# Photocatalytic C−H Azolation of Arenes Using Heterogeneous Carbon Nitride in Batch and Flow

**DOI:** 10.1002/cssc.202101767

**Published:** 2021-10-22

**Authors:** Zhenghui Wen, Ting Wan, Arjun Vijeta, Carla Casadevall, Laura Buglioni, Erwin Reisner, Timothy Noël

**Affiliations:** ^1^ Flow Chemistry Group Van't Hoff Institute for Molecular Sciences (HIMS) Universiteit van Amsterdam (UvA) Science Park 904 1098 XH Amsterdam The Netherlands; ^2^ Yusuf Hamied Department of Chemistry University of Cambridge Lensfield Road Cambridge CB2 1EW United Kingdom; ^3^ Department of Chemical Engineering and Chemistry Sustainable Process Engineering Eindhoven University of Technology P.O. Box 513 5600 MB Eindhoven The Netherlands

**Keywords:** azolation, carbon nitride, flow chemistry, heterogeneous catalysis, photocatalysis

## Abstract

The functionalization of aryl C(sp^2^)−H bonds is a useful strategy for the late‐stage modification of biologically active molecules, especially for the regioselective introduction of azole heterocycles to prepare medicinally‐relevant compounds. Herein, we describe a practical photocatalytic transformation using a mesoporous carbon nitride (mpg‐CN_*x*_) photocatalyst, which enables the efficient azolation of various arenes through direct oxidation. The method exhibits a broad substrate scope and is amenable to the late‐stage functionalization of several pharmaceuticals. Due to the heterogeneous nature and high photocatalytic stability of mpg‐CN_*x*_, the catalyst can be easily recovered and reused leading to greener and more sustainable routes, using either batch or flow processing, to prepare these important compounds of interest in pharmaceutical and agrochemical research.

## Graphical abstract

**Green and versatile**! The photocatalytic C(sp^2^)−H azolation of arenes using heterogeneous carbon nitride is reported. This strategy exhibits a broad substrate scope and is amenable to the late‐stage functionalization of several pharmaceuticals. The catalyst can be easily recovered and reused leading to greener and more sustainable routes.

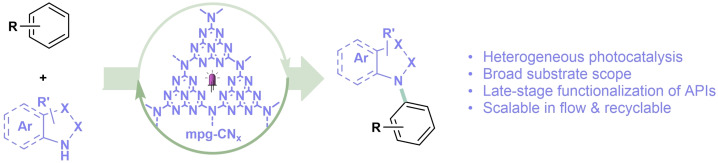

## Introduction

The ability to functionalize organic molecules is the central tenet of synthetic organic chemistry.^[1]^ Historically, this was achieved by converting functional groups in a step‐wise and selective fashion to gradually build up molecular complexity (so‐called synthon approach).^[2]^ As a leading example, transition metal‐catalyzed cross‐coupling served as a powerful transformation to construct carbon‐carbon and carbon‐heteroatom bonds by matching nucleophiles with suitable electrophiles.^[3]^ Key for the success of cross‐coupling chemistry is the access to molecular fragments with this appropriate built‐in functionality, requiring often laborious multi‐step synthetic protocols.

The need to pre‐install such functional handles prevents these classical strategies from being practical for the late‐stage functionalization (LSF) of biologically active molecules and materials. LSF is an ideal strategy to rapidly prepare a diverse set of analogues, which are useful to study structure‐activity relationships, to block metabolic sites or to prepare oxidized metabolites.^[4]^ As a prime example of LSF, C−H bond activation has enabled chemists to purposely modify C−H bonds in organic molecules.^[5]^ In many cases, these modifications occur in the proximity of an existing functional group,^[6]^ so‐called directing group, which allows the active catalyst to navigate to a certain C−H bond at which the actual functionalization step occurs.^[7]^ However, the most versatile strategy is the undirected and selective C−H bond functionalization, which is far more challenging.^[8]^

Several notable strategies to functionalize C(sp^2^)−H bonds utilize electro‐,^[9]^ photo‐^[10]^ or photoelectrochemical^[11]^ oxidation of electron‐rich arenes to give the corresponding radical cations, which can be subsequently attacked by various nucleophiles. Nicewicz and co‐workers have shown that such a strategy can be used for the site‐selective C(sp^2^)−H amination of arenes with azoles.^[12]^ A photoelectrocatalytic approach was developed by Hu and co‐workers using hematite as photoanode and allowed to avoid the use of homogeneous photocatalysts.^[13]^ Recently, Wang, König and co‐workers developed a metal‐free semiconductor strategy using hexagonal boron carbon nitride as a photocatalyst.^[14]^ Despite these seminal advances, a general protocol displaying a broad substrate scope and using an easy‐to‐recover photocatalyst remains a challenge.

In this line, graphitic carbon nitride (CN_*x*_), a polymeric material composed of heptazine units, has emerged as one of the most promising heterogeneous photocatalysts.^[15]^ This material has gathered interest for being inexpensive, robust and easy to prepare, along with possessing outstanding thermal, chemical and photostability. CN_*x*_ has a broad absorption in the UV/Vis region and an optical band gap of approximately 2.7 eV, making it suitable for various applications, including solar fuels production, environmental remediation^[16]^ and organic transformations.^[17]^ The physical and chemical properties of graphitic carbon nitride can be easily tuned by simple modifications. One of its morphologically modified derivatives, known as mesoporous graphitic carbon nitride (mpg‐CN_*x*_), with a VB of −1.1 V and CB of +1.6 V vs SCE is particularly attractive for catalytic applications due to its large surface area.^[18]^

Herein, we describe the use of mpg‐CN_*x*_ as a heterogeneous photocatalyst to enable the photocatalytic C−H azolation of arenes. The scope of the transformation is broad, enabling both early‐stage modification of arenes and late‐stage functionalization of drug molecules. Due to its heterogeneous nature, mpg‐CNx can be recovered easily and recycled without loss of its photocatalytic activity. Moreover, the photocatalyst can be loaded in a packed‐bed reactor, which allows easy separation of the product from the photocatalyst and enables scale‐up as well as higher‐throughput of the targeted transformation in flow.

## Results and Discussion

**Figure 1:**
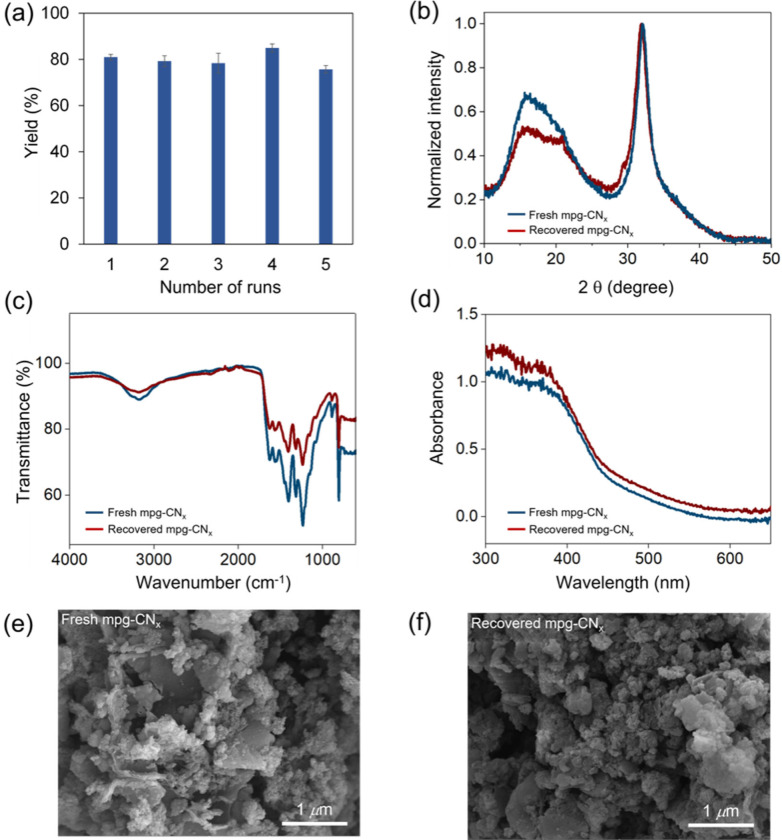
Recovery and recyclability of mpg‐CN_*x*_. a) recyclability of mpg‐CN_*x*_ for the photocatalytic coupling reaction between mesitylene and pyrazole at the standard conditions (Yields were determined by ^1^H NMR spectroscopy with pyrazine as external standard). b) pXRD patterns of fresh and recovered mpg‐CN_*x*_ after 4 cycles. c) ATR‐IR spectra of fresh and recovered mpg‐CN_*x*_ after 4 cycles. d) UV/Vis DRS of fresh and recovered mpg‐CN_*x*_ after 4 cycles. e) SEM image of the material before. f) SEM image after 4 photocatalytic cycles.

The mpg‐CN_*x*_ photocatalyst was synthesized as previously reported by heating cyanamide as a precursor with silica as a hard template in air at 550 °C for 4 h, followed by silica etching using aqueous ammonium difluoride.^[16b]^ The material was characterized by powder X‐ray diffraction (pXRD), attenuated total reflectance infrared (ATR‐IR), UV/Vis diffuse reflection spectroscopy (DRS), and scanning electron microscopy (SEM) to confirm the composition and morphology of mpg‐CN_*x*_ (Figure 1). The stacking and in‐plane periodicity of this polymeric material can be confirmed from the pXRD peaks at 27° and 12°, respectively. The ATR‐IR peak at 804 cm^−1^ confirms the characteristic vibration of the heptazine core. The mpg‐CN_*x*_ exhibits the expected UV/Vis absorption and SEM images show the mesoporosity of the material.

With sufficient quantities of mpg‐CN_*x*_ photocatalyst in hand, we commenced our investigations with the coupling reaction between mesitylene and pyrazole as benchmark (Table 1). After an extensive evaluation of different reaction conditions (see the Supporting Information), we identified optimal reaction conditions outlined in Table 1, entry 1. The cross‐coupling between mesitylene and pyrazole could be realized in the presence of mpg‐CN_*x*_ and adding potassium persulfate as the oxidant. Control experiments revealed that the reaction required a photocatalyst, light and oxidants to yield the targeted product **1** (Table 1, entries 2–6). It is worth mentioning that K_2_S_2_O_8_, in the absence of photocatalyst, can also enable C(sp^2^)−H functionalization reactions,^[19]^ albeit at low yields under the employed cross‐coupling reaction conditions (Table 1, entry 2). Finally, the reaction was subjected to UV−A light (*λ*=365 nm LEDs) to obtain optimal results but could also be realized with visible light (Table 1, entries 1, 8). This observation sets mpg‐CN_*x*_ apart from classical semiconductor‐based photocatalysts, such as TiO_2_ (Table 1, entries 9, 10).^[20]^

**Table 1:** Control reactions for optimized experimental conditions for the photocatalytic C−H azolation of arenes.^[a]^

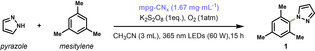		
		
Entry	Deviation from above	Yield **1** (%)^[b]^
1	none	82 (80)^[c]^
2	No mpg‐CN_*x*_	7
3	No K_2_S_2_O_8_	51
4	No O_2_; under N_2_ atmosphere	39
5	No K_2_S_2_O_8_, No O_2_	n.d.
6	Na_2_S_2_O_8_ instead of K_2_S_2_O_8_	61
7	No light	n.d.
8	Blue light	60
9	TiO_2_ instead of mpg‐CN_x_	41
10	TiO_2_ instead of mpg‐CN_x_ irradiated with blue light	n.d.

[a] Reaction conditions: pyrazole (0.2 mmol), mesitylene (6 equiv.), mpg‐CN_*x*_ (1.67 mg mL^−1^), K_2_S_2_O_8_ (1 equiv.), O_2_ (1 atm), CH_3_CN (3 mL), room temperature, 365 nm UV LEDs (60 W), 15 h. [b] Yields were determined by ^1^H NMR spectroscopy with pyrazine as external standard. [c] Isolated yield. n.d.=not detected.

**Scheme 1:**
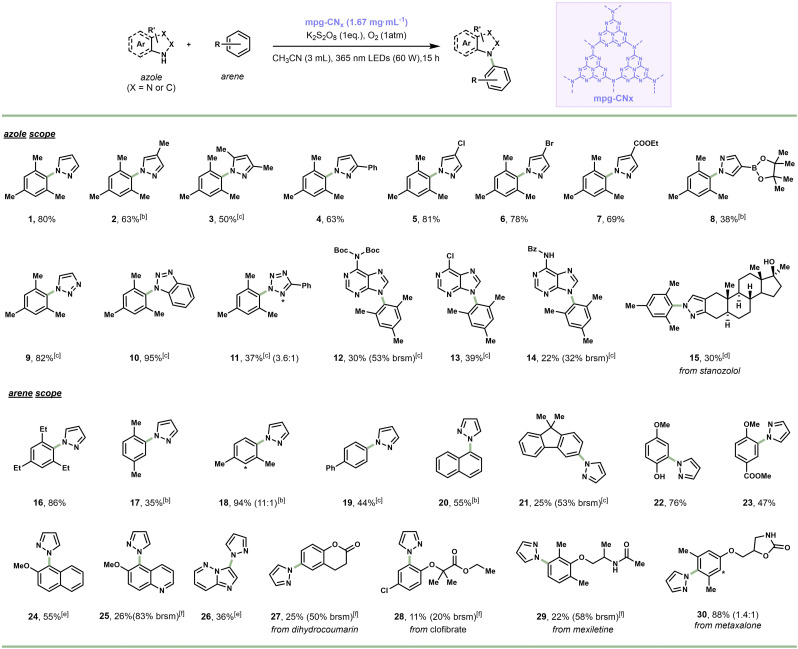
Substrate scope of the photocatalytic C−H azolation of arenes using mpg‐CN_*x*_ as a heterogenous photocatalyst. [a] Reaction conditions: Azole (0.6 mmol), arene (6 equiv.), mpg‐CN_*x*_ (1.67 mg mL^−1^), O_2_ (1 atm), K_2_S_2_O_8_ (1 equiv.), 365 nm LEDs (60 W), CH_3_CN (3 mL), rt, 15 h; [b] Arene (18 equiv.); [c] Arene (18 equiv.), 24 h; [d] Arene (18 equiv.), 40 h; [e] 24 h; [f] 40 h. Brsm: based on remaining starting material.

With the optimal reaction conditions established, we evaluated the generality of our mpg‐CN_*x*_‐enabled photocatalytic C−H azolation of arenes (Scheme 1). Mesitylene as a model substrate was combined with various alkyl‐ and aryl‐bearing pyrazoles, generating the coupled products in good yields (**1**–**4**, 50–80 %). The reaction tolerated also halogenated pyrazoles, which afforded the targeted compounds in excellent yields (**5**, **6**, 78–81 %); these halogenated compounds can serve as suitable electrophiles in classical cross‐coupling chemistry.^[21]^ Also pyrazoles with electron‐withdrawing functional groups (e. g., ester) were accommodated in the transformation (**7**, 69 %). Notably, a pyrazole containing a boronic acid pinacol (Bpin) ester functionality was successfully coupled under the reaction conditions (**8**, 38 % yield); the presence of the Bpin functionality should enable further diversification in other catalytic transformations, such as Suzuki‐Miyaura^[22]^ or Chan‐Evans‐Lam coupling reactions.^[23]^ Other azoles, including triazoles (**9**–**10**, 82–95 %), tetrazole (**11**, 37 %) and purines (**12**–**14**, 22–39 %), were found to serve as competent nucleophiles despite some substrates (purines) having a limited solubility. Remarkably, we also managed to functionalize the anabolic steroid stanozolol to yield adduct **15** in synthetically useful quantities (**15**, 30 %).

Next, we investigated the ability of the developed photocatalytic protocol to functionalize various arenes using pyrazole as the benchmark coupling partner (Scheme 1). Various alkylated and arylated arenes could be coupled with pyrazole in good to excellent yields (**16**–**21**, 25–94 %). These examples also show that the photocatalytic procedure is not particularly sensitive to steric hindrance (e. g., **1**, **16**, **20**). Also, electron‐rich arenes can be readily coupled with pyrazole (**22**–**24**, 47–76 %). Heteroarenes as drug‐like moieties, such as quinoline and imidazo[1,2‐b]pyridazine, are suitable reaction partners as well, yielding the targeted compounds in medicinally‐relevant quantities (**25**, **26**, 26–36 %).

Given the pharmaceutical relevance of the developed photocatalytic protocol,^[24]^ we further sought to convert several medicinal scaffolds with pyrazole as nucleophile. As shown in Scheme 1, dihydrocoumarin, clofibrate, mexiletine and metaxalone could be readily engaged in the photocatalytic C−H azolation coupling procedure, yielding the corresponding adducts (**27**–**30**, 11–88 %). These results underscore the potential of our transformation to enable the late‐stage functionalization of important Active Pharmaceutical Ingredients (APIs).

One of the major advantages of heterogeneous photocatalysis is the ease of photocatalyst recovery, facilitating the purification of APIs to meet the associated stringent purity requirements.^[25]^ The recovered catalyst can subsequently be reused on the proviso that its activity is preserved and the heterogeneous catalyst is not leached into solution.^[26]^ For our benchmark coupling reaction between mesitylene and pyrazole (Table 1, entry 1), we showed that mpg‐CN_*x*_ could be recycled at least 4 times without any noticeable loss of photocatalytic activity (Figure 1A). It should be further noted that the entire substrate scope was performed with a single batch of mpg‐CN_*x*_, which was recovered after each experiment (Scheme 1). Also pXRD, ATR‐IR, UV/Vis and SEM images revealed no apparent changes in the nature of the photocatalyst before and after photocatalysis, further substantiating the high stability of mpg‐CN_*x*_ (Figure 1). Finally, we also investigated several batches of mpg‐CN_*x*_ photocatalyst and obtained for three different batches identical kinetic profiles (see the Supporting Information), demonstrating excellent inter‐batch reproducibility.

**Scheme 2:**
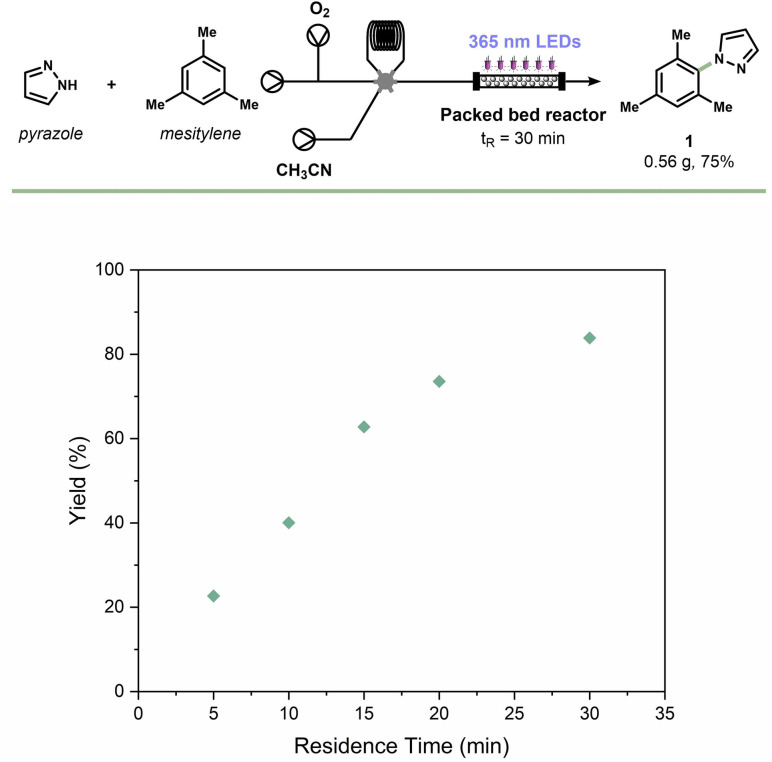
Scale up and reaction kinetics of the C−H azolation of arenes in a continuous‐flow packed bed reactor filled with mpg‐CN_*x*_.

These results show that the catalyst is a stable and promising candidate for prolonged use during a scale‐up experiment in a continuous‐flow reactor.^[27]^ Essentially two strategies can be distinguished for handling solids in flow: suspension flow^[28]^ or immobilization inside the reactor.[^18c^, ^29^] We selected the latter approach as it avoids the need for catalyst recovery/recycling strategies and is technologically straightforward to implement. After packing a capillary (PFA, 2 mm I.D. ×3 mm O.D.) with mpg‐CN_x_, a segmented flow of the liquid reagents and oxygen gas was directed over the photocatalytic bed which was subjected to 365 nm LED irradiation (see Scheme S3 in the Supporting Information). Due to the high light intensity in capillary microreactors,^[30]^ we observed a significant acceleration of the reaction kinetics leading to a full conversion within only 30 minutes residence time (Scheme 2). Using these optimized flow conditions, 0.56 g of the target compound **1** could be readily obtained with a high turnover frequency (TOF) valve (835.2 μmol g^−1^ h^−1^) of the mpg‐CN_*x*_ photocatalyst.

**Scheme 3:**
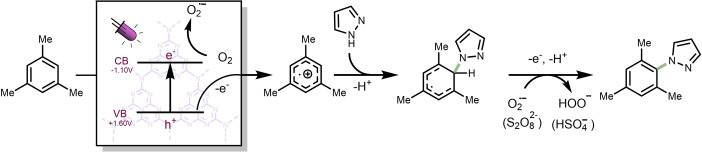
Plausible mechanism for the photocatalytic C−H azolation of arenes using heterogeneous carbon nitride.

Based on our experimental observations, a plausible mechanistic rationale is suggested in Scheme 3. Upon light absorption, mpg‐CN_*x*_ generates an electron‐hole pair.^[31]^ The arene (e. g., mesitylene, *E*_ox_=+2.05 V vs. SCE)^[32]^ is subsequently oxidized via a single electron transfer from the arene to the hole in the valence band of mpg‐CN_*x*_. The corresponding arene radical cation is prone to nucleophilic attack from the azole coupling partner. This event is followed by another single electron oxidation and proton removal, which rearomatizes the arene yielding the targeted cross‐coupled product. Oxygen and K_2_S_2_O_8_ play a role as electron acceptors from the conduction band of mpg‐CN_*x*_ and as terminal oxidant in the rearomatization process.

## Conclusions

In summary, we have developed a practical protocol for the photocatalytic C−H azolation of arenes using heterogeneous mesoporous carbon nitride. The reaction displays a broad scope including both early‐ and late‐stage functionalization of aryl C(sp^2^)−H bonds. mpg‐CN_*x*_ is shown to be highly stable under the developed reaction conditions and can be recycled several times without noticeable reduction in photocatalytic activity and can be used under continuous‐flow reaction conditions. Due to the heterogeneous and metal‐free nature of this photocatalyst, we expect this method to be of great added value to the synthetic community, both in academia and industry.

## Experimental Section

### Catalyst preparation

mpg‐CN_*x*_ was synthesized and characterized (ATR‐IR, pXRD and UV/Vis DRS) according to a previously reported procedure.[^18c^, ^33^] Cyanamide (3 g) was dissolved in a 40 wt.% dispersion of SiO_2_ (7.5 g, Ludox SM) in water and stirred at 60 °C overnight. The reaction mixture was then heated at a rate of 2.3 °C min^−1^ over 4 h to reach a temperature of 550 °C, and then kept at this temperature for an additional 4 h. The resulting brown‐yellow powder was treated with NH_4_HF_2_ (4 M; note that handling requires extreme caution) for 24 h to remove the silica template. The powder was then centrifuged at 9000 rpm and washed with distilled water (3×) and ethanol (2×). Finally, the powders were dried overnight at 70 °C under vacuum.

### General procedure 1 (GP1) for batch conditions

Three oven dried vials equipped with a stirring bar were charged with mpg‐CN_*x*_ (5 mg, 1.67 mg mL^−1^), K_2_S_2_O_8_ (54 mg, 0.2 mmol, 1 equiv.), azole (0.2 mmol, 1 equiv.) and arene (1.2 mmol, 6 equiv.). Subsequently, CH_3_CN (3 mL) was added and the vials were sealed with caps. After sonicating for 5–10 min, O_2_ was bubbled into the three vials for 5 min. The vials were then irradiated in the batch reactor (see Figure S1 in the Supporting Information) at room temperature with rapid stirring (1500 rpm) under an oxygen atmosphere. When the reaction was completed (15‐40 h), the crude reaction mixtures were centrifuged at 5000 rpm for 5 min, followed by careful separation of the liquid phase. The catalyst was washed twice with CH_3_CN. The combined organic phase was dried under reduced pressure and purified by flash column chromatography on a Biotage® Isolera Four system affording the product, which was characterized by ^1^H NMR, ^13^C NMR, and HRMS.

### General procedure 2 (GP2) for flow conditions

To a 5 mL oven‐dried volumetric flask pyrazole was added (20 mg, 0.06 M), together with mesitylene (6 equiv., *d*=0.864 g mL^−1^, 166.8 μL) and CH_3_CN (up to 5 mL). The reaction mixture was transferred into a 20 mL Erlenmeyer flask, which served as a storage vessel to be pumped into the reactor by a peristaltic pump. The oxygen flow was set using a mass flow controller and it was subsequently mixed with the liquid feed via a PEEK T‐mixer (IDEX, P‐714, inner diameter 1 mm). The combined gas‐liquid feed was pumped into a 10 mL pre‐reactor (PFA capillary tubing, 1.59 mm I.D., equipped with two switching valves, see Figure S3 in the Supporting Information) at 10 mL min^−1^ and 2 mL min^−1^, respectively. When the loop was completely full, it was closed using the two switching valves and connected to a HPLC pump via a six‐way valve (see Figure S4 in the Supporting Information). Next, the inlet valve was opened, and the loop was pressurized. After pressurization, the outlet valve was also opened to pump the reaction mixture into the mpg‐CN_*x*_ packed‐bed reactor for the desired residence time (5–30 min). The setup allowed adding CH_3_CN through the six‐way valve to flush the reaction mixture out. The collected solution was dried under reduced pressure and pyrazine (8 mg, 0.1 mmol) was added as external standard. Finally, ^1^H‐NMR analysis was performed. The scheme of the full flow set‐up is shown in Figure S5 in the Supporting Information.

## Conflict of interest

The authors declare no conflict of interest.

## Supporting information

As a service to our authors and readers, this journal provides supporting information supplied by the authors. Such materials are peer reviewed and may be re‐organized for online delivery, but are not copy‐edited or typeset. Technical support issues arising from supporting information (other than missing files) should be addressed to the authors.

Supporting Information
