# Supplementary material for: Photocatalytic C−H Azolation of Arenes Using Heterogeneous Carbon Nitride in Batch and Flow
Source: ChemSusChem. 2021 Oct 22;14(23):5265–70. doi: 10.1002/cssc.202101767 (PMC9298336; doi:10.1002/cssc.202101767)
Supplement: Supplementary file 1 — Supporting Information [file CSSC-14-5265-s001.pdf]

ChemSusChem

Supporting Information

Photocatalytic C─H Azolation of Arenes Using Heterogeneous Carbon Nitride in Batch and Flow

Zhenghui Wen, Ting Wan, Arjun Vijeta, Carla Casadevall, Laura Buglioni, Erwin Reisner,* and Timothy Noël*© 2021 The Authors. ChemSusChem published by Wiley-VCH GmbH. This is an open access article under the terms of the Creative Commons Attribution License, which permits use, distribution and reproduction in any medium, provided the original work is properly cited.

Supporting Information

Photocatalytic C–H Azolation of Arenes using Heterogeneous Carbon Nitride in batch and flow

Zhenghui Wen,[a] Ting Wan,[a] Arjun Vijeta,[b] Carla Casadevall,[b] Laura Buglioni,[c] Erwin Reisner,*[b] Timothy Noël*[a]

[a] Flow Chemistry Group, van 't Hoff Institute for Molecular Sciences (HIMS), Universiteit van Amsterdam (UvA), Science Park 904, 1098 XH, Amsterdam, The Netherlands.

[b] Yusuf Hamied Department of Chemistry, University of Cambridge, Lensfield Road, Cambridge CB2 1EW, United Kingdom.

[c] Department of Chemical Engineering and Chemistry, Sustainable Process Engineering, Eindhoven University of Technology, P.O. Box 513, 5600 MB Eindhoven, The Netherlands.

Table of Contents

1. General information 3
2. Catalyst preparation 5
3. Reactor Design 5
3.1 Homemade setup for batch experiments 5
3.2 Vapourtec UV-150 6
4. General procedure 8
4.1 General procedure 1 (GP1) for batch conditions: 8
4.2 General procedure 2 (GP2) for flow conditions: 8
5. Reaction Optimization 9
5.1 General experimental procedure for screening experiments 9
5.2 Solvent screening 10
5.3 Oxidant screening 11
5.4 mpg-CN_x loading screening 12
5.5 Mesitylene equivalent screening 13
5.6 Light source screening 14
6. Light-Dependence Experiments 14
7. Stabilization test of different batches of mpg-CNx 17
8. Substrate preparation 18
9. Characterization data 19
10. NMR spectra 29
11. Reference 61

1. General information

All reagents and solvents were used as received without further purification, unless stated otherwise. Reagents and solvents were bought from Sigma Aldrich, TCI and Fluorochem. Technical solvents were bought from Biosolve and were used as received. LED strips (365 nm, 5 m, 240 SMD2835 LEDs) were purchased from LuxaLight. For batch experiments, the LED strip was cut in half and 2.5 m of LED strip were coiled in a 3D-printed (PLA) reactor with an inner diameter of 12.5 cm. For flow experiments, Vapourtec UV-150 device was used, equipped with 150 W 365 nm LEDs. All capillary tubing and microfluidic fittings were purchased from IDEX Health & Science. Mass flow controller was purchased from Bronkhorst. Disposable syringes were from BD Discardit II® or NORM-JECT®, purchased from VWR Scientific. The pperistaltic pump was bought from Masterflex Ismatec, model Miniflex dual-channel pump 78018-42. HPLC pump was purchased by Shimadzu model LC-20AD. Product isolation was performed automatically by a Biotage® Isolera Four, with Biotage® SNAP KP-Sil 10 or 25 g flash chromatography cartridges. TLC analysis was performed using Silica on aluminium foils TLC plates (F254, Supelco Sigma-Aldrich™) with visualization under ultraviolet light (254 nm and 365 nm) or cerium ammonium molybdate stain (CAM). ^{1}H (400 MHz) and ^{13}C (100 MHz) spectra were recorded on ambient temperature using a Bruker-Avance 400. ^{1}H NMR spectra are reported in parts per million (ppm) downfield relative to $CDCl_3$ (7.26 ppm) and all ^{13}C NMR spectra are reported in ppm relative to $CDCl_3$ (77.16 ppm), unless stated otherwise. In the NMR spectra the following abbreviations were used to describe the multiplicity: s = singlet, d = doublet, t = triplet, q = quartet, p = pentet, h = hextet, hept = heptet, m = multiplet, dd = double doublet, td = triple doublet. NMR data was processed using the MestReNova 12.0 software package. Known products were characterized by comparing to the corresponding ^{1}H NMR and ^{13}C NMR from literature. Melting points were recorded on a Buchi M-565 melting point apparatus. High-resolution mass spectra (HRMS) were recorded on an AccuTOF LC, JMS-T100LP Mass spectrometer (JEOL, Japan). The X-ray diffraction (XRD) patterns were obtained with a Bruker-AXS D2 Phaser powder X-ray diffractometer in Bragg-Brentano geometry using Co Kα = 1.78897 Å, operated at 30 kV. Infrared spectra of the samples were measured using a PerkinElmer Spectrum 100 spectrometer equipped with an ATR sampling accessory. UV-vis diffuse reflection spectroscopy (DRS) of powder material was carried out on an Agilent Cary machine. Scanning electron microscopy (SEM) images were collected on a high performance MIRA3 FEG-SEM system that features a high brightness

Schottky emitter at an accelerating voltage of 5 kV. The names of all products were generated using the PerkinElmer ChemDraw 20.1 software package.

2. Catalyst preparation

mpg-CN_x was synthesized and characterized by ATR-IR, pXRD and UV-Vis DRS according to a procedure previously reported.[1] For mpg-CN_x, cyanamide (3 g) was dissolved in a 40 wt.% dispersion of SiO_2 (7.5 g, Ludox SM) in water, stirring at 60 °C overnight. The resulting solution was then heated at a rate of 2.3 °C·min^{-1} over 4 h to reach a temperature of 550 °C, and then kept at this temperature for further 4 h. The resulting brown-yellow powder was treated with 4 M NH_4HF_2 for 24 h in order to remove the silica template. The powders were then centrifuged at 9000 rpm and washed three times with distilled water and twice with ethanol. Finally, the powders were dried overnight at 70 °C under vacuum.

3. Reactor Design

3.1 Homemade setup for batch experiments

All the optimization experiments and scope in batch were performed with our homemade photoreactor (**Figure S1**).

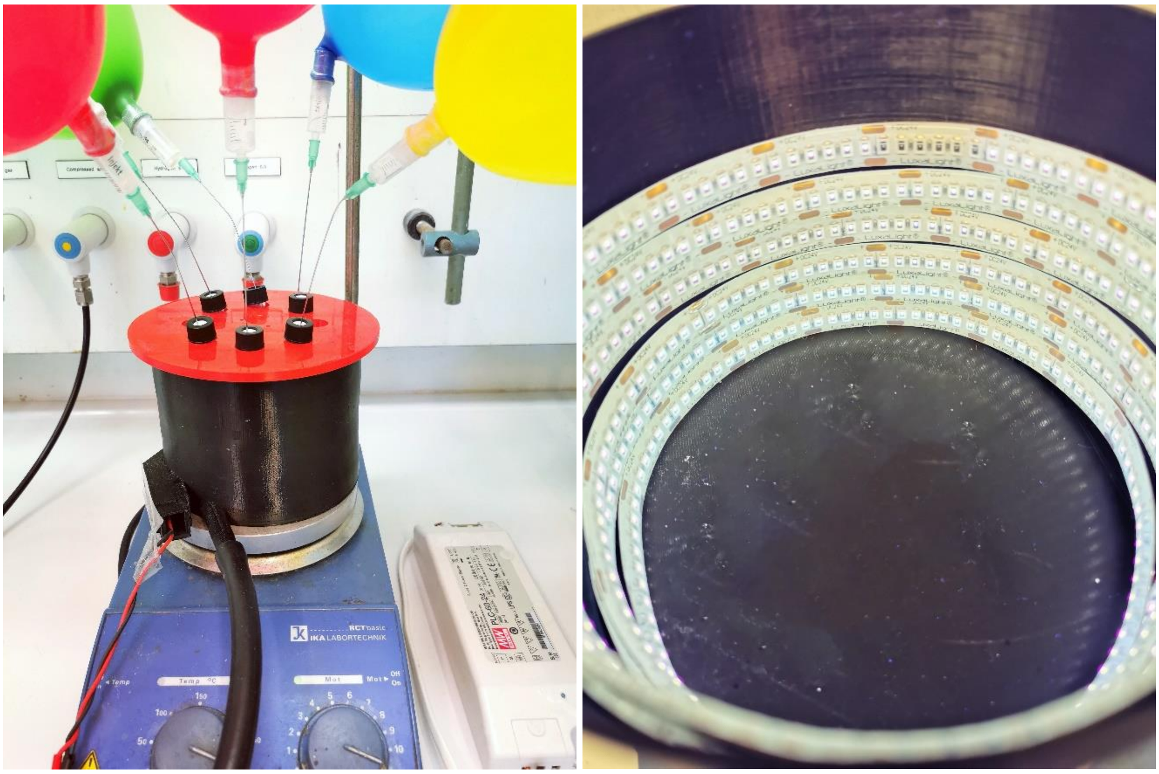

Figure S1. Left: 3D printed reactor in use. **Right:** the reactor was covered with reflective tape and contains 2.5 m of near-UV LED strip (365 nm, 60W). The reactor has an inner diameter of 12.5 cm.

3.2 Vapourtec UV-150

For the experiments in flow, a Vapourtec UV-150 photochemical reactor was used, equipped with 150 W LED (365 nm). A packed-bed reactor (**Figure S2**) was prepared following a reported procedure.[2] A 3 wt.% mpg-CN_x mixture was prepared by gently grinding 100 mg of mpg-CN_x, 540 mg of $K_2S_2O_8$ and 2.8 g of a 1:1 mixture of 212-300 µm and 425-600 µm diameter glass beads in an agate mortar. This mixture was then vigorously stirred in a vortex generator for 30 s. One end of a 1 m long PFA tube (3 mm o.d., 2 mm i.d.) was plugged with Celite and cotton filter, and the tube was then filled approximately up to a height of 5 cm with 212-300 µm diameter glass beads. The photocatalyst-containing mixture was packed into the tube, and the other end was then closed with a cotton filter. The entry and exit point of the reactor were connected to 1/8" o.d. 1/4"-28 flat bottom flangeless fittings, for facile in-line connection. The volume of the photoreactor, V_r, was calculated using the difference in mass of the packed 'dry' reactor (m_1) and the mass of the packed reactor when filled with the solvent of choice (m_2.) (Equation S1).

$$V_r = \frac{\Delta m}{\rho_{solv.}} = \frac{(m_2 - m_1)}{\rho_{solv.}} \qquad \text{(Equation S1)}$$

where $\rho_{\mathrm{solv.}}$ denotes the solvent density. The calculated volume of the packed-bed reactor was 1.74 mL.

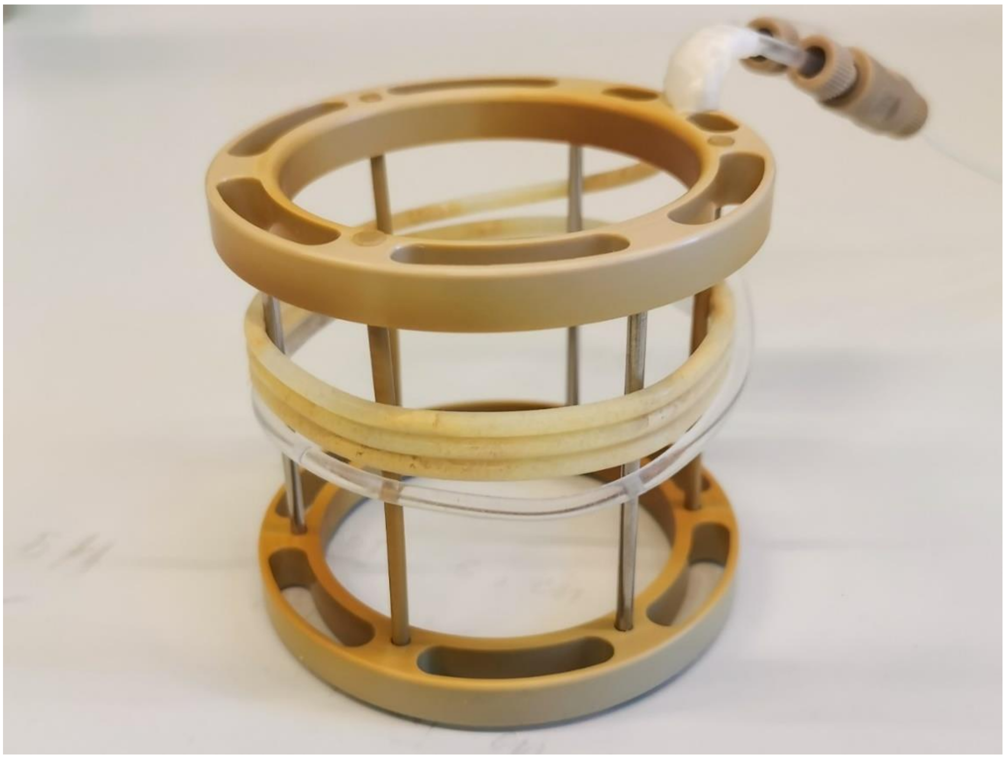

Figure S2. Picture of packed bed reactor with Vapourtec reactor support.

The setups of the flow experiments are shown in **Figure S3** and **S4**.

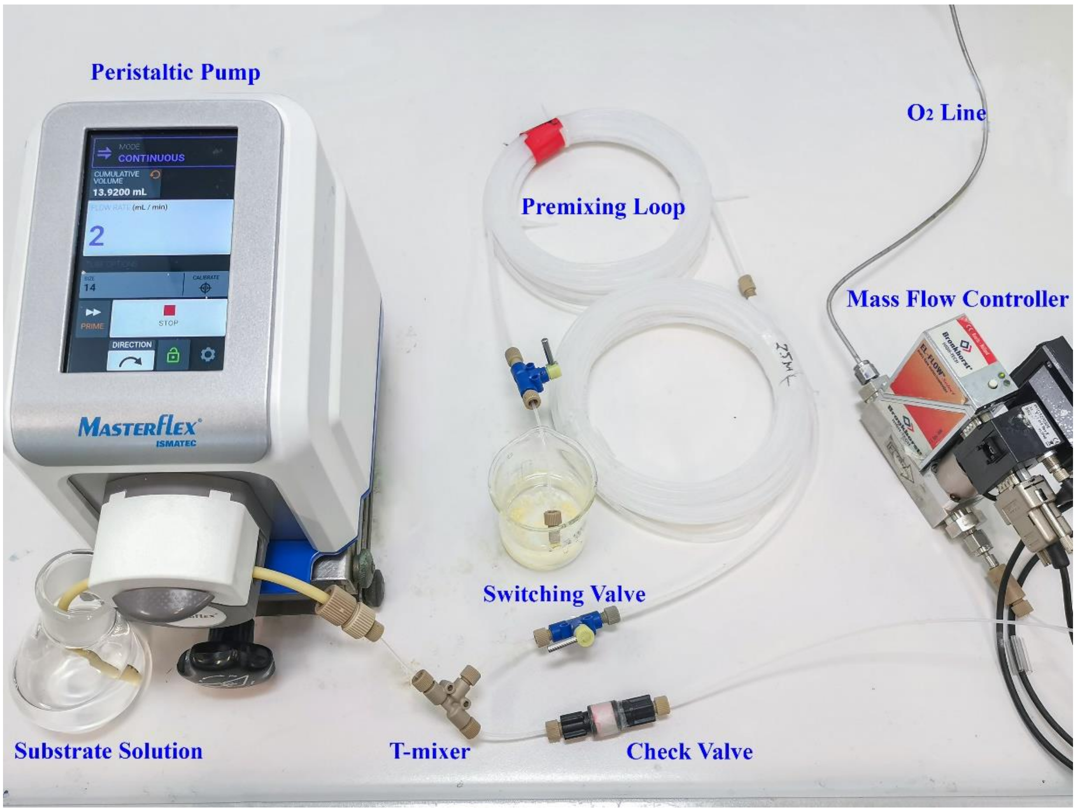

Figure S3. Picture of the flow setup for premixing of substrate solution and O_2.

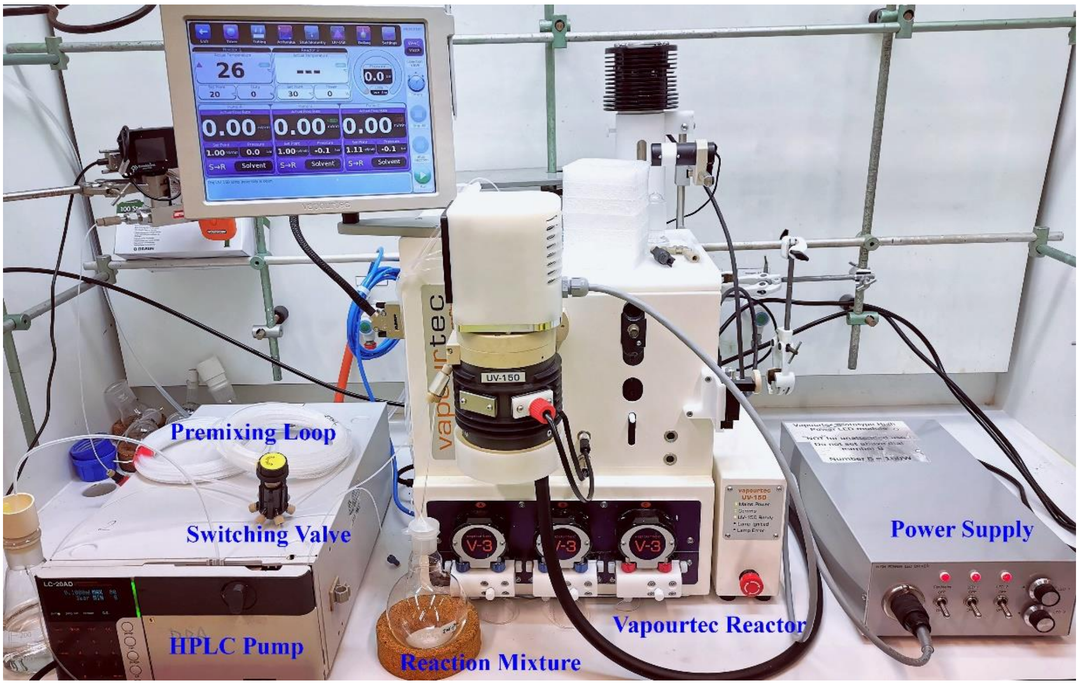

Figure S4. Picture of the setup of experiments in flow.

4. General procedure

4.1 General procedure 1 (GP1) for batch conditions:

Three oven dried vials (13 x 100 mm) equipped with a stirring bar were charged with mpg-CN_x (5 mg, 1.67 mg mL^{-1}), $K_2S_2O_8$ (54 mg, 0.2 mmol, 1 equiv.), azole (0.2 mmol, 1 equiv.) and arene (1.2 mmol, 6 equiv.). Subsequently, CH_3CN (3 mL) was added and the vials were sealed with caps. After sonicating for 5-10 min, O_2 was bubbled into the three vials for 5 min. The vials were then irradiated in the batch reactor (described above) at room temperature with rapid stirring (1500 rpm) under an oxygen atmosphere. When the reaction was over (15-40 h), the crude reaction mixtures were centrifuged at 5000 rpm for 5 min and then the liquid phase was carefully separated. The catalyst was washed twice with CH_3CN. The combined organic phase was dried under reduced pressure and purified by flash column chromatography on a Biotage® Isolera Four system affording the product, which was characterized by ^1H NMR, ^{13}C NMR, and HRMS.

4.2 General procedure 2 (GP2) for flow conditions:

To a 5 mL oven-dried volumetric flask pyrazole was added (20 mg, 0.06 M), together with mesitylene (6 equiv., d = 0.864 g·mL^{-1}, 166.8 μL) and CH_3CN (up to 5 mL). The reaction mixture was transferred into a 20 mL Erlenmeyer flask to be pumped with a peristaltic pump. Oxygen flow was set using a mass flow controller and then it was mixed with the liquid feed via a PEEK T-mixer (IDEX, P-714, inner diameter 1 mm). The gas and the liquid feed were pumped into a 10 mL pre-reactor (PFA capillary tubing, 1.59 mm I.D., equipped with two switching valves, see **Figure S3**) at 10 mL min^{-1} and 2 mL min^{-1}, respectively. When the loop was completely full, it was closed using the two switching valves and connected to a HPLC pump via a six-way valve (see **Figure S4**). Next, the inlet valve was opened, and the loop was pressurized. After pressurization, the outlet valve was opened as well, to pump the reaction mixture into the packed-bed reactor for the desired residence time (5-30 min). It was possible to add CH_3CN through the six-way valve to elute the reaction mixture out. The collected volume was dried under reduced pressure and pyrazine (8 mg, 0.1 mmol, external standard) was added. Finally, ^1H-NMR analysis was performed. The scheme of the full flow set-up is shown in **Figure S5**.

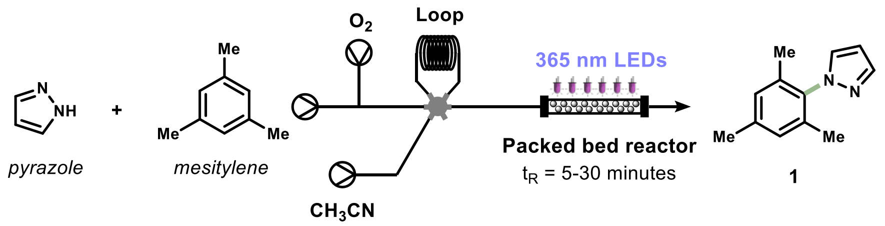

Figure S5. Schematic representation of the flow set-up.

5. Reaction Optimization

5.1 General experimental procedure for screening experiments

Following the **GP1** (see section 4.1), mpg-CN_x (5 mg, 1.67 mg/mL), an oxidant (0.2 mmol, 1 equiv.), pyrazole (13.6 mg, 0.2 mmol, 1 equiv.) and mesitylene (1.2 mmol, 6 equiv.) were mixed in a solvent (3 mL). After 15 h, the reaction crude was analyzed by ^{1}H NMR using pyrazine as an external standard (0.1 mmol, 0.5 equiv., 8 mg). For a representative NMR spectrum, see **Figure S6**.

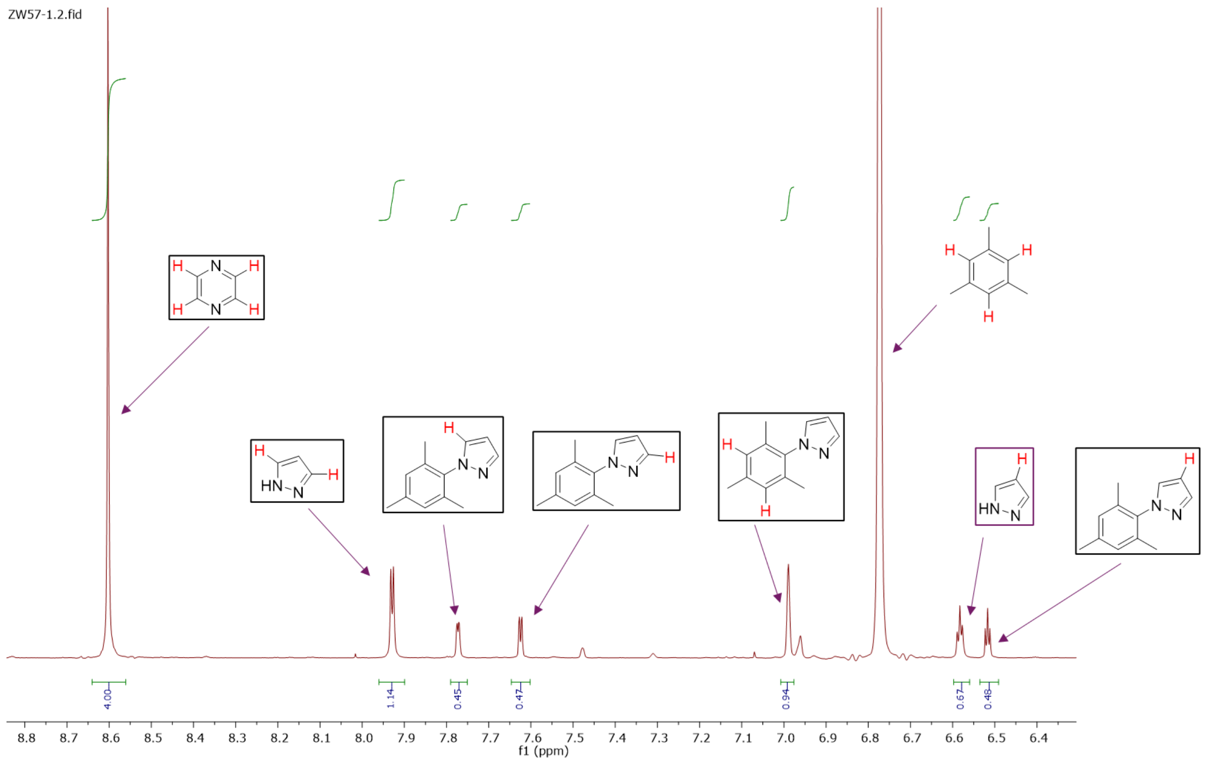

Figure S6. Example of ^{1}H-NMR spectra for determining NMR yields.

5.2 Solvent screening

Table S1. Solvent screening. [a]

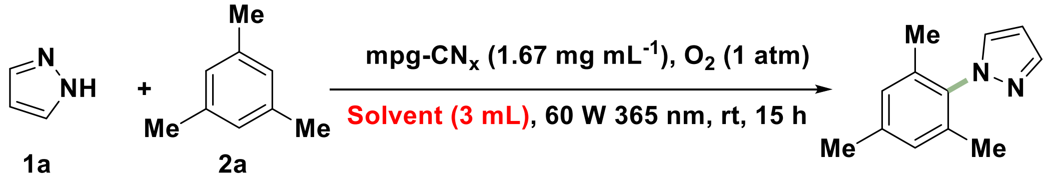

Entry	Solvent (3 mL)	Conversion (%)[b]	Yield (%)[c]
1	Anhydrous CH_3CN	32	31
2	Methanol	7	5
3	DCM	8	5
4	DMAc	29	23
5	DCE	6	5
6	Acetone	51	23
**7**	**CH_3CN**	**39**	**33**

[a] Reaction conditions: pyrazole (0.2 mmol), mesitylene (0.6 mmol, 3 equiv.), O_2 (1 atm), mpg-CN_x (5 mg, 1.67 mg mL^{-1}), solvent (3 mL), 60 W 365 nm UV light at room temperature for 15 h. [b] The conversion of pyrazole was determined by ^{1}H-NMR using pyrazine as external standard. [c] The yield was determined by ^{1}H-NMR using pyrazine as external standard.

5.3 Oxidant screening

Table S2. Oxidant screening. [a]

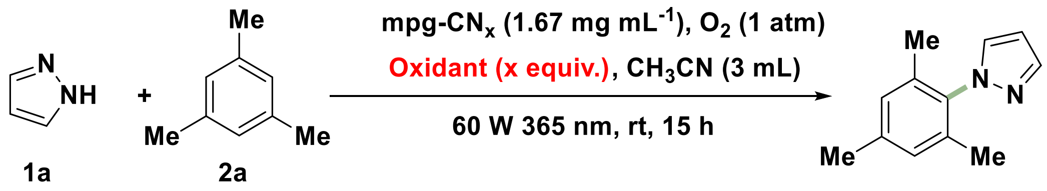

Entry	**2a** (equiv.)	Oxidant (x eq.)	Conversion (%)[a]	Yield (%)[a]
1	3	TBHP (3 eq.)	38	30
2	3	TEMPO (3 eq.)	49	7
3	3	H_2O_2 (3 eq.)	17	15
4	3	$PhI(OAc)_2$ (3 eq.)	72	24
5	3	BQ (3 eq.)	39	26
6	3	$Na_2S_2O_8$ (3 eq.)	58	50
7	3	$Na_2S_2O_8$ (1 eq.)	62	51
8	6	$Na_2S_2O_8$ (1 eq.)	71	61
9	6	$(NH_4)_2S_2O_8$ (1 eq.)	53	44
**10**	**6**	**$K_2S_2O_8$ (1 eq.)**	**94**	**82**
11	6	DDQ (1 eq.)	97	66
12	6	$K_2S_2O_8$ (0.5 eq.)	83	71
13	6	-	57	56

[a] Reaction conditions: pyrazole (0.2 mmol), mesitylene (0.6-1.2 mmol, 3-6 equiv.), O_2 (1 atm), oxidant (0.5-3 equiv.), mpg-CN$_x$ (5 mg, 1.67 mg mL^{-1}), CH_3CN (3 mL), 60 W 365 nm UV light at room temperature for 15 h. [b] The conversion of pyrazole was determined by ^{1}H-NMR using pyrazine as external standard. [c] The yield was determined by ^{1}H-NMR using pyrazine as external standard. Abbreviation: TBHP is tert-Butyl hydroperoxide; BQ is 1,4-Benzoquinone; DDQ is 2,3-Dichloro-5,6-dicyano-1,4-benzoquinone.

5.4 mpg-CN_x loading screening

Table S3. mpg-CN_x loading screening. [a]

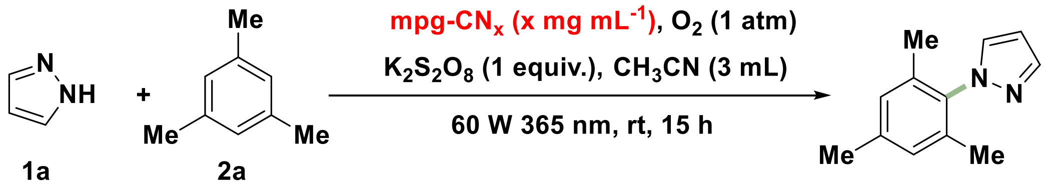

Entry	mpg-CN_x loading (mg mL^{-1})	Conversion (%)[b]	Yield (%)[c]
**1**	**1.67**	**94**	**82**
2	0.83	93	75
3	0.43	91	72
4	-	8	7
5[d]	-	22	13

[a] Reaction conditions: pyrazole (0.2 mmol), mesitylene (1.2 mmol, 6 equiv.), O_2 (1 atm), $K_2S_2O_8$ (1 equiv.), mpg-CN_x (x mg mL^{-1}), CH_3CN (3 mL), 60 W 365 nm UV light at room temperature for 15 h. [b] The conversion of pyrazole was determined by ^{1}H-NMR using pyrazine as external standard. [c] The yield was determined by ^{1}H-NMR using pyrazine as external standard. [d] Control experiment without mpg-CNx in flow conditions.

5.5 Mesitylene equivalent screening

Table S4. Mesitylene equivalent screening. [a]

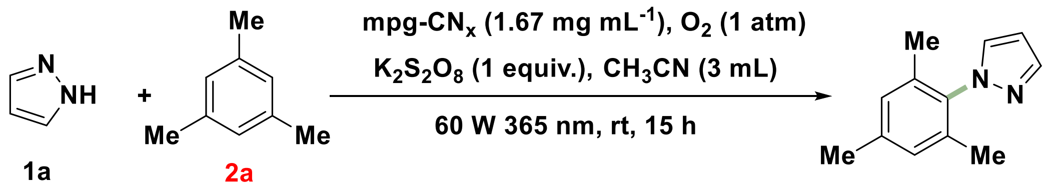

Entry	**2a** (equiv.)	Conversion (%)[b]	Yield (%)[c]
**1**	**6**	**94**	**82**
2	3	96	78
3	2	91	75
4	1.5	90	58

[a] Reaction conditions: pyrazole (0.2 mmol), mesitylene (x equiv.), O_2 (1 atm), $K_2S_2O_8$ (1 eq.), mpg-CN_x (1.67 mg mL^{-1}), CH_3CN (3 mL), 60 W 365 nm UV light at room temperature for 15 h. [b] The conversion of pyrazole was determined by ^{1}H-NMR using pyrazine as external standard. [c] The yield was determined by ^{1}H-NMR using pyrazine as external standard.

5.6 Light source screening

Table S5. Light source screening. [a]

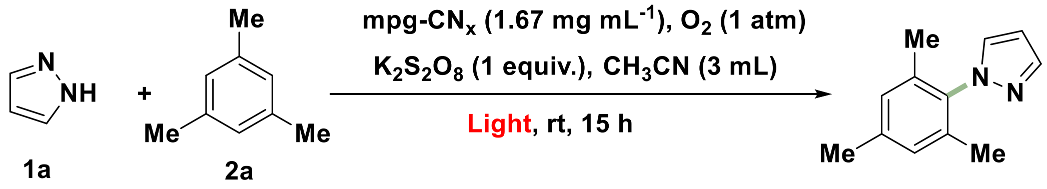

Entry	Light	Conversion (%)[b]	Yield (%)[c]
**1**	**60 W 365 nm**	**94**	**82**
2	36 W 365 nm	69	62
3	18 W blue light	71	60
4	26 W white light	77	61

[a] Reaction conditions: pyrazole (0.2 mmol), mesitylene (6 equiv.), O_2 (1 atm), $K_2S_2O_8$ (1 equiv.), mpg-CN_x (1.67 mg mL^{-1}), CH_3CN (3 mL), irradiated with different light at room temperature for 15 h. [b] The conversion of pyrazole was determined by ^{1}H-NMR using pyrazine as external standard. [c] The yield was determined by ^{1}H-NMR using pyrazine as external standard.

6. Light-Dependence Experiments

Following the **GP1**, the reaction between pyrazole (0.2 mmol, 13.6 mg) and mesitylene (6 equiv., 166.5 μL) was performed. Pyrazine was used as an internal standard (0.1 mmol, 0.5 equiv., 8 mg). Aliquots (100 μL) of the reaction mixture were taken during 10.5 h and analyzed by ^{1}H NMR, as shown in Figure S7. In Figure S8, the reaction vial was first wrapped with aluminium foil and kept in a black box for 150 min. During this time, no product formation was observed. After that, the aluminium foil was removed and the reaction continued under light irradiation, showing a similar plot as **Figure S7**. Similarly, when the irradiation was stopped from the 180th to the 240th min, the yield stopped increasing (**Figure S9**). When accounting for the total irradiation time (by substracting the dark periods in control experiments during the initial phase and the middle phase) the reaction profile of all experiments show a good overlap (**Figure S10**). This indicates that light irradiation is always required all over the reaction time.

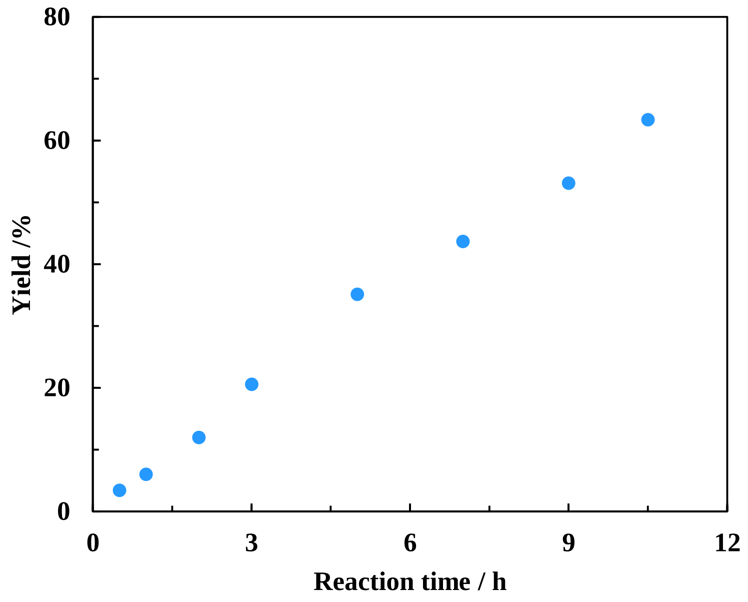

Figure S7. Kinetic profile of the reaction with continuous irradiation.

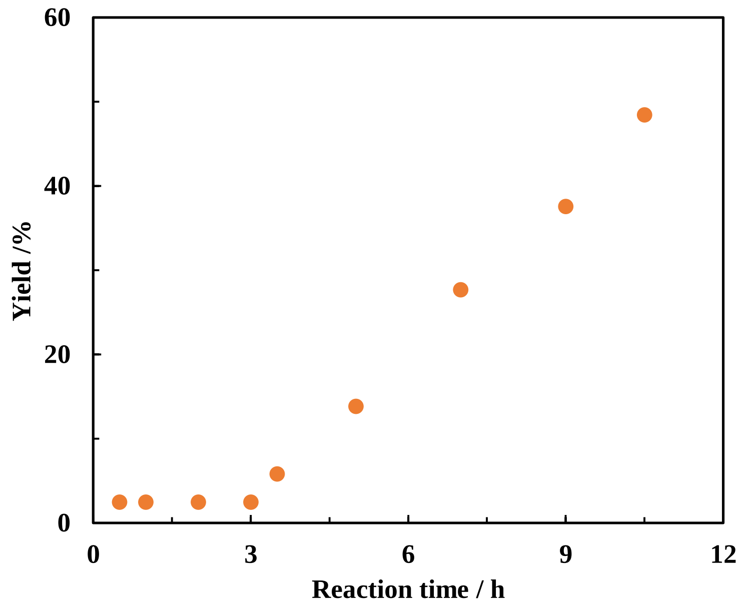

Figure S8. 150 min in the dark at the beginning (30th-180th min).

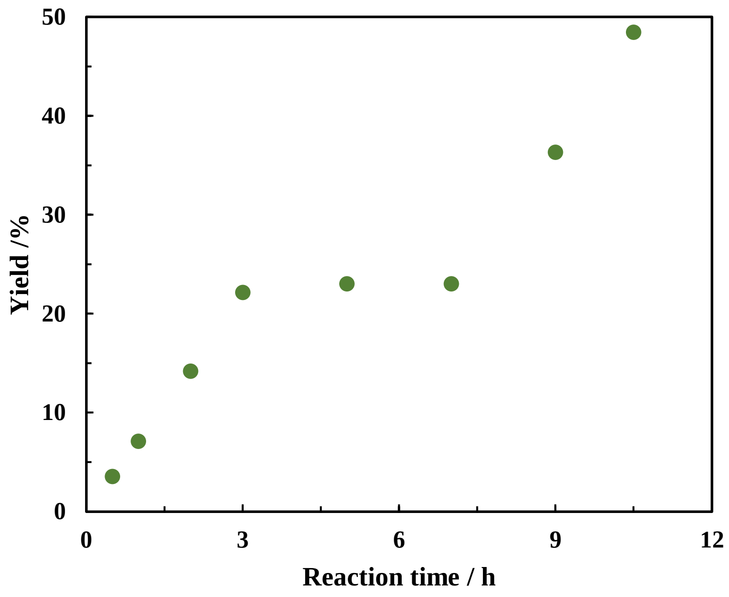

Figure S9. 60 min in the dark in the middle of the experiment (180th-240th min).

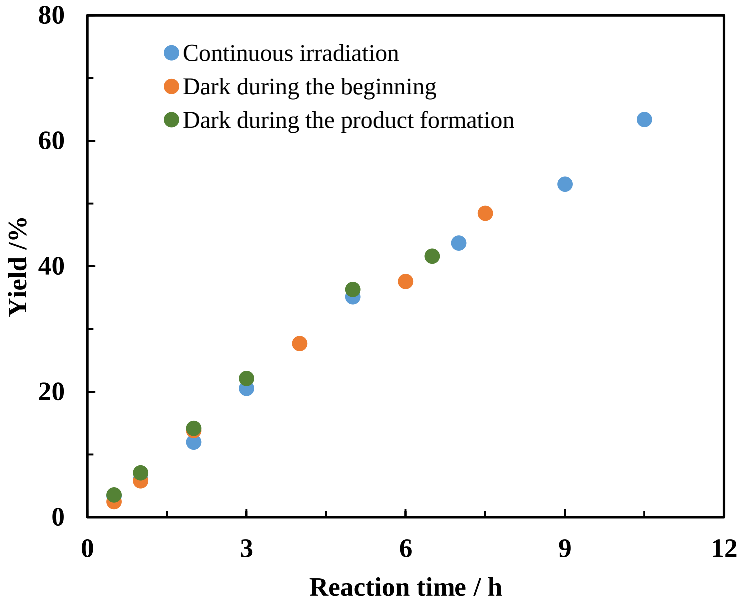

Figure S10. Overlap of figures S7-9 substracting the time without irradiation.

7. Reproducibility investigation with different batches of mpg-CNx

Heterogeneous catalysts are often plagued by poor reproducibility of the synthetic procedure, where some batches perform optimal and others much worse for a given transformation. To rule out inter-batch variability issues, we carried out three kinetic studies with three different catalyst batches (prepared on different days and on different scales).

Following the **GP1**, the reactions between pyrazole (0.2 mmol, 13.6 mg) and mesitylene (6 equiv., 166.5 μL) with three different batches of mpg-CNx were performed. Pyrazine was used as an internal standard (0.1 mmol, 0.5 equiv., 8 mg). Aliquots (100 μL) of the reaction mixture were taken during 25 h and analyzed by ^{1}H NMR, as shown in Figure S11.

The reaction profiles with three different batches of mpg-CNx show a similar kinetic performance (within experimental error), which indicate that mpg-CNx is a robust photocatalyst and can be reliably synthesized without noticeable inter-batch variability.

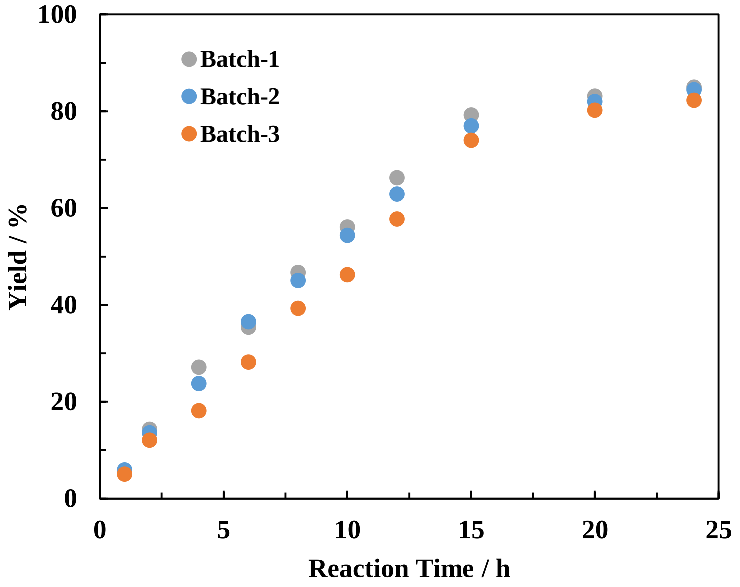

Figure S11. Stabilization test of three different batches of mpg-CNx.

8. Substrate preparation

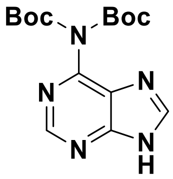

***Tert*-butyl (*tert*-butoxycarbonyl)(9*H*-purin-6-yl)carbamate (S1). S1** was synthesized according to a procedure reported in the literature.[3] To a 100 mL N_2-flushed round-bottomed flask equipped with a magnetic stirring bar, adenine (1.35 g, 10.0 mmol) and DMAP (0.1 equiv., 1.0 mmol, 0.122 g) were added to be then suspended in 50 mL of dry THF using a gas-tight syringe. To this suspension, 8.7 g (3.98 equiv., 39.8 mmol) of Boc_2O were added under N_2 atmosphere. The reaction mixture was stirred for 12 h at room temperature, after which the solvent was removed by rotary evaporation to give a yellow oil, which was diluted with ethyl acetate (400 mL). This solution was washed with 1 N HCl (30 mL) and brine (3 × 100 mL). The organic layer was dried over Na_2SO_4 and evaporated to give a colourless oil corresponding to tris-Boc-adenine, which was used in the next step without further purification.

The tris-Boc-adenine was first dissolved in CH_3OH (100 mL), and then saturated $NaHCO_3$ solution (45 mL) was added. The so-obtained turbid mixture was stirred at 50 ^oC for 1 h. The reaction progress was monitored via TLC (cyclohexane:ethyl acetate 7:3). After evaporating CH_3OH, water (100 mL) was added to the mixture and the aqueous layer was extracted with $CHCl_3$ (2 × 300 mL). The organic layer was dried over Na_2SO_4, filtered, and evaporated to give a white solid. The crude material was dissolved in ethyl acetate and purified through column chromatography (ethyl acetate) to give **S1** (6.3 mmol, 2.1 g, 63% yield) as a white solid. Spectroscopic data are in agreement with the literature.[3]

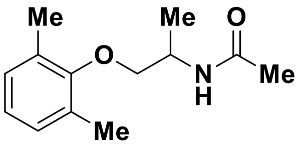

***N*-(1-(2,6-Dimethylphenoxy)propan-2-yl)acetamide (S2). S2** was synthesized according to a procedure reported in the literature.[4] Acetyl chloride (0.50 mL, 6.95 mmol, 1.5 equiv.) was slowly added to a stirred suspension of mexiletine hydrochloride (1.00 g, 4.64 mmol, 1.0 equiv.) in EtOAc/5 N NaOH (1:1, 10 mL) at 0 °C. After the addition, the reaction mixture was allowed to warm up to ambient temperature and to stir for 1 hour. Water (10 mL) was then added and the aqueous layer was extracted using EtOAc (3 × 15 mL). The combined organic layers were washed with a aqueous saturated $NaHCO_3$ solution (3 × 15 mL) and brine (15 mL), dried over $MgSO_4$, filtered, and concentrated in vacuo, to give **S2** (0.99 g, 4.50 mmol, 97%) as a white solid. The spectroscopic data are in agreement with the literature.[4]

9. Characterization data

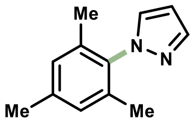

1-Mesityl-1*H*-pyrazole (1). The product was prepared following the general procedure **GP1** (15 h reaction time) and was purified by flash column chromatography on silica gel (Cyclohexane:Ethyl Acetate 98:2) to afford the product as a pale yellow solid (89.3 mg, 80 %). m.p. (55-57 °C). **^{1}H NMR** (400 MHz, $CDCl_3$) δ 7.73 (d, *J* = 1.8 Hz, 1H), 7.43 (d, *J* = 2.3 Hz, 1H), 6.94 (s, 2H), 6.44 (t, *J* = 2.1 Hz, 1H), 2.33 (s, 3H), 1.96 (s, 6H). **^{13}C NMR** (100 MHz, $CDCl_3$) δ 140.0, 138.9, 136.9, 136.0, 131.0, 128. 9, 105. 9, 21.2, 17.3. **HRMS (ESI)**: m/z Calc. for $C_{12}H_{15}N_2$ $[M + H]^+$ 187.1230; Found 187.1235. The spectroscopic data are in agreement with the literature.[5]

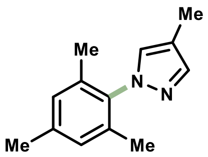

1-Mesityl-4-methyl-1*H*-pyrazole (2). The product was prepared following the general procedure **GP1** (15 h reaction time) and was purified by flash column chromatography on silica gel (Cyclohexane:Ethyl Acetate 98:2) to afford the product as a colourless oil (75.6 mg, 63 %). **^{1}H NMR** (400 MHz, $CDCl_3$) δ 7.53 (s, 1H), 7.19 (s, 1H), 6.92 (s, 2H), 2.32 (s, 3H), 2.17 (s, 3H), 1.97 (s, 6H). **^{13}C NMR** (100 MHz, $CDCl_3$) δ 140.4, 138.7, 137.1, 136.0, 129.8, 128.8, 116.3, 21.2, 17.4, 9.1. **HRMS (ESI)** m/z Calc. for $C_{13}H_{17}N_2$ $[M + H]^+$ 201.1386; Found 201.1392.
The spectroscopic data are in agreement with the literature.[6]

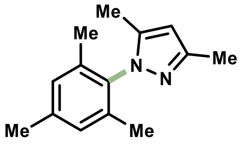

1-Mesityl-3,5-dimethyl-1*H*-pyrazole (3). The product was prepared following the general procedure **GP1** (24 h reaction time) and was purified by flash column chromatography on silica gel (Cyclohexane:Ethyl Acetate 98:2) to afford the product as a colourless oil (64.3 mg, 50 %). **^{1}H NMR** (400 MHz, $CDCl_3$) δ 6.91 (s, 2H), 5.95 (s, 1H), 2.31 (s, 3H), 2.28 (s, 3H), 1.96 (s, 3H), 1.92 (s, 6H). **^{13}C NMR** (100 MHz, $CDCl_3$) 148.6, 140.1, 138.6, 136.5, 135.4, 128.8, 104.5, 21.2, 17.3, 13.8, 11.0. **HRMS (ESI)** m/z Calc. for $C_{14}H_{19}N_2$ $[M + H]^+$ 215.1543; Found 215.1548.
The spectroscopic data are in agreement with the literature.[7]

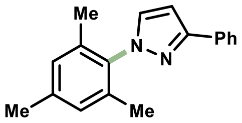

1-Mesityl-3-phenyl-1*H*-pyrazole (4). The product was prepared following the general procedure **GP1** (15 h reaction time) and was purified by flash column chromatography on silica gel (Cyclohexane:Ethyl Acetate 98:2) to afford the product as a yellow oil (99.1 mg, 63 %). **^{1}H**

NMR (400 MHz, $CDCl_3$) δ 7.92 – 7.87 (m, 2H), 7.46 (d, *J* = 2.3 Hz, 1H), 7.44 – 7.38 (m, 2H), 7.34 – 7.29 (m, 1H), 6.97 (s, 2H), 6.75 (d, *J* = 2.3 Hz, 1H), 2.34 (s, 3H), 2.05 (s, 6H). **^{13}C NMR** (100 MHz, $CDCl_3$) δ 152.1, 139.0, 137.1, 136.0, 133.6, 132.5, 129.0, 128.7, 127.8, 125.9, 103.2, 21.2, 17.5. **HRMS (ESI)** m/z Calc. for $C_{18}H_{19}N_2$ $[M + H]^+$ 263.1543; Found 263.1548.

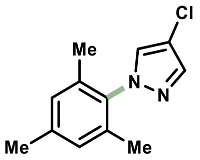

4-Chloro-1-mesityl-1*H*-pyrazole (5). The product was prepared following the general procedure **GP1** (15 h reaction time) and was purified by flash column chromatography on silica gel (Cyclohexane:Ethyl Acetate 98:2) to afford the product as a pale-yellow oil (107.0 mg, 81 %). **^{1}H NMR** (400 MHz, $CDCl_3$) δ 7.64 (s, 1H), 7.42 (s, 1H), 6.94 (s, 2H), 2.33 (s, 3H), 1.99 (s, 6H). **^{13}C NMR** (100 MHz, $CDCl_3$) δ 139.4, 138.6, 136.6, 135.8, 129.0, 128.9, 110.5, 21.2, 17.3. **HRMS (ESI)** m/z Calc. for $C_{12}H_{14}ClN_2$ $[M + H]^+$ 221.0840; Found 221.0846.
The spectroscopic data are in agreement with the literature.[8]

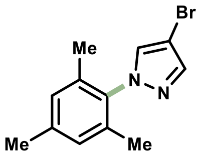

4-Bromo-1-mesityl-1*H*-pyrazole (6). The product was prepared following the general procedure **GP1** (15 h reaction time) and was purified by flash column chromatography on silica gel (Cyclohexane:Ethyl Acetate 98:2) to afford the product as a colourless oil (123.6 mg, 78 %). **^{1}H NMR** (400 MHz, $CDCl_3$) δ 7.68 (s, 1H), 7.45 (s, 1H), 6.94 (s, 2H), 2.33 (s, 3H), 1.98 (s, 6H). **^{13}C NMR** (100 MHz, $CDCl_3$) δ 140.7, 139.4, 136.5, 135.8, 131.1, 129.0, 93.6, 21.2, 17.3. **HRMS (ESI)** m/z Calc. for $C_{12}H_{14}BrN_2$ $[M + H]^+$ 265.0335; Found 265.0340.
The spectroscopic data are in agreement with the literature.[8b]

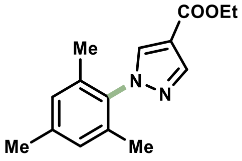

Ethyl 1-mesityl-1*H*-pyrazole-4-carboxylate (7). The product was prepared following the general procedure **GP1** (15 h reaction time) and was purified by flash column chromatography on silica gel (Cyclohexane:Ethyl Acetate 99:1) to afford the product as a yellow oil (106.8 mg, 69 %). **^{1}H NMR** (400 MHz, $CDCl_3$) δ 8.12 (s, 1H), 7.93 (s, 1H), 6.95 (s, 2H), 4.33 (q, *J* = 7.1 Hz, 2H), 2.33 (s, 3H), 1.98 (s, 6H), 1.37 (t, *J* = 7.1 Hz, 3H). **^{13}C NMR** (100 MHz, $CDCl_3$) δ 163.3, 141.7, 139.6, 136.2, 135.5, 134.6, 129.1, 115.7, 60.4, 21.2, 17.3, 14.5. **HRMS (ESI)** m/z Calc. for $C_{15}H_{19}N_2O_2$ $[M + H]^+$ 259.1441; Found 259.1447.
The spectroscopic data are in agreement with the literature.[8b]

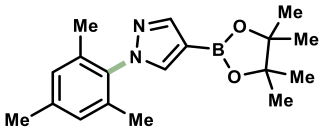

1-Mesityl-4-(4,4,5,5-tetramethyl-1,3,2-dioxaborolan-2-yl)-1*H*-pyrazole (8). The product was prepared following the general procedure **GP1** (15 h reaction time) and was purified by flash column chromatography on silica gel (Cyclohexane:Ethyl Acetate 95:5) to afford the product as a colourless oil (71.1 mg, 38 %). **^{1}H NMR** (400 MHz, $CDCl_3$) δ 7.99 (s, 1H), 7.70 (s, 1H), 6.93 (s, 2H), 2.32 (s, 3H), 1.96 (s, 6H), 1.35 (s, 12H). **^{13}C NMR** (100 MHz, $CDCl_3$) δ 146.1, 138.9, 138.0, 136.6, 135.7, 128.9, 83.5, 25.0, 21.2, 17.4. **HRMS (ESI)** m/z Calc. for $C_{18}H_{26}BN_2O_2$ [M + H]$^+$ 313.2082; Found 313.2091.

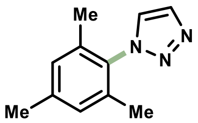

1-Mesityl-1*H*-1,2,3-triazole (9). The product was prepared following the general procedure **GP1** (24 h reaction time) and was purified by flash column chromatography on silica gel (Cyclohexane:Ethyl Acetate 97:3) to afford the product as a a white solid (92.1 mg, 82 %). m.p. 177-179 °C. **^{1}H NMR** (400 MHz, $CDCl_3$) δ 7.89 (d, *J* =0.9 Hz 1H), 7.62 (d, *J* = 1.0 Hz, 1H), 7.00 (s, 2H), 2.36 (s, 3H), 1.95 (s, 6H). **^{13}C NMR** (100 MHz, $CDCl_3$) δ 140.1, 135.3, 133.8, 133.6, 129.2, 125.5, 21.3, 17.4. **HRMS (ESI)** m/z Calc. for $C_{11}H_{14}N_3$ [M + H]$^+$ 188.1182; Found 188.1177.
The spectroscopic data are in agreement with the literature.[8b]

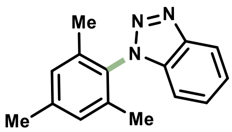

1-Mesityl-1*H*-benzo[d][1,2,3]triazole (10). The product was prepared following the general procedure **GP1** (24 h reaction time) and was purified by flash column chromatography on silica gel (Cyclohexane:Ethyl Acetate 99:1) to afford the product as a colourless oil (135.2 mg, 95 %). **^{1}H NMR** (400 MHz, $CDCl_3$) δ 8.12 (d, *J* = 8.3 Hz, 1H), 7.46 – 7.41 (m, 1H), 7.40 – 7.35 (m, 1H), 7.17 (d, *J* = 8.1 Hz, 1H), 7.03 (s, 2H), 2.36 (s, 3H), 1.83 (s, 6H). **^{13}C NMR** (100 MHz, $CDCl_3$) δ 145.4, 140.2, 136.1, 133.8, 131.6, 129.3, 128.0, 124.0, 120.0, 109.7, 21.2, 17.3. **HRMS (ESI)** m/z Calc. for $C_{15}H_{16}N_3$ [M + H]$^+$ 238.1339; Found 238.1344.
The spectroscopic data are in agreement with the literature.[9]

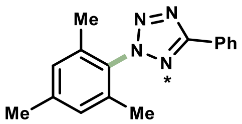

2-Mesityl-5-phenyl-2*H*-tetrazole (11) and 1-mesityl-5-phenyl-1*H*-tetrazole (11'). The compounds were prepared following the general procedure **GP1** (24 h reaction time). Product **11** was purified by flash column chromatography on silica gel (Cyclohexane:Ethyl Acetate 98:2) to afford the product as a white solid (45.9 mg, 29 %). m.p. 91-93 °C. **^{1}H NMR** (400 MHz, $CDCl_3$) 8.26 (dd, *J* = 7.4, 1.9 Hz, 2H), 7.52 (d, *J* = 7.1 Hz, 3H), 7.04 (s, 2H), 2.39 (s, 3H), 2.02 (s, 6H). **^{13}C NMR** (100

MHz, $CDCl_3$) δ 165.2, 141.0, 135.2, 134.0, 130.6, 129.3, 129.1, 127.5, 127.1, 21.4, 17.5. **HRMS (ESI)** m/z Calc. for $C_{16}H_{17}N_4$ $[M + H]^+$ 265.1448; Found 246.1453.

Product **11'** was purified by flash column chromatography on silica gel (Cyclohexane: Ethyl Acetate 98:2) to afford the product as a pale yellow oil (12.7 mg, 8 %). **^{1}H NMR** (400 MHz, $CDCl_3$) 7.61 – 7.57 (m, 2H), 7.49 – 7.43 (m, 1H), 7.39 – 7.34 (m, 2H), 7.04 (s, 2H), 2.39 (s, 3H), 1.88 (s, 6H). **^{13}C NMR** (100 MHz, $CDCl_3$) δ 154.0, 141.2, 135.3, 131.5, 130.9, 130.0, 129.3, 127.7, 123.8, 21.4, 17.6. **HRMS (ESI)** m/z Calc. for $C_{16}H_{17}N_4$ $[M + H]^+$ 265.1448; Found 246.1453.

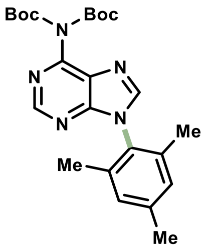

Tert-butyl (tert-butoxycarbonyl)(9-mesityl-9*H*-purin-6-yl)carbamate (12). The product was prepared following the general procedure **GP1** (24 h reaction time) and was purified by flash column chromatography on silica gel (Cyclohexane:Ethyl Acetate 90:10) to afford the product as a colourless oil (81.6 mg, 30 %). **^{1}H NMR** (400 MHz, $CDCl_3$) δ 8.82 (s, 1H), 8.03 (s, 1H), 7.04 (s, 2H), 2.35 (s, 3H), 1.91 (s, 6H), 1.44 (s, 18H). **^{13}C NMR** (100 MHz, $CDCl_3$) 153.6, 152.9, 150.8, 150.3, 145.1, 140.3, 135.8, 129.7, 129.1, 128.4, 83.8, 27.8, 21.2, 17.7. **HRMS (ESI)** m/z Calc. for $C_{24}H_{32}N_5O_4$ $[M + H]^+$ 454.2449; Found 454.2454.

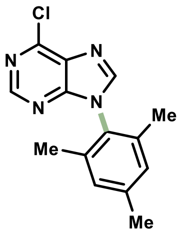

6-Chloro-9-mesityl-9*H*-purine (13). The product was prepared following the general procedure **GP1** (24 h reaction time) and was purified by flash column chromatography on silica gel (Cyclohexane: Ethyl Acetate 91:9) to afford the product as a white solid (63.7 mg, 39 %). m.p. 143-145 °C. **^{1}H NMR** (400 MHz, $CDCl_3$) δ 8.72 (s, 1H), 8.11 (s, 1H), 7.05 (s, 2H), 2.36 (s, 3H), 1.93 (s, 6H). **^{13}C NMR** (100 MHz, $CDCl_3$) δ 152.8, 152.1, 151.7, 145.8, 140.6, 135.7, 131.3, 129.8, 128.9, 21.2, 17.8. **HRMS (ESI)** m/z Calc. for $C_{14}H_{14}ClN_4$ $[M + H]^+$ 273.0902; Found 273.0907.

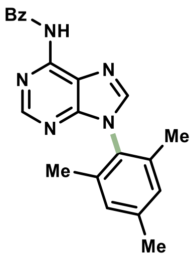

***N*-(9-mesityl-9*H*-purin-6-yl) benzamide (14).** The product was prepared following the general procedure **GP1** (24 h reaction time) and was purified by flash column chromatography on silica gel (Cyclohexane:Ethyl Acetate 40:60) to afford the product as a white solid (47.1 mg, 22 %). m.p. 203-205 °C. **^{1}H NMR** (400 MHz, $CDCl_3$) δ 9.34 (brs, 1H), 8.74 (s, 1H), 8.06 (d, *J* = 7.4 Hz, 2H), 7.98 (s, 1H), 7.62 – 7.57 (m, 1H), 7.54 – 7.48 (m, 2H), 7.05 (s, 2H), 2.36 (s, 3H), 1.95 (s, 6H). **^{13}C NMR** (100 MHz, $CDCl_3$) δ 164.9, 153.4, 152.5, 150.0, 143.5, 140.3, 135.9, 133.8,

132.9, 129.7, 129.2, 128.9, 128.0, 122.8, 21.2, 17.8. **HRMS (ESI)** m/z Calc. for $C_{21}H_{20}N_4O$ $[M + H]^+$ 358.1662; Found 358.1668.

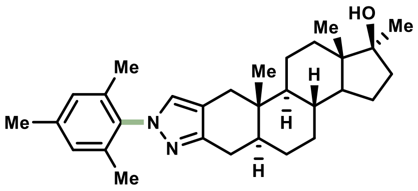

(1S,3bR,5aS,10aS,10bS,12aS)-8-mesityl-1,10a,12a-trimethyl-1,2,3,3a,3b,4,5,5a,6,8,10,10a,10b,11,12,12a-hexadecahydrocyclopenta[5,6] naphtho[1,2-f]indazol-1-ol (15). The product was prepared following the general procedure **GP1** (40 h reaction time) and was purified by flash column chromatography on silica gel (Cyclohexane:Ethyl Acetate 80:20) to afford the product as a yellow solid (80.3 mg, 30 %). m.p. 188-190 °C. **^{1}H NMR** (400 MHz, $CDCl_3$) δ 7.06 (s, 1H), 6.90 (s, 2H), 2.69 (dd, *J* = 16.0, 5.7 Hz, 2H), 2.41 – 2.32 (m, 1H), 2.30 (s, 3H), 2.22 – 2.16 (m, 1H), 1.97 (s, 6H), 1.83 – 1.73 (m, 3H), 1.68 – 1.59 (m, 4H), 1.56 – 1.51 (m, 1H), 1.49 – 1.38 (m, 2H), 1.37 – 1.21 (m, 7H), 1.00 – 0.84 (m, 5H), 0.82 (s, 3H). **^{13}C NMR** (100 MHz, $CDCl_3$) δ 148.5, 138.3, 137.5, 136.1, 128.7, 128.5, 115.5, 81.9, 54.1, 50.8, 45.5, 42.8, 39.1, 36.9, 36.6, 35.1, 31.8, 31.7, 29.6, 27.8, 25.9, 23.5, 21.2, 21.0, 17.4, 14.0, 11.7. **HRMS (ESI)** m/z Calc. for $C_{30}H_{43}N_2O$ $[M + H]^+$ 447.3370; Found 447.3375.

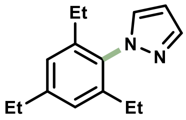

1-(2,4,6-Triethylphenyl)-1*H*-pyrazole (16). The product was prepared following the general procedure **GP1** (15 h reaction time) and was purified by flash column chromatography on silica gel (Cyclohexane:Ethyl Acetate 99:1) to afford the product as a colourless oil (117.7 mg, 86 %). **^{1}H NMR** (400 MHz, $CDCl_3$) δ 7.71 (d, *J* = 1.4 Hz, 1H), 7.45 (d, *J* = 1.9 Hz, 1H), 7.00 (s, 2H), 6.42 (t, *J* = 2.1 Hz, 1H), 2.67 (q, *J* = 7.6 Hz, 2H), 2.31 – 2.17 (m, 4H), 1.27 (t, *J* = 7.6 Hz, 3H), 1.07 (t, *J* = 7.6 Hz, 6H). **^{13}C NMR** (100 MHz, $CDCl_3$) δ 145.6, 142.1, 139.8, 136.1, 131.7, 126.1, 105.7, 28.9, 24.4, 15.7, 15.5. **HRMS (ESI)** m/z Calc. for $C_{15}H_{21}N_2$ $[M + H]^+$ 229.1699; Found 229.1694.
The spectroscopic data are in agreement with the literature.[10]

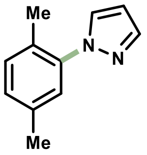

1-(2,5-Dimethylphenyl)-1*H*-pyrazole (17). The product was prepared following the general procedure **GP1** (15 h reaction time) and was purified by flash column chromatography on silica gel (Cyclohexane:Ethyl Acetate 98:2) to afford the product as a colourless oil (36.1 mg, 35 %). **^{1}H NMR** (400 MHz, $CDCl_3$) δ 7.71 (d, *J* = 1.9 Hz, 1H), 7.59 (d, *J* = 2.4 Hz, 1H), 7.21 – 7.15 (m, 2H), 7.12 (dd, *J* = 7.8, 1.8 Hz, 1H), 6.43 (t, *J* = 2.1 Hz, 1H), 2.35 (s, 3H), 2.20 (s, 3H). **^{13}C NMR** (100 MHz, $CDCl_3$) δ 140.2, 139.8, 136.5,

131.1, 130.5, 130.3, 129.1, 126.8, 106.1, 20.8, 17.7. **HRMS (ESI)** m/z Calc. for $C_{11}H_{13}N_2$ [M + H]$^+$ 173.1073; Found 173.1079.
The spectroscopic data are in agreement with the literature.[10]

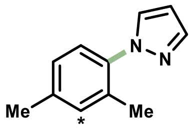

1-(2,4-Dimethylphenyl)-1*H*-pyrazole (18) and 1-(2,6-dimethylphenyl)-1*H*-pyrazole (18’). The product was prepared following the general procedure **GP1** (15 h reaction time) and was purified by flash column chromatography on silica gel (Cyclohexane:Ethyl Acetate 99:1) to afford the product as a colourless oil (97.1 mg, 94 %). The major (**18**): minor (**18’**) ratio of the inseparable mixture was 11:1 as determined by ^{1}H NMR of the isolated product.
^{1}H NMR (400 MHz, $CDCl_3$) δ 7.74 (d, *J* = 1.6 Hz, $1H_{minor}$), 7.70 (d, *J* = 1.9 Hz, $1H_{major}$), 7.56 (d, *J* = 2.0 Hz, $1H_{major}$), 7.45 (d, *J* = 2.0 Hz, $1H_{minor}$), 7.25 – 7.22 (m, $1H_{minor}$), 7.20 (d, *J* = 7.9 Hz, $1H_{major}$), 7.14 (s, $1H_{minor}$), 7.13 – 7.11(m, $1H_{major}$), 7.09 – 7.05 (m, 2H), 6.45 (t, *J* = 2.1 Hz, $1H_{minor}$), 6.42 (t, *J* = 2.1 Hz, $1H_{major}$), 2.37 (s, $3H_{major}$), 2.19 (s, $3H_{major}$) 2.01 (s, $6H_{minor}$). **^{13}C NMR** (100 MHz, $CDCl_3$) δ 140.2, 140.1, 138.3, 137.7, 136.3, 133.5, 131.9, 130.8, 130.6, 129.0, 128.2, 127.2, 126.1, 106.1, 105.9, 21.1, 18.0, 17.3. **HRMS (ESI)** m/z Calc. for $C_{11}H_{13}N_2$ [M + H]$^+$ 173.1073; Found 173.1068.
The spectroscopic data are in agreement with the literature.[10]

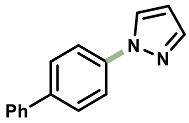

1-([1,1'-Biphenyl]-4-yl)-1*H*-pyrazole (19). The product was prepared following the general procedure GP1 (24 h reaction time) and was purified by flash column chromatography on silica gel (Cyclohexane:Ethyl Acetate 95:5) to afford the product as a white solid (58.1 mg, 44 %). m.p. 129-131 °C. **^{1}H NMR** (400 MHz, $CDCl_3$) δ 7.97 (d, *J* = 2.5 Hz, 1H), 7.82 – 7.74 (m, 3H), 7.72 – 7.67 (m, 2H), 7.65 – 7.60 (m, 2H), 7.50 – 7.44 (m, 2H), 7.41 – 7.34 (m, 1H), 6.50 (t, *J* = 2.1 Hz, 1H). **^{13}C NMR** (100 MHz, $CDCl_3$) δ 141.3, 140.2, 139.5, 139.4, 129.0, 128.1, 127.6, 127.1, 126.8, 119.5, 107.8. **HRMS (ESI)** m/z Calc. for $C_{15}H_{13}N_2$ [M + H]$^+$ 221.1073; Found 221.1076.
The spectroscopic data are in agreement with the literature.[10]

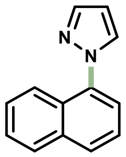

1-(Naphthalen-1-yl)-1*H*-pyrazole (20). The product was prepared following the general procedure **GP1** (15 h reaction time) and was purified by flash column chromatography on silica gel (Cyclohexane:Ethyl Acetate 96:4) to afford the product as a yellow oil (64.0 mg, 55 %). **^{1}H NMR** (400 MHz, $CDCl_3$) δ 7.95 – 7.90 (m, 2H), 7.87 (d, *J* = 1.9 Hz, 1H), 7.86 – 7.83 (m, 1H), 7.80 (d, *J* = 2.3 Hz, 1H), 7.58 – 7.49 (m, 4H),

6.55 (t, *J* = 2.1 Hz, 1H). **^{13}C NMR** (100 MHz, $CDCl_3$) δ 141.0, 137.5, 134.5, 131.8, 129.3, 129.1, 128.2, 127.3, 126.8, 125.2, 123.4, 123.3, 106.6. **HRMS (ESI)** m/z Calc. for $C_{13}H_{11}N_2$ $[M + H]^+$ 195.0917; Found 195.0922.

The spectroscopic data are in agreement with the literature.[11]

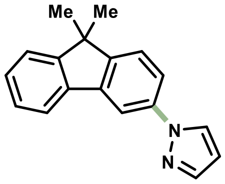

1-(9,9-Dimethyl-9*H*-fluoren-3-yl)-1*H*-pyrazole (21). The product was prepared following the general procedure **GP1** (24 h reaction time) and was purified by flash column chromatography on silica gel (Cyclohexane: Ethyl Acetate 70:30) to afford the product as a yellow oil (39.0 mg, 25 %). **^{1}H NMR** (400 MHz, $CDCl_3$) δ 7.99 (d, *J* = 2.8 Hz, 1H), 7.84 (d, *J* = 1.9 Hz, 1H), 7.77 (dd, *J* = 4.8, 3.3 Hz, 2H), 7.73 (dd, *J* = 6.2, 1.8 Hz, 1H), 7.62 (dd, *J* = 8.2, 2.1 Hz, 1H), 7.46 (dd, *J* = 6.9, 1.2 Hz, 1H), 7.39 – 7.31 (m, 2H), 6.50 (m, 1H), 1.55 (s, 6H). **^{13}C NMR** (100 MHz, $CDCl_3$) δ 155.3, 153.9, 141.1, 139.6, 138.4, 137.8, 127.5, 127.3, 127.1, 122.8, 120.8, 120.1, 118.2, 114.4, 107.7, 47.3, 27.2, 27.0. **HRMS (ESI)** m/z Calc. for $C_{18}H_{17}N_2$ $[M + H]^+$ 261.1386; Found 261.1392.

The spectroscopic data are in agreement with the literature.[10]

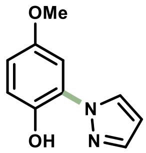

4-Methoxy-3-(1*H*-pyrazol-1-yl)phenol (22). The product was prepared following the general procedure **GP1** (15 h reaction time) and was purified by flash column chromatography on silica gel (Cyclohexane: Ethyl Acetate 97:3) to afford the product as a colourless oil (86.7 mg, 76 %). **^{1}H NMR** (400 MHz, $CDCl_3$) δ 7.95 (d, *J* = 2.5 Hz, 1H), 7.71 (d, *J* = 1.8 Hz, 1H), 7.02 (d, *J* = 8.9 Hz, 1H), 6.91 (d, *J* = 2.9 Hz, 1H), 6.75 (dd, *J* = 8.9, 2.9 Hz, 1H), 6.47 (t, *J* = 2.2 Hz, 1H), 3.78 (s, 3H). **^{13}C NMR** (100 MHz, $CDCl_3$) δ 152.7, 143.2, 139.1, 126.9, 124.8, 119.3, 112.8, 106.9, 104.4, 56.0. **HRMS (ESI)** m/z Calc. for $C_{10}H_{11}N_2O_2$ $[M + H]^+$ 191.0815; Found 191.0821.

The spectroscopic data are in agreement with the literature.[12]

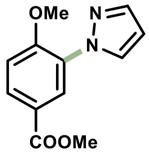

Methyl 4-methoxy-3-(1*H*-pyrazol-1-yl)benzoate (23). The product was prepared following the general procedure **GP1** (15 h reaction time) and was purified by flash column chromatography on silica gel (Cyclohexane: Ethyl Acetate 90:10) to afford the product as a pale-yellow oil (65.4 mg, 47 %). **^{1}H NMR** (400 MHz, $CDCl_3$) δ 8.39 (d, *J* = 2.2 Hz, 1H), 8.02

– 7.98 (m, 2H), 7.72 (d, *J* = 1.8, 0.6 Hz, 1H), 7.06 (d, *J* = 8.7 Hz, 1H), 6.43 (t, *J* = 2.5, 1.8 Hz, 1H), 3.93 (s, 3H), 3.88 (s, 3H). **^{13}C NMR** (100 MHz, $CDCl_3$) δ 166.2, 154.9, 140.5, 131.6, 130.0, 129.5, 126.8, 123.3, 111.8, 106.6, 56.3, 52.1. **HRMS (ESI)** m/z Calc. for $C_{12}H_{13}N_2O_3$ $[M + H]^+$ 233.0921; Found 233.0926.
The spectroscopic data are in agreement with the literature.[7]

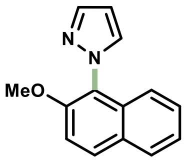

1-(2-Methoxynaphthalen-1-yl)-1*H*-pyrazole (24). The product was prepared following the general procedure **GP1** (24 h reaction time) and was purified by flash column chromatography on silica gel (Cyclohexane:Ethyl Acetate 93:7) to afford the product as a yellow oil (65.4 mg, 55 %). **^{1}H NMR** (400 MHz, $CDCl_3$) δ 7.85 (d, *J* = 9.1 Hz, 1H), 7.80 (d, *J* = 1.8 Hz, 1H), 7.74 (d, *J* = 7.6 Hz, 1H), 7.58 (d, *J* = 2.3 Hz, 1H), 7.37 – 7.32 (m, 1H), 7.32 – 7.25 (m, 2H), 7.18 (d, *J* = 8.3 Hz, 1H), 6.47 (t, *J* = 2.1 Hz, 1H), 3.78 (s, 3H). **^{13}C NMR** (100 MHz, $CDCl_3$) δ 152.6, 140.5, 133.0, 132.2, 130.7, 128.8, 127.8, 127.7, 124.4, 123.1, 122.1, 113.6, 106.0, 56.8. **HRMS (ESI)** m/z Calc. for $C_{14}H_{13}N_2O$ $[M + H]^+$ 225.1022; Found 225.1028.
The spectroscopic data are in agreement with the literature.[13]

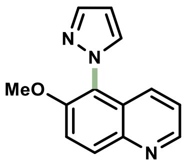

6-Methoxy-5-(1*H*-pyrazol-1-yl)quinoline (25). The product was prepared following the general procedure **GP1** (40 h reaction time) and was purified by flash column chromatography on silica gel (Cyclohexane:Ethyl Acetate 50:50) to afford the product as a yellow oil (35.2 mg, 26 %). **^{1}H NMR** (400 MHz, $CDCl_3$) δ 8.80 (dd, *J* = 4.2, 1.7 Hz, 1H), 8.22 (d, *J* = 9.4 Hz, 1H), 7.87 – 7.84 (m, 1H), 7.72 – 7.67 (m, 2H), 7.59 (d, *J* = 9.4 Hz, 1H), 7.34 (dd, *J* = 8.6, 4.1 Hz, 1H), 6.55 (t, *J* = 2.1 Hz, 1H), 3.91 (s, 3H). **^{13}C NMR** (100 MHz, $CDCl_3$) δ 152.5, 149.0, 143.4, 140.9, 133.2, 132.1, 131.1, 127.4, 122.6, 122.4, 116.7, 106.4, 57.0. **HRMS (ESI)** m/z Calc. for $C_{13}H_{12}N_3O$ $[M + H]^+$ 226.0975; Found 226.0974.
The spectroscopic data are in agreement with the literature.[5]

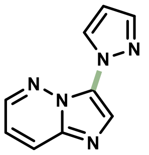

3-(1*H*-pyrazol-1-yl)imidazo[1,2-b]pyridazine (26). The product was prepared following the general procedure **GP1** (24 h reaction time) and was purified by flash column chromatography on silica gel (Cyclohexane:Ethyl Acetate 70:30) to afford the product as a brown solid (40.0 mg, 36 %). m.p. 86-88 °C. **^{1}H NMR** (400 MHz, DMSO-d_6) **^{1}H NMR** (400 MHz, DMSO-d_6) δ 8.61 (dd, *J* = 4.4, 1.5 Hz, 1H), 8.33 (d, *J* = 2.5 Hz, 1H), 8.25 (dd, *J* = 9.3, 1.6 Hz, 1H), 8.05 (s, 1H), 7.86 (d, *J* = 1.8 Hz, 1H), 7.35 (dd, *J* = 9.3,

4.4 Hz, 1H), 6.62 (t, *J* = 2.2 Hz, 1H).**^{13}C NMR** (100 MHz, DMSO-d$_6$) δ 144.6, 141.7, 136.9, 132.7, 127.6, 126.4, 126.2, 118.2, 107.3. **HRMS (ESI)** m/z Calc. for $C_9H_8N_5$ [M + H]$^+$ 186.0774; Found 186.0780.

The spectroscopic data are in agreement with the literature.[14]

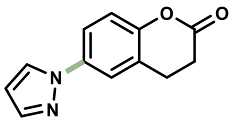

6-(1*H*-pyrazol-1-yl)chroman-2-one (27). The product was prepared following the general procedure **GP1** (40 h reaction time) and was purified by flash column chromatography on silica gel (Cyclohexane: Ethyl Acetate 81:19) to afford the product as a yellow oil (32.1 mg, 25 %). **^{1}H NMR** (400 MHz, $CDCl_3$) δ 7.87 (d, *J* = 2.3 Hz, 1H), 7.71 (d, *J* = 1.5 Hz, 1H), 7.61 (d, *J* = 2.5 Hz, 1H), 7.51 (dd, *J* = 8.7, 2.6 Hz, 1H), 7.12 (d, *J* = 8.7 Hz, 1H), 6.47 (m, 1H), 3.10 – 3.03 (m, 2H), 2.85 – 2.78 (t, *J* = 7.2 Hz, 2H). **^{13}C NMR** (100 MHz, $CDCl_3$) δ 168.1, 150.4, 141.3, 136.8, 126.9, 124.0, 119.3, 119.0, 117.9, 107.9, 29.1, 24.0. **HRMS (ESI)** m/z Calc. for $C_{12}H_{11}N_2O_2$ [M + H]$^+$ 215.0815; Found 215.0821.

The spectroscopic data are in agreement with the literature.[10]

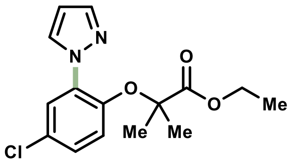

Ethyl 2-(4-chloro-2-(1*H*-pyrazol-1-yl)phenoxy)-2-methyl-propanoate (28). The product was prepared following the general procedure **GP1** (40 h reaction time) and was purified by flash column chromatography on silica gel (Cyclohexane:Ethyl Acetate 95:5) to afford the product as a colourless oil (20.3 mg, 11 %). **^{1}H NMR** (400 MHz, $CDCl_3$) δ 8.13 (d, *J* = 2.5Hz, 1H), 7.79 (d, *J* = 2.6 Hz, 1H), 7.69 (d, *J* = 1.8 Hz, 1H), 7.15 (dd, *J* = 8.8, 2.6 Hz, 1H), 6.95 (d, *J* = 8.8 Hz, 1H), 6.42 (t, *J* = 2.5 Hz, 1H), 4.23 (q, *J* = 7.1 Hz, 2H), 1.43 (s, 6H), 1.26 (t, *J* = 7.1 Hz, 3H). **^{13}C NMR** (100 MHz, $CDCl_3$) δ 173.5, 145.6, 140.7, 134.2, 132.0, 128.7, 127.2, 125.5, 121.6, 106.8, 81.7, 61.8, 25.0, 14.2. **HRMS (ESI)** m/z Calc. for $C_{15}H_{17}ClN_2O_3$ [M + H]$^+$ 309.1000; Found 309.1006.

The spectroscopic data are in agreement with the literature.[15]

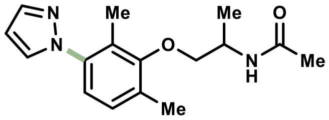

***N*-(1-(2,6-dimethyl-3-(1*H*-pyrazol-1-yl)phenoxy)propan-2-yl)acetamide (29).** The product was prepared following the general procedure **GP1** (40 h reaction time) and was purified by flash column chromatography on silica gel (Cyclohexane: Ethyl Acetate 80:20) to afford the product as a colourless oil (37.9 mg, 22 %). **^{1}H NMR** (400 MHz, $CDCl_3$) δ 7.71 (d, *J* = 2.2 Hz, 1H), 7.55 (d, *J* = 2.1 Hz, 1H), 7.09 (d, *J* = 8.1 Hz, 1H), 7.03 (d, *J* = 8.0 Hz, 1H), 6.43 (t, *J* = 2.1

Hz, 1H), 5.84 (brs,, 1H), 4.46 – 4.30 (m, 1H), 3.84 (dd, J = 9.1, 4.1 Hz, 1H), 3.76 (dd, J = 9.1, 3.3 Hz, 1H), 2.32 (s, 3H), 2.09 (s, 3H), 2.02 (s, 3H), 1.41 (d, J = 6.9 Hz, 3H). **^{13}C NMR** (100 MHz, $CDCl_3$) δ 169.6, 155.6, 140.3, 139.3, 131.6, 130.9, 128.7, 127.8, 122.3, 106.3, 74.4, 45.5, 23.6, 17.9, 16.4, 11.6. **HRMS (ESI)** m/z Calc. for $C_{16}H_{22}N_3O_2$ $[M + H]^+$ 288.1707; Found 288.1712.

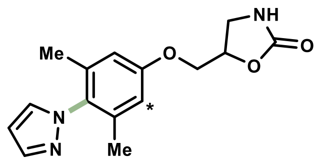

5-((3,5-Dimethyl-4-(1*H*-pyrazol-1-yl)phenoxy)methyl)oxazolidin-2-one (30) and 5-((3,5-dimethyl-2-(1*H*-pyrazol-1-yl)phenoxy)methyl)oxazolidin-2-one (30')

The molecule was prepared following the general procedure **GP1** (15 h reaction time). The product **30** was purified by flash column chromatography on silica gel (Cyclohexane:Ethyl Acetate 75:25) to afford the product as a white solid (88.4 mg, 51 %). m.p. 185-187 °C. **^{1}H NMR** (400 MHz, $CDCl_3$) δ 7.71 (d, J = 1.9 Hz, 1H), 7.41 (d, J = 2.3 Hz, 1H), 6.67 (s, 2H), 6.43 (t, J = 2.1 Hz, 1H), 5.97 (brs, 1H), 5.01 – 4.93 (m, 1H), 4.15 (d, J = 4.8 Hz, 2H), 3.77 (t, J = 8.8 Hz, 1H), 3.62 – 3.57 (m, 1H), 1.96 (s, 6H). **^{13}C NMR** (100 MHz, $CDCl_3$) δ 159.5, 158.0, 140.2, 138.0, 133.5, 131.2, 113.9, 106.0, 74.2, 68.2, 42.8, 17.7. **HRMS (ESI)** m/z Calc. for $C_{15}H_{18}N_3O_3$ $[M + H]^+$ 228.1343; Found 228.1348.

The product **30'** was purified by flash column chromatography on silica gel (Cyclohexane: Ethyl Acetate 25:75) to afford the product as a white solid (63.2 mg, 37 %). m.p. 138-140 °C. **^{1}H NMR** (400 MHz, $CDCl_3$) δ 7.70 (d, J = 1.8 Hz, 1H), 7.48 (d, J = 2.3 Hz, 1H), 6.78 (s, 1H), 6.65 (s, 1H), 6.42 (t, J = 2.1 Hz, 1H), 5.06 (brs, 1H), 4.79 – 4.69 (m, 1H), 4.10 (dd, J = 10.3, 4.6 Hz, 1H), 3.97 (dd, J = 10.2, 3.6 Hz, 1H), 3.47 (t, J = 8.8 Hz, 1H), 3.31 – 3.23 (m, 1H), 2.36 (s, 3H), 2.04 (s, 3H). **^{13}C NMR** (100 MHz, $CDCl_3$) δ 159.0, 153.9, 140.2, 140.0, 137.7, 132.3, 127.5, 124.6, 112.2, 105.8, 74.0, 69.1, 42.1, 21.7, 17.3. **HRMS (ESI)** m/z Calc. for $C_{15}H_{18}N_3O_3$ $[M + H]^+$ 228.1343; Found 228.1348.

The spectroscopic data are in agreement with the literature.[15]

10. NMR spectra

1 - ^{1}H NMR (400 MHz, $CDCl_3$)

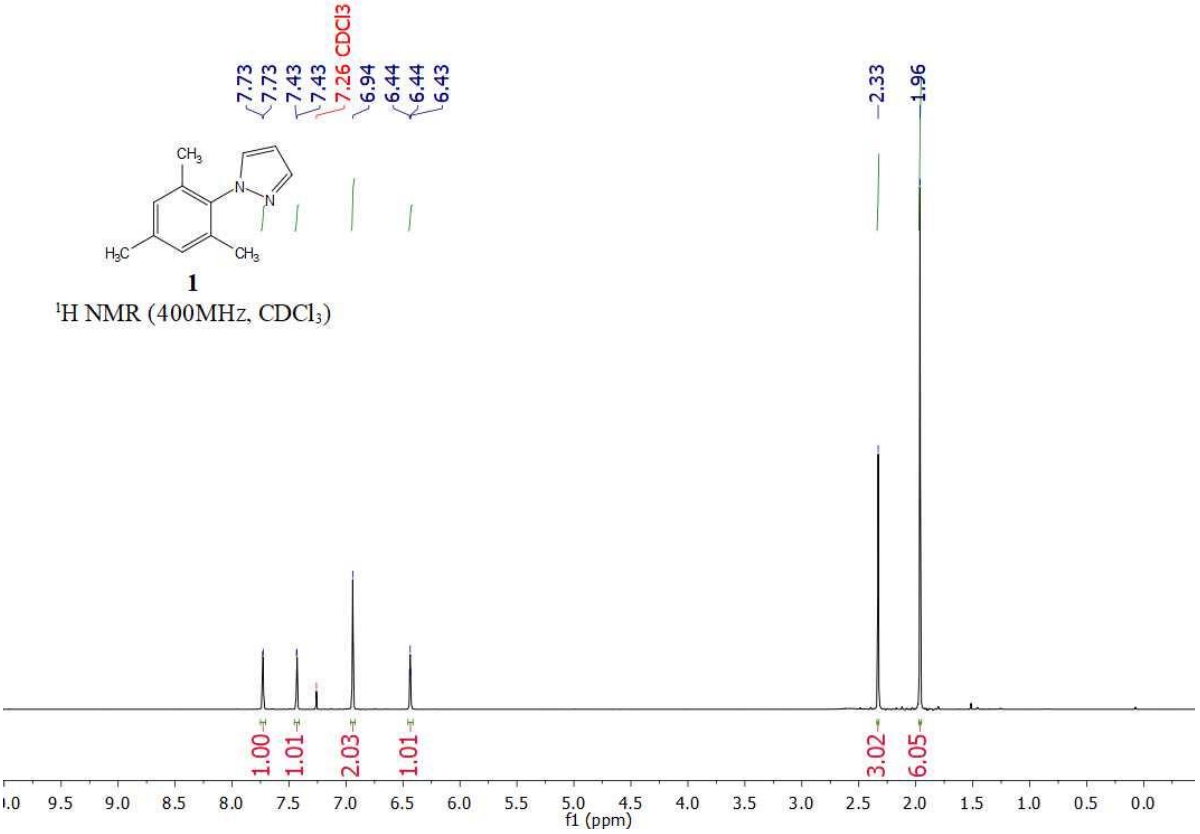

1 – ^{13}C NMR (100 MHz, $CDCl_3$)

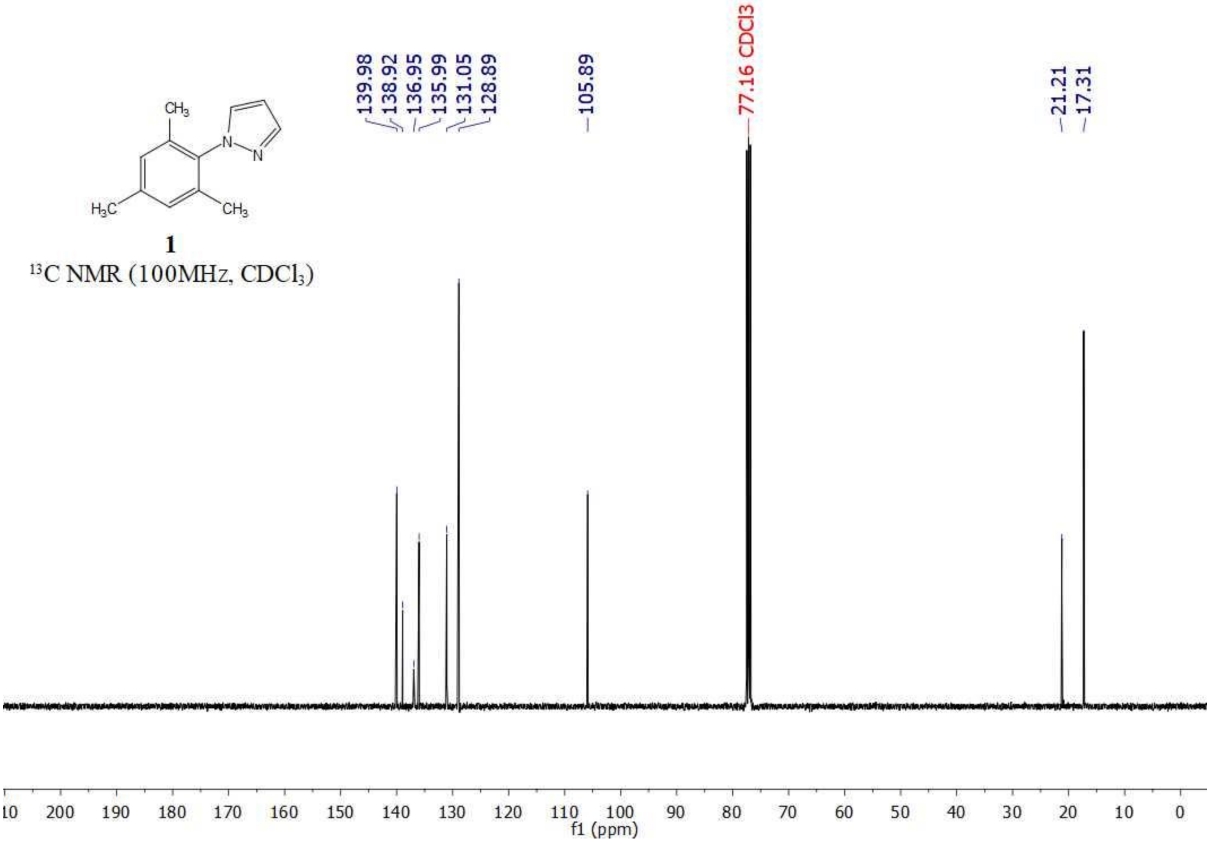

2 - ^{1}H NMR (400 MHz, $CDCl_3$)

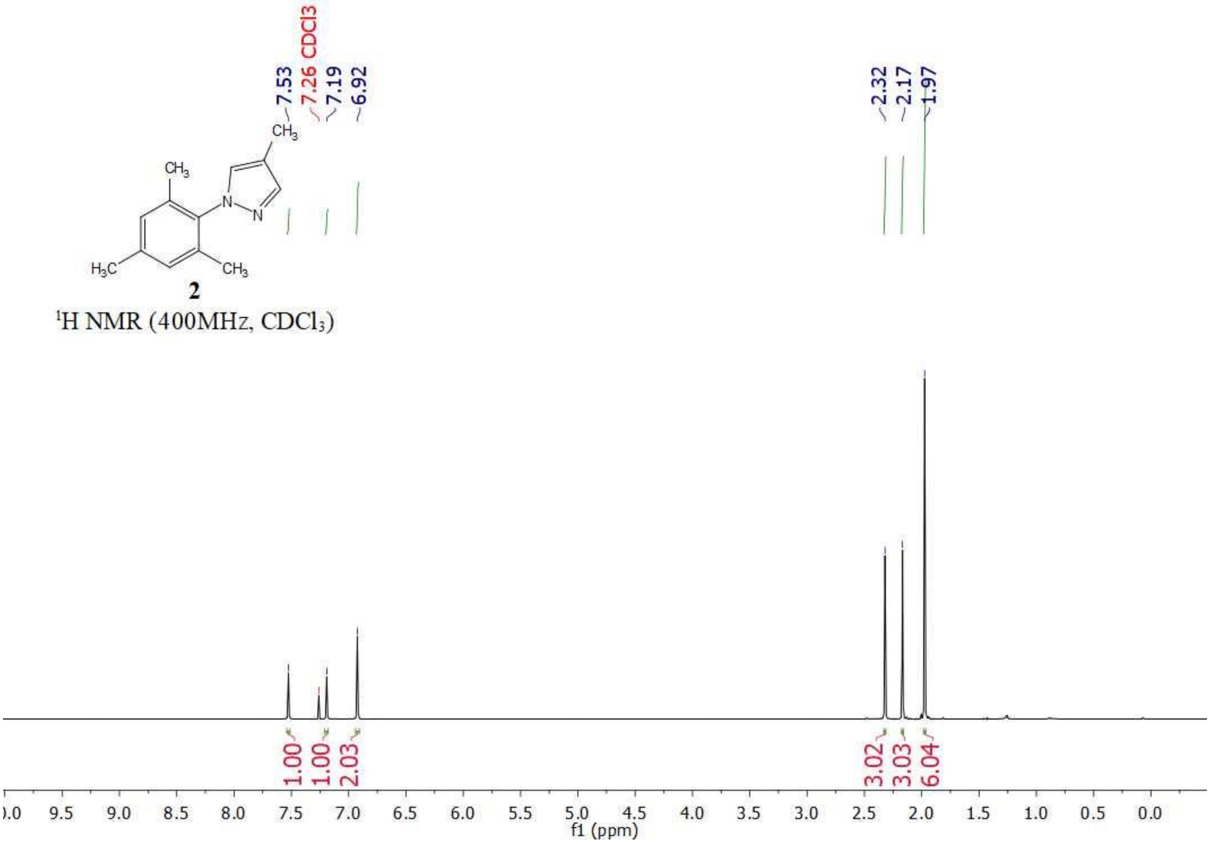

2 – ^{13}C NMR (100 MHz, $CDCl_3$)

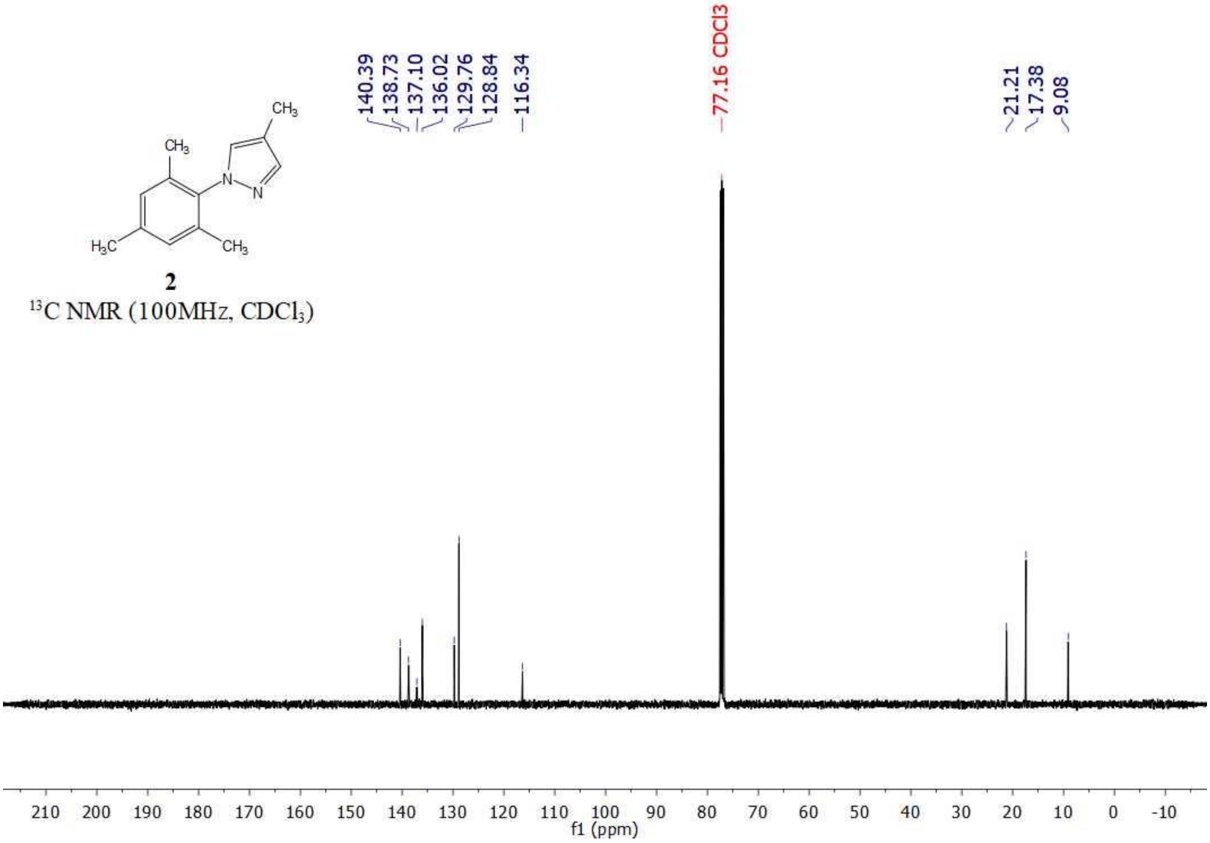

3 - ^{1}H NMR (400 MHz, $CDCl_3$)

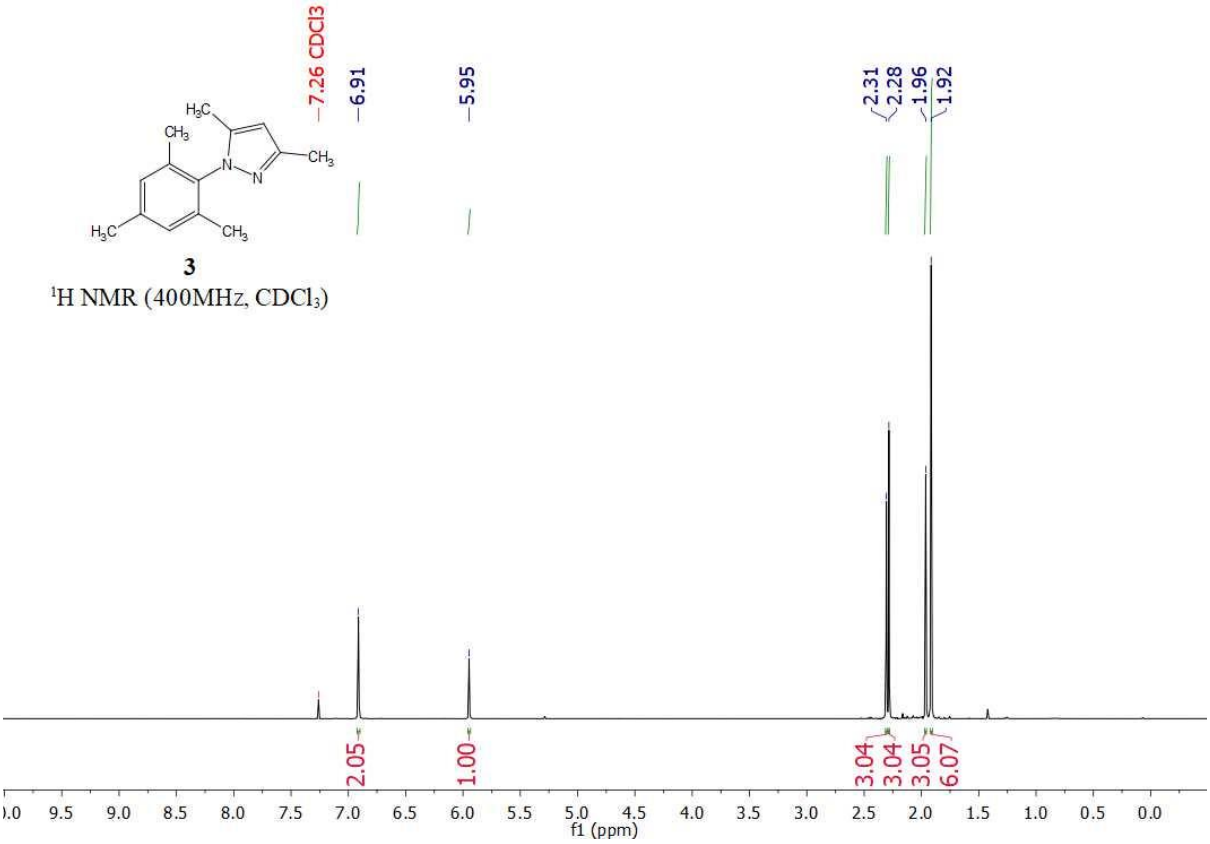

3 – ^{13}C NMR (100 MHz, $CDCl_3$)

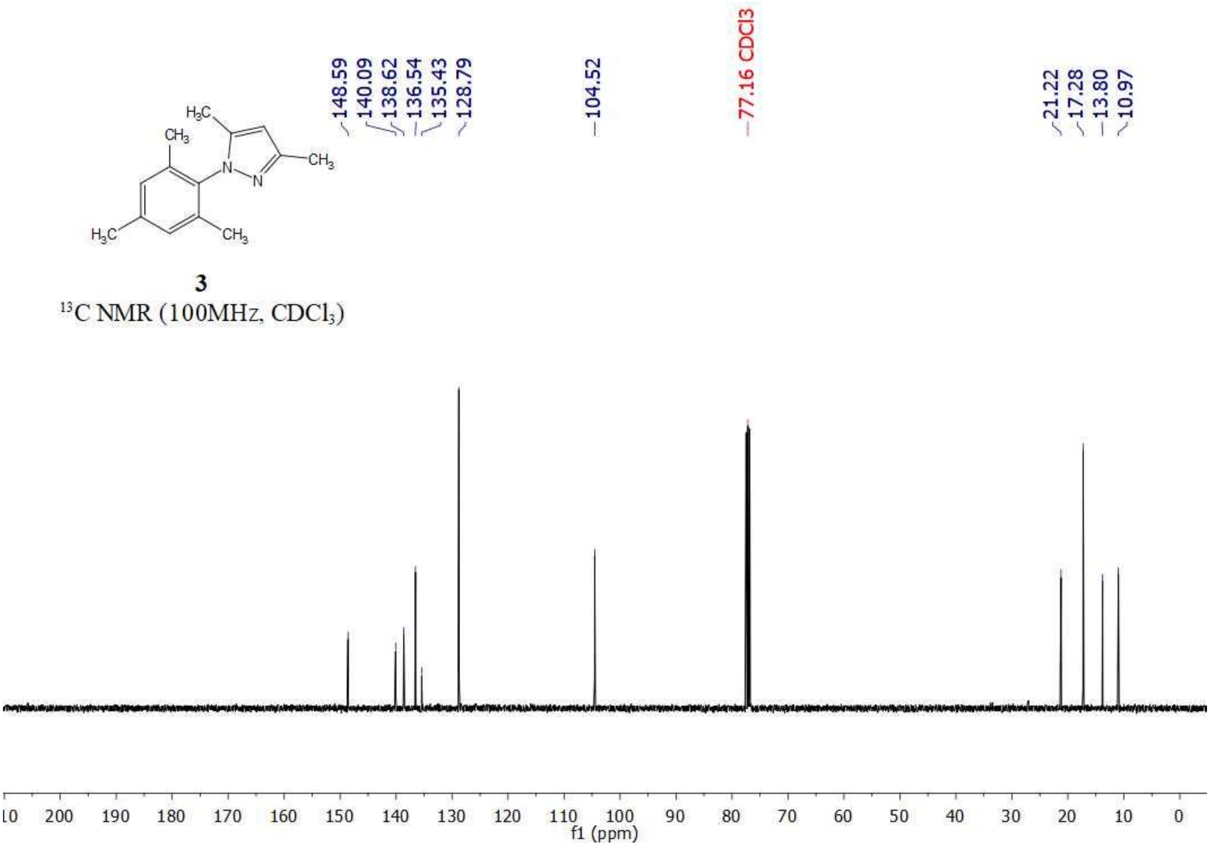

4 - ^{1}H NMR (400 MHz, $CDCl_3$)

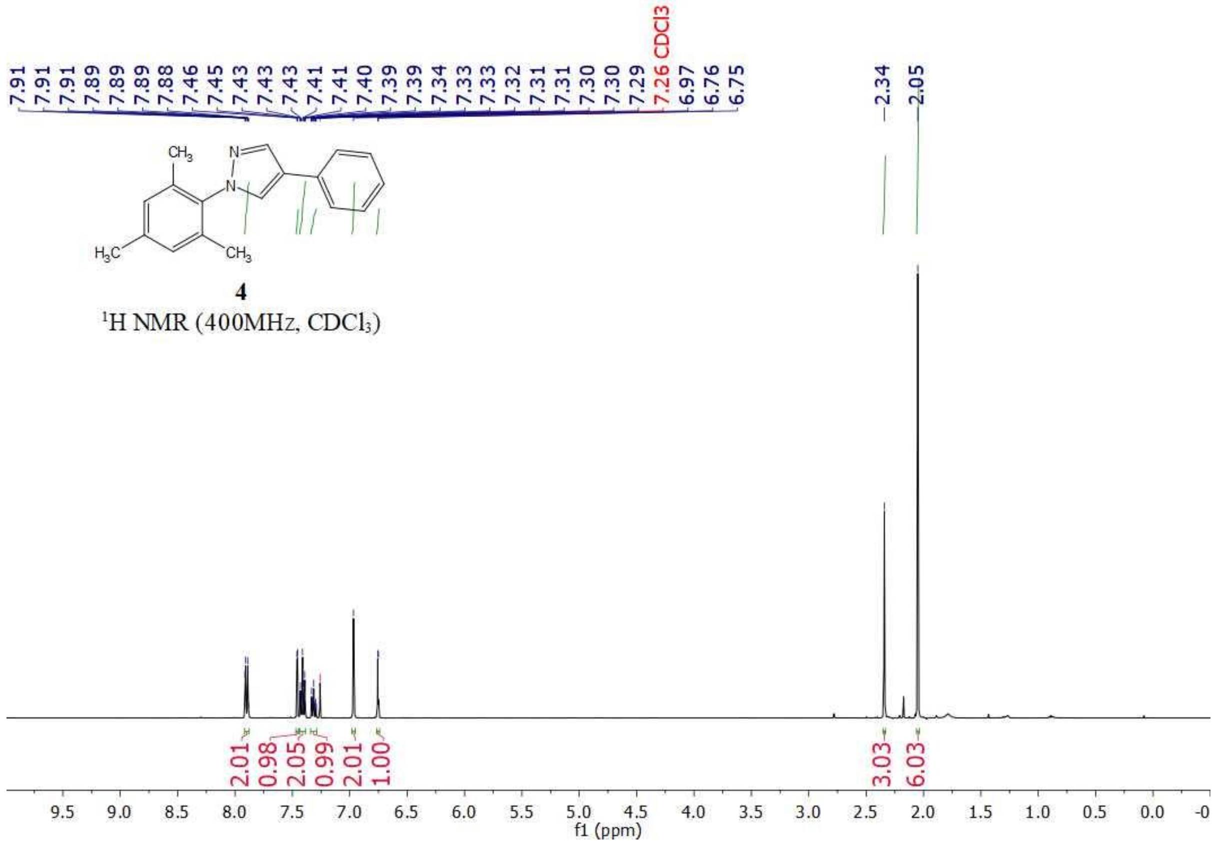

4 – ^{13}C NMR (100 MHz, $CDCl_3$)

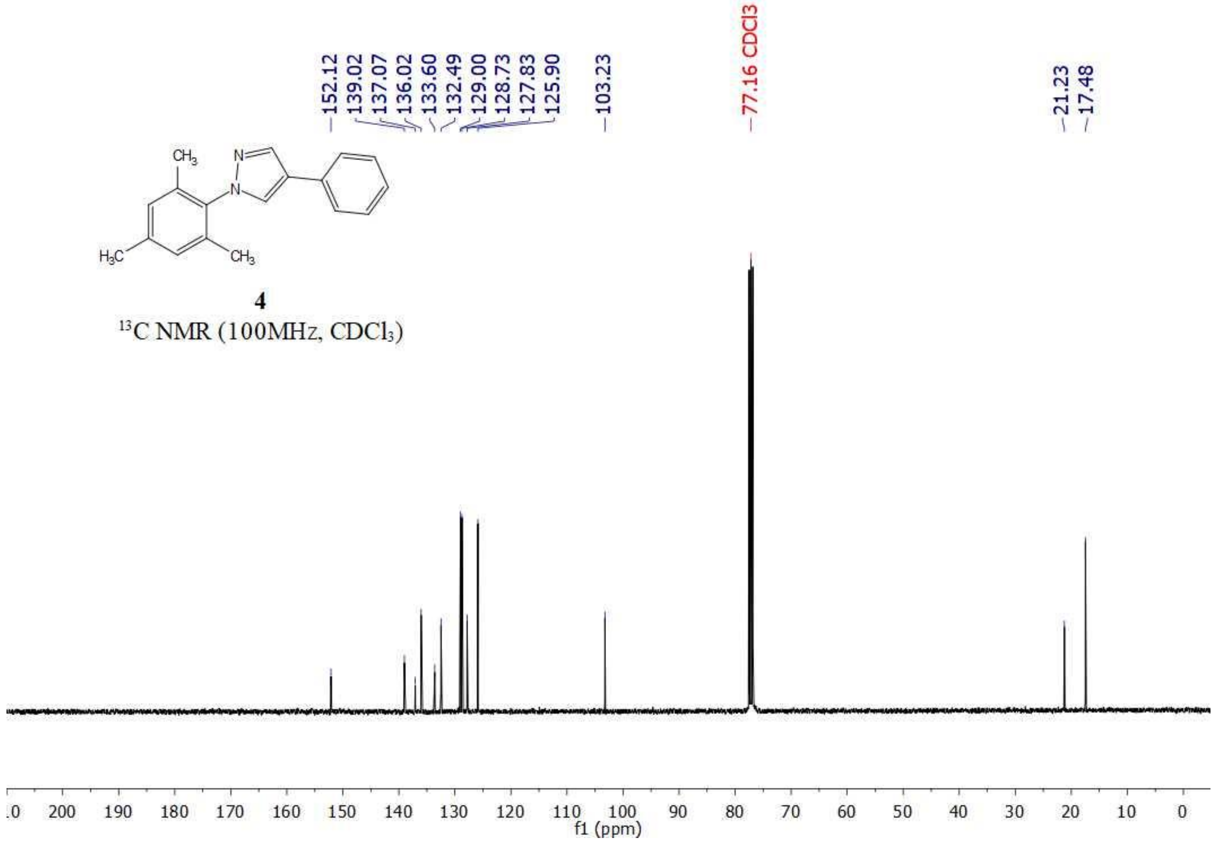

5 - ^{1}H NMR (400 MHz, $CDCl_3$)

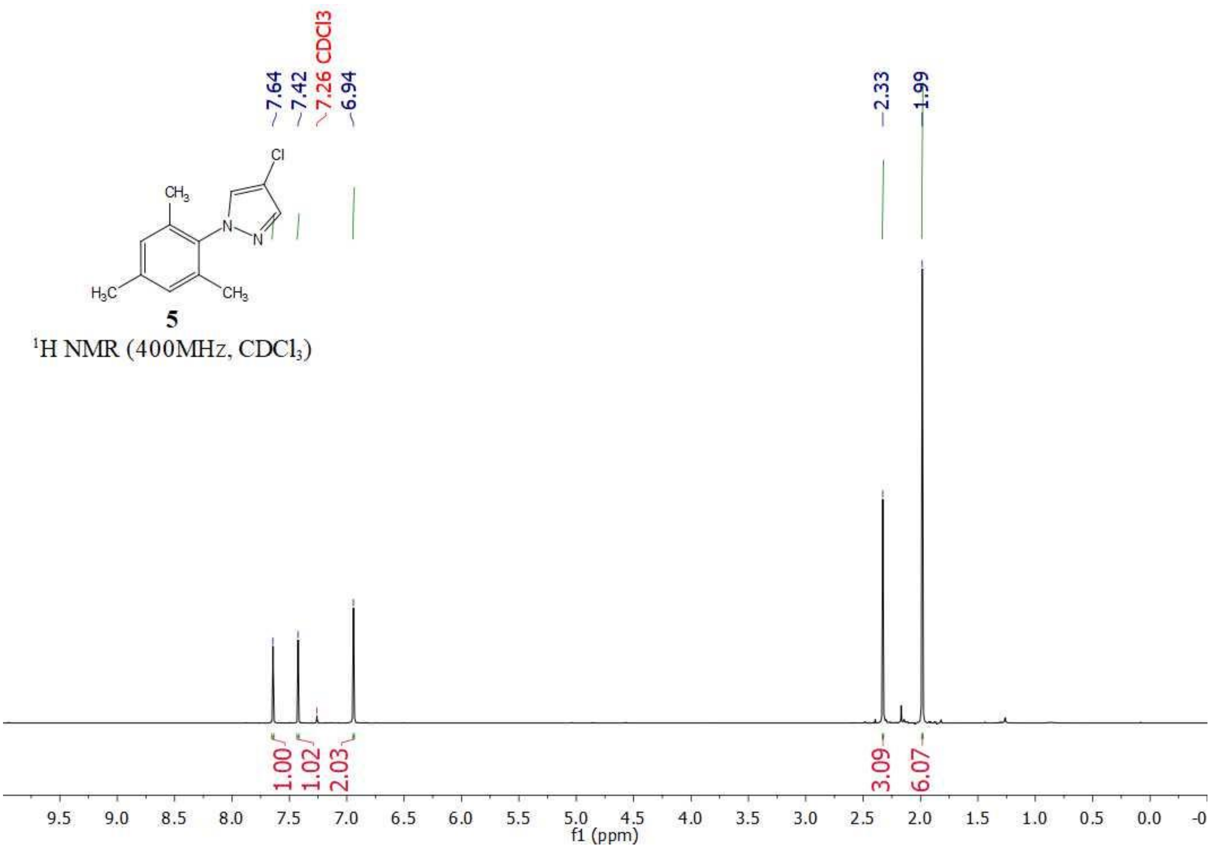

5 – ^{13}C NMR (100 MHz, $CDCl_3$)

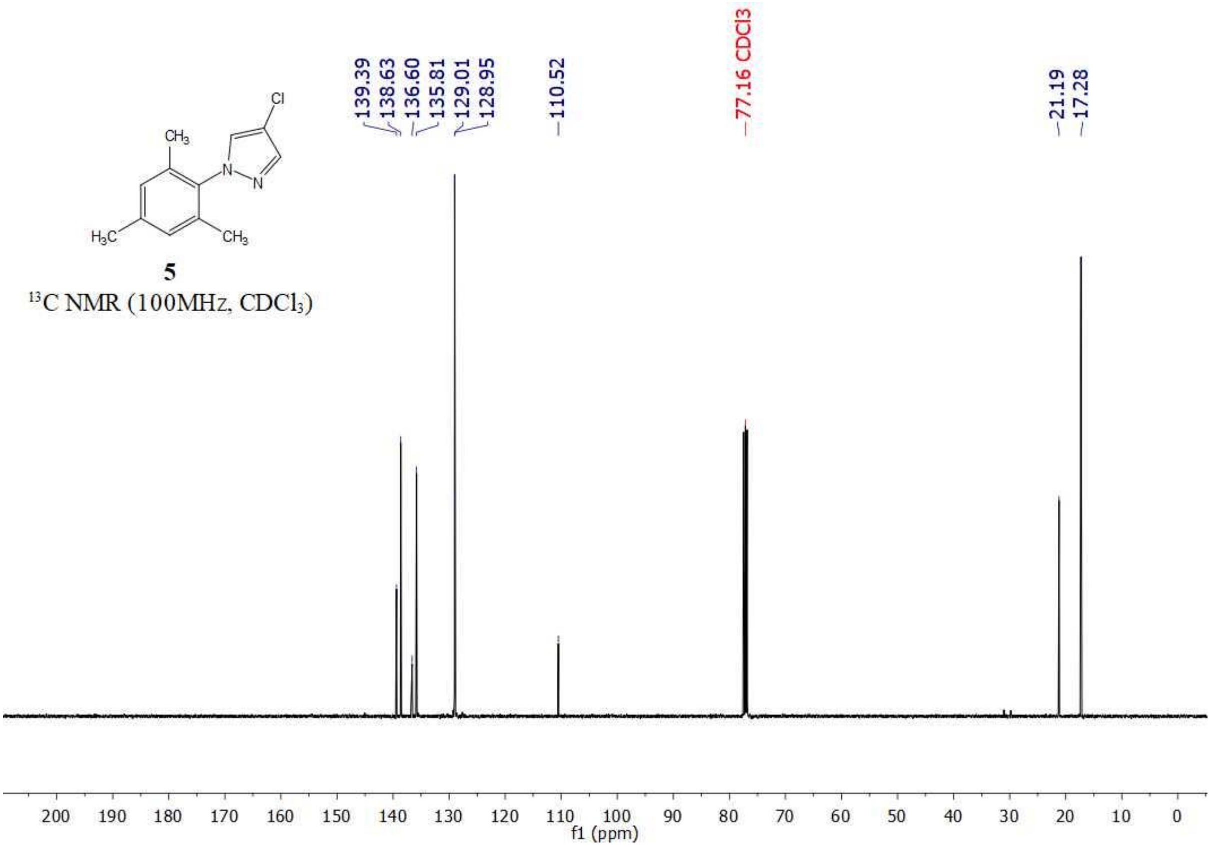

6 - ^{1}H NMR (400 MHz, $CDCl_3$)

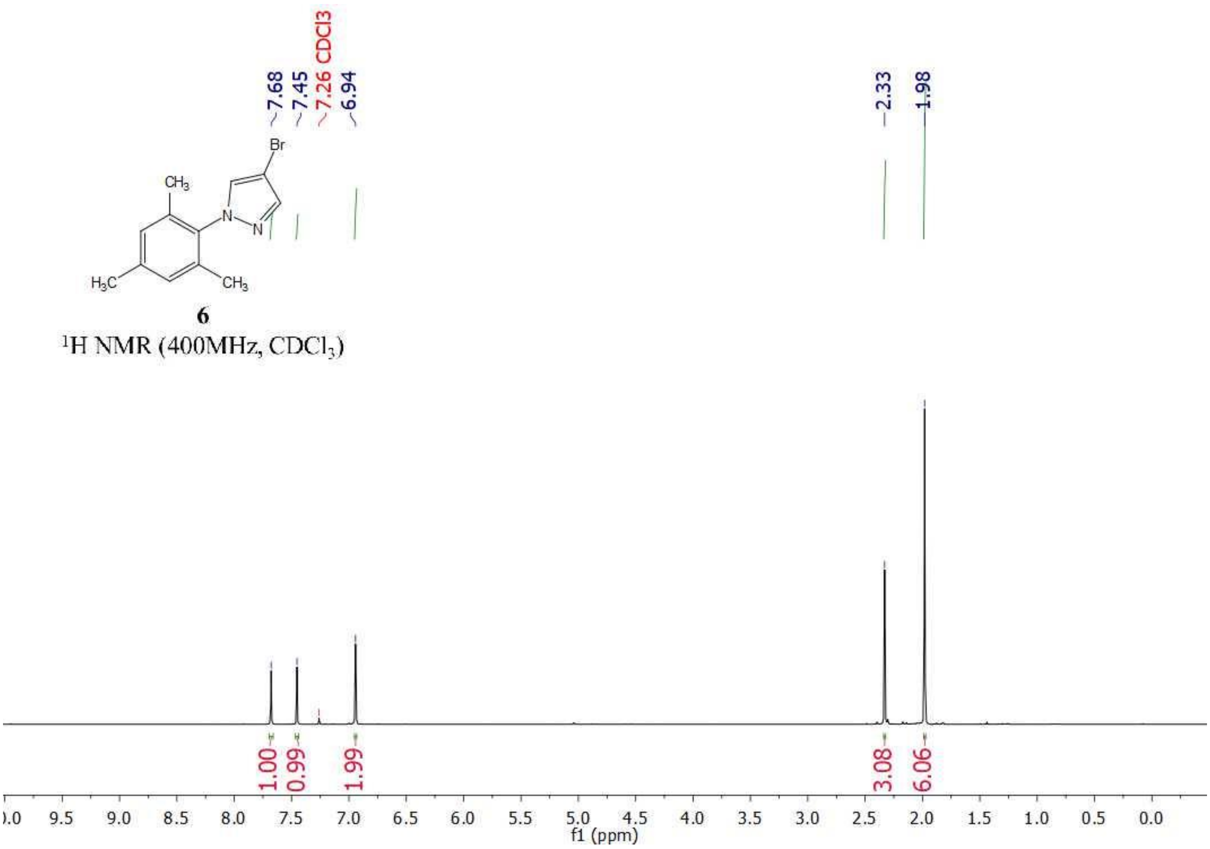

6 – ^{13}C NMR (100 MHz, $CDCl_3$)

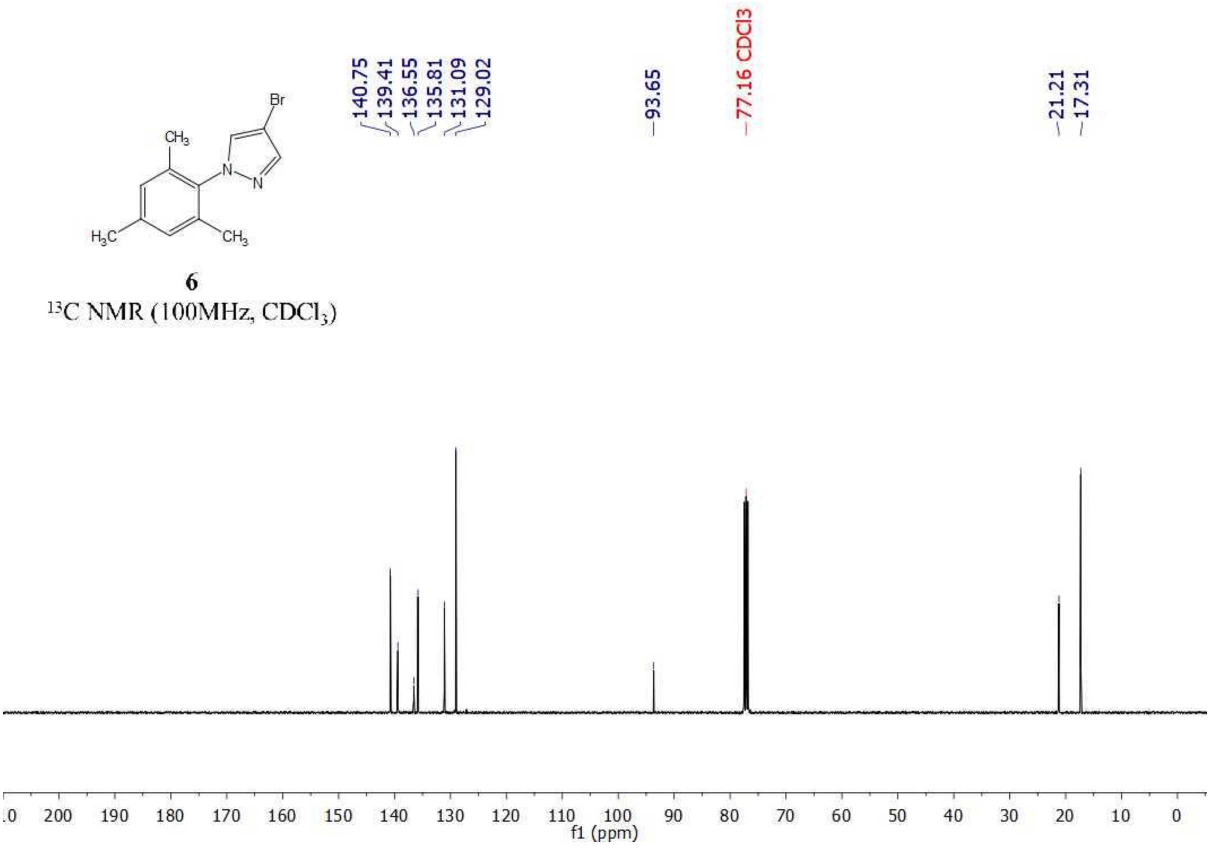

7 - ^{1}H NMR (400 MHz, $CDCl_3$)

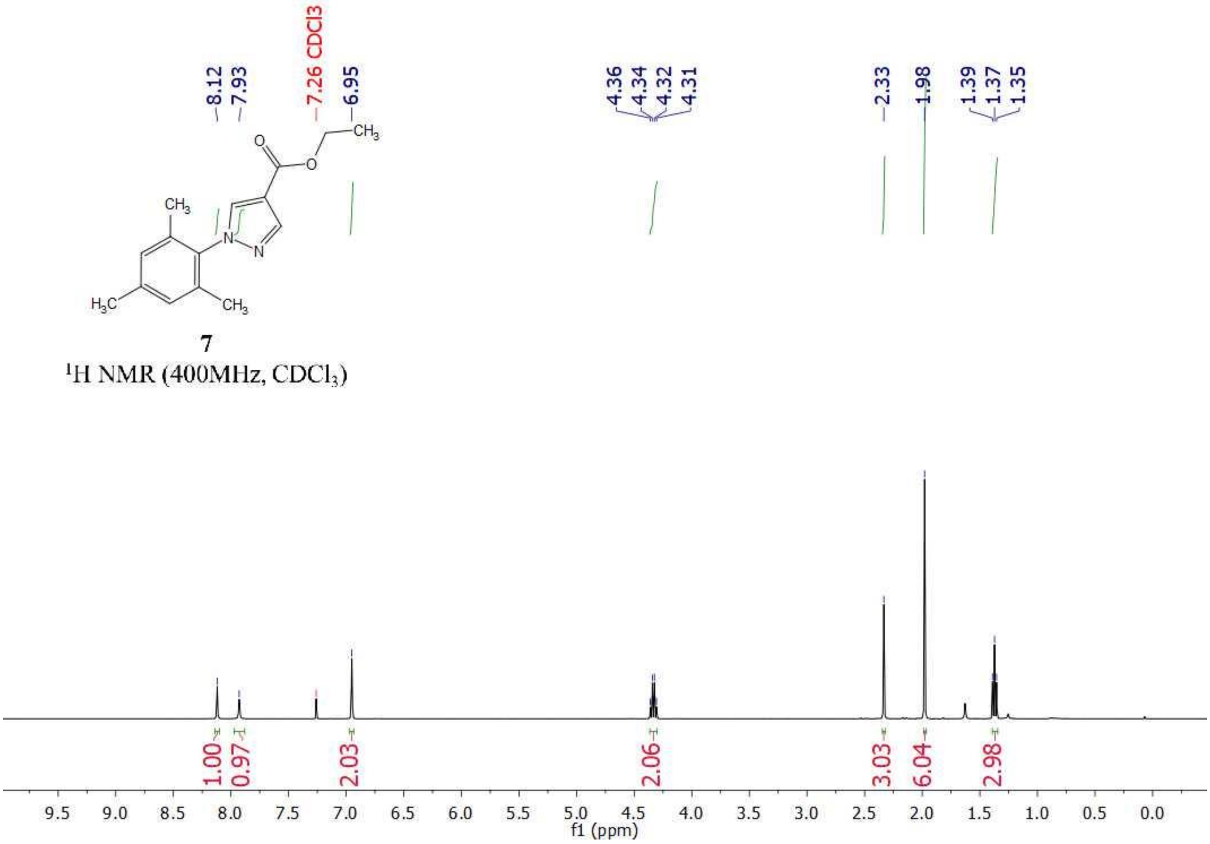

7 – ^{13}C NMR (100 MHz, $CDCl_3$)

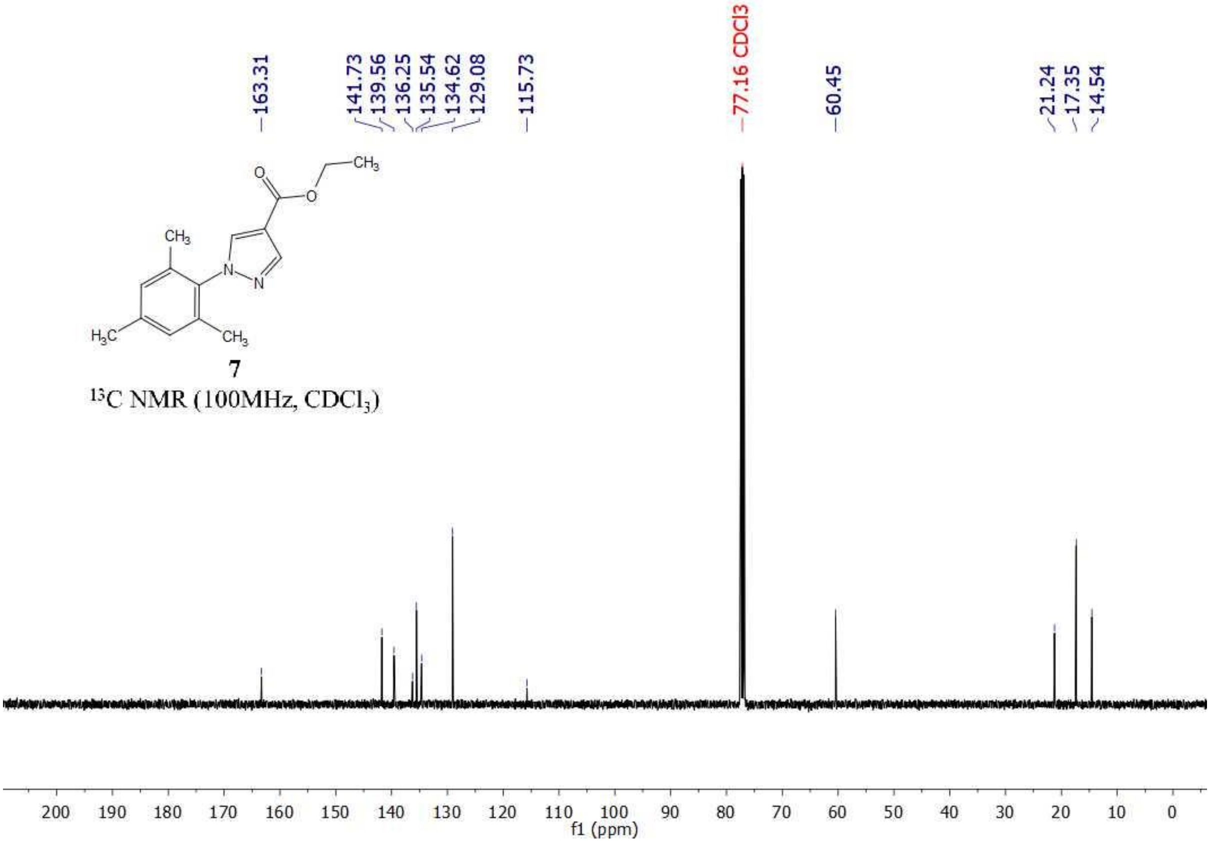

8 - ^{1}H NMR (400 MHz, $CDCl_3$)

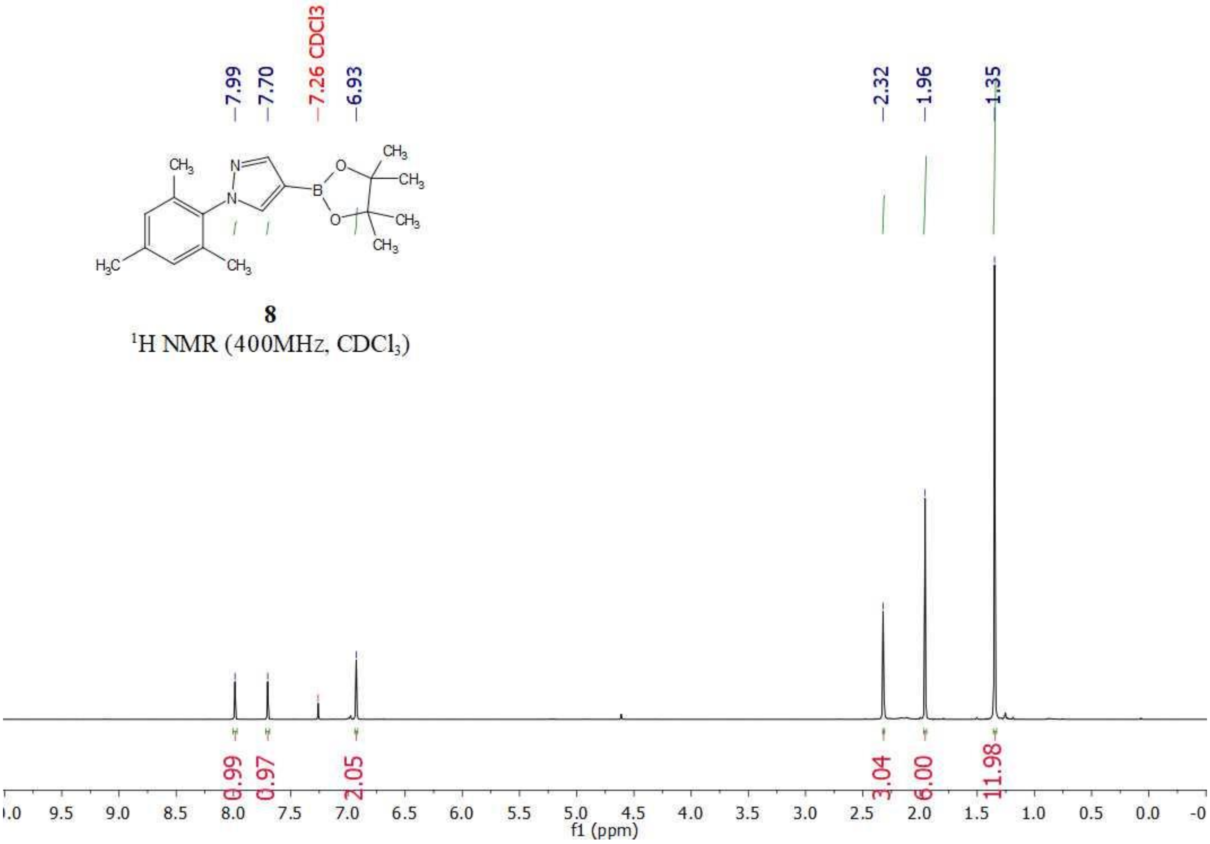

8 – ^{13}C NMR (100 MHz, $CDCl_3$)

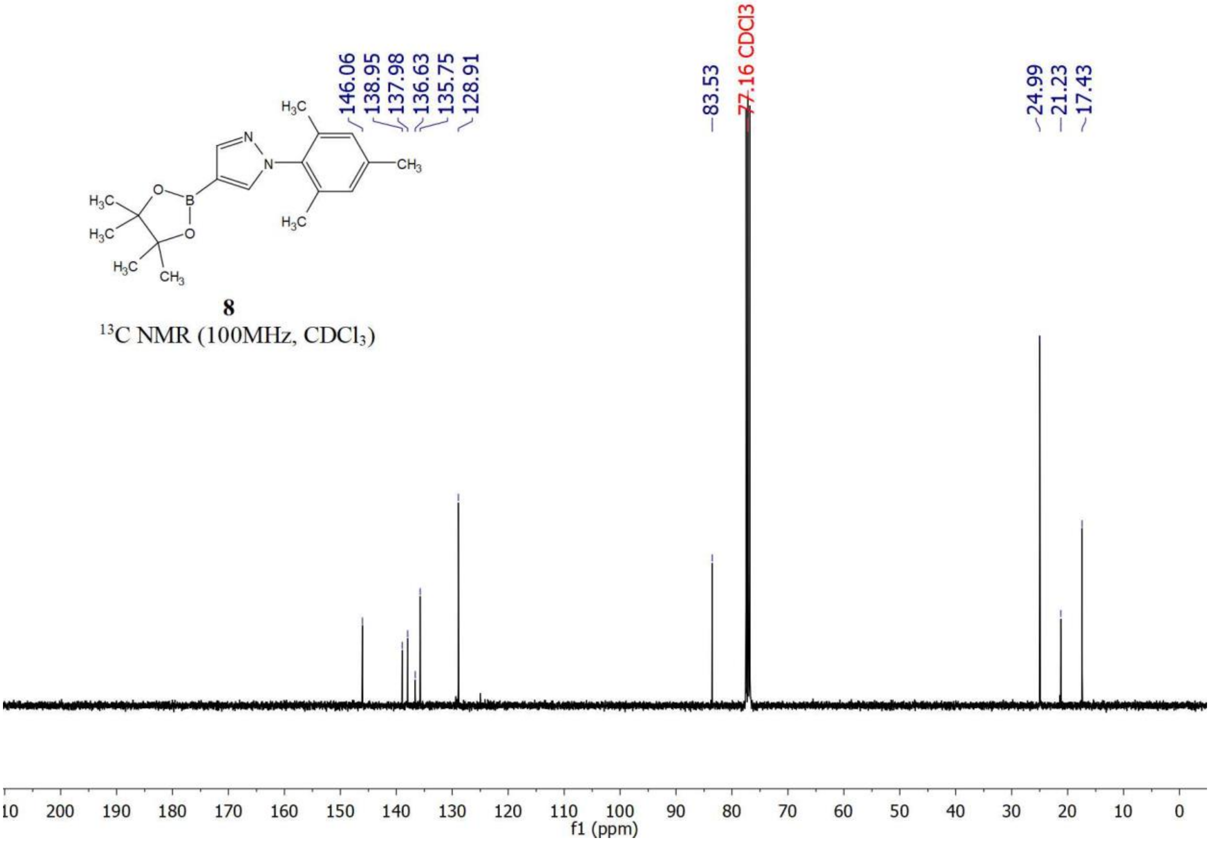

9 - ^{1}H NMR (400 MHz, $CDCl_3$)

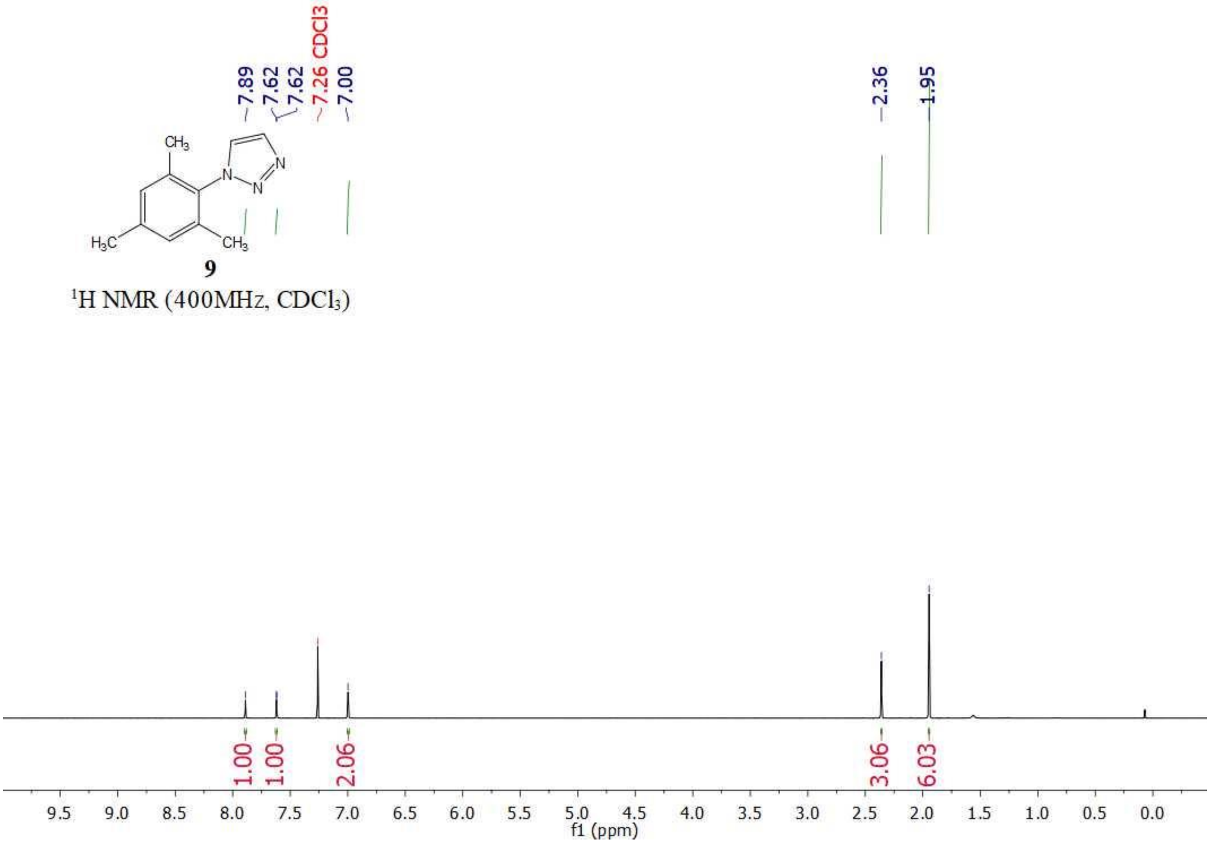

9 – ^{13}C NMR (100 MHz, $CDCl_3$)

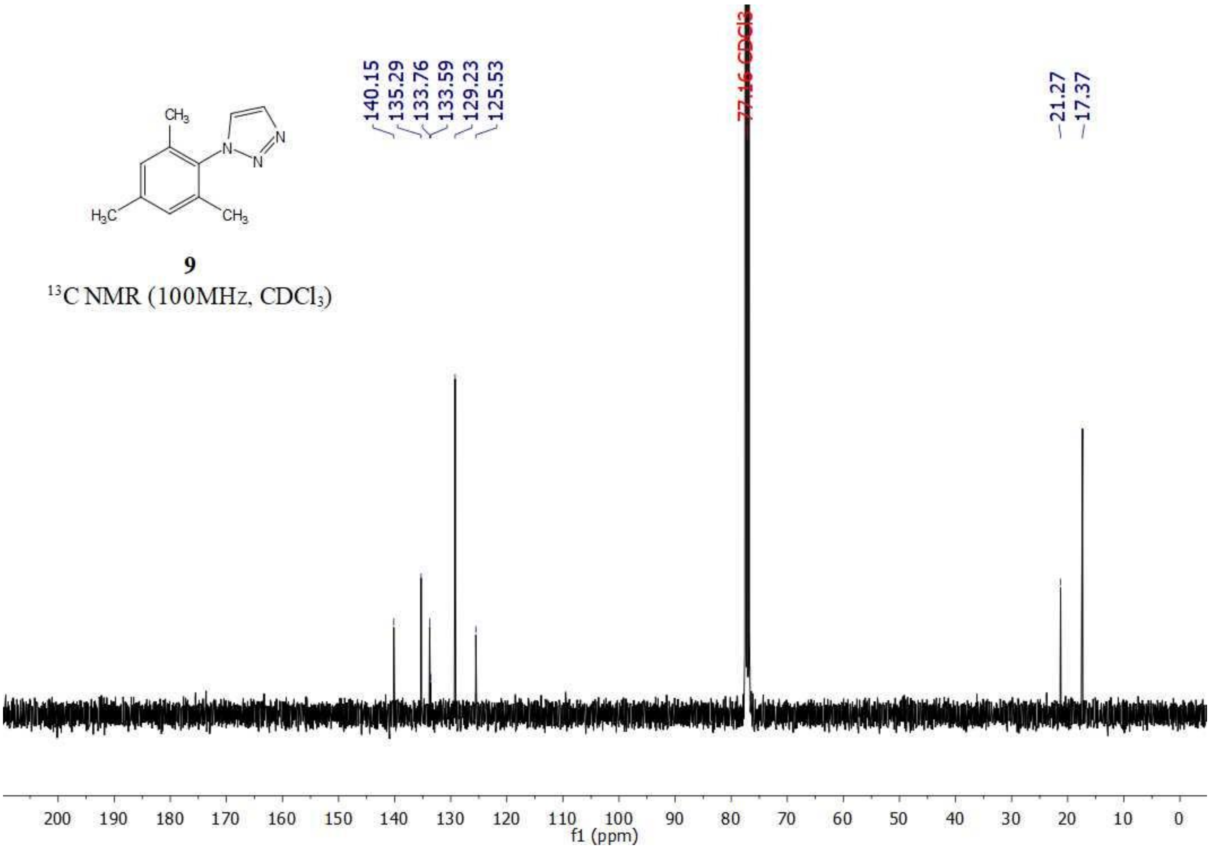

10 - ^{1}H NMR (400 MHz, $CDCl_3$)

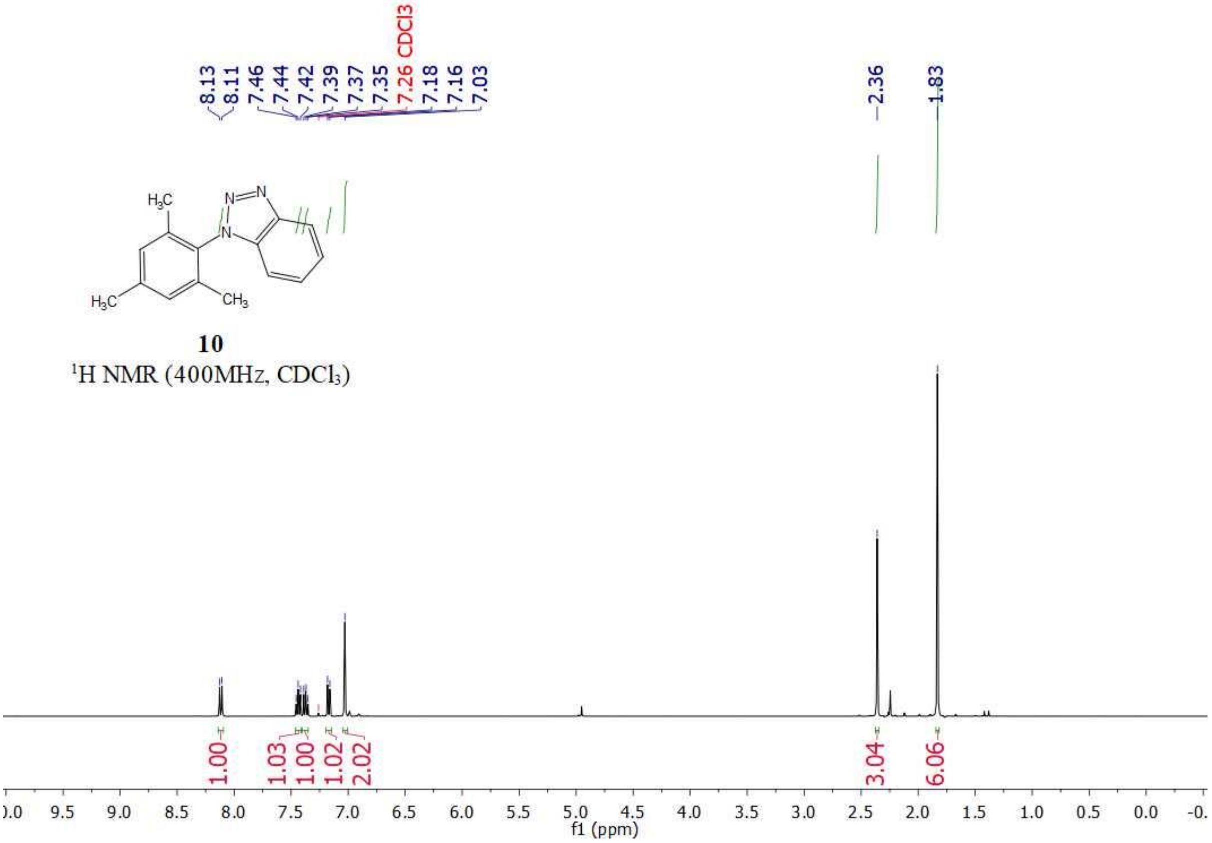

10 – ^{13}C NMR (100 MHz, $CDCl_3$)

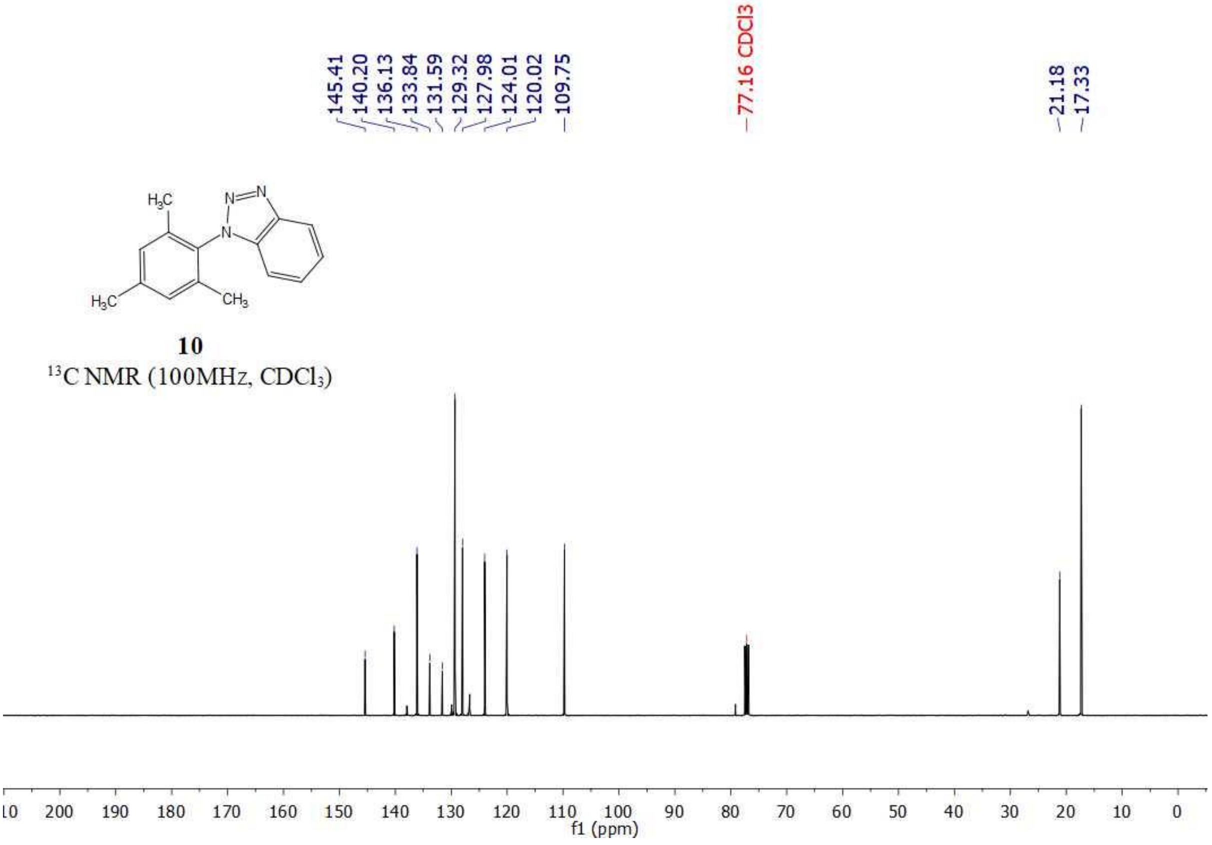

11 - ^{1}H NMR (400 MHz, $CDCl_3$)

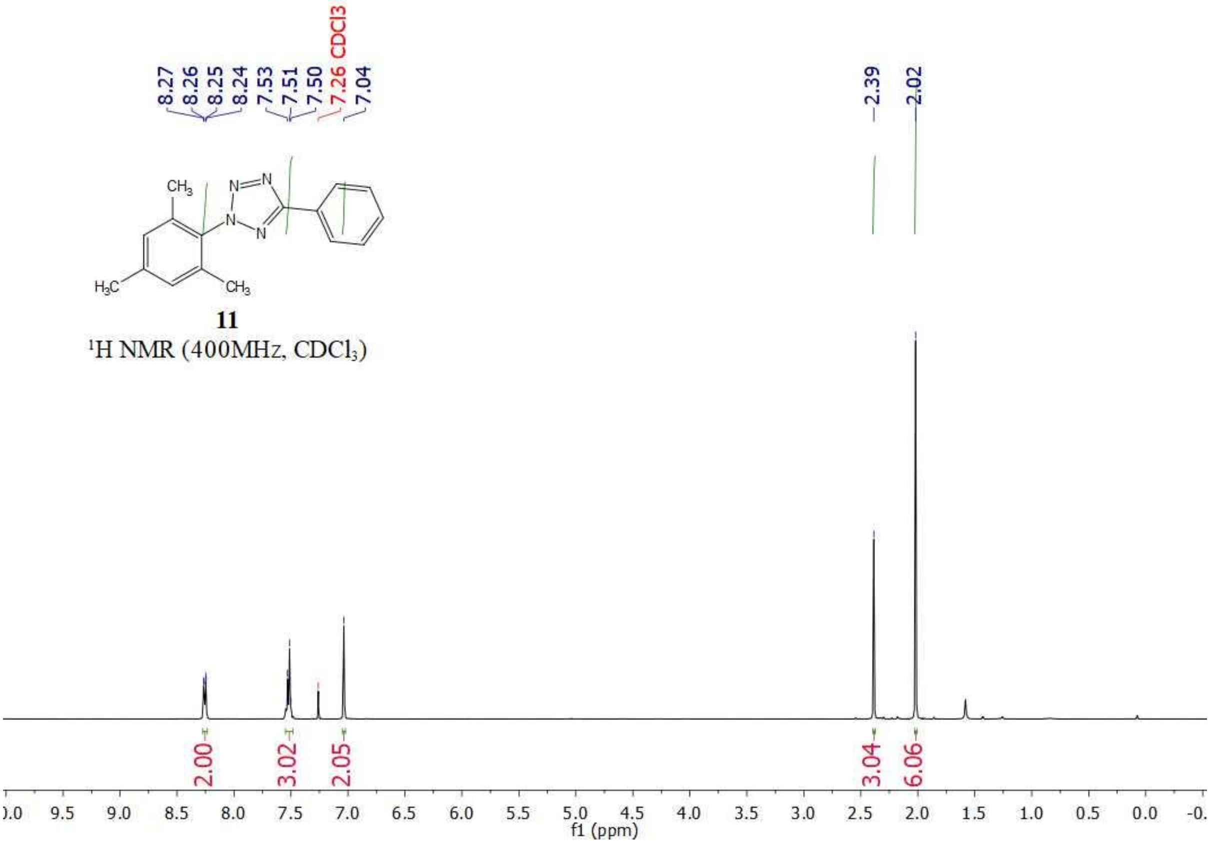

11 – ^{13}C NMR (100 MHz, $CDCl_3$)

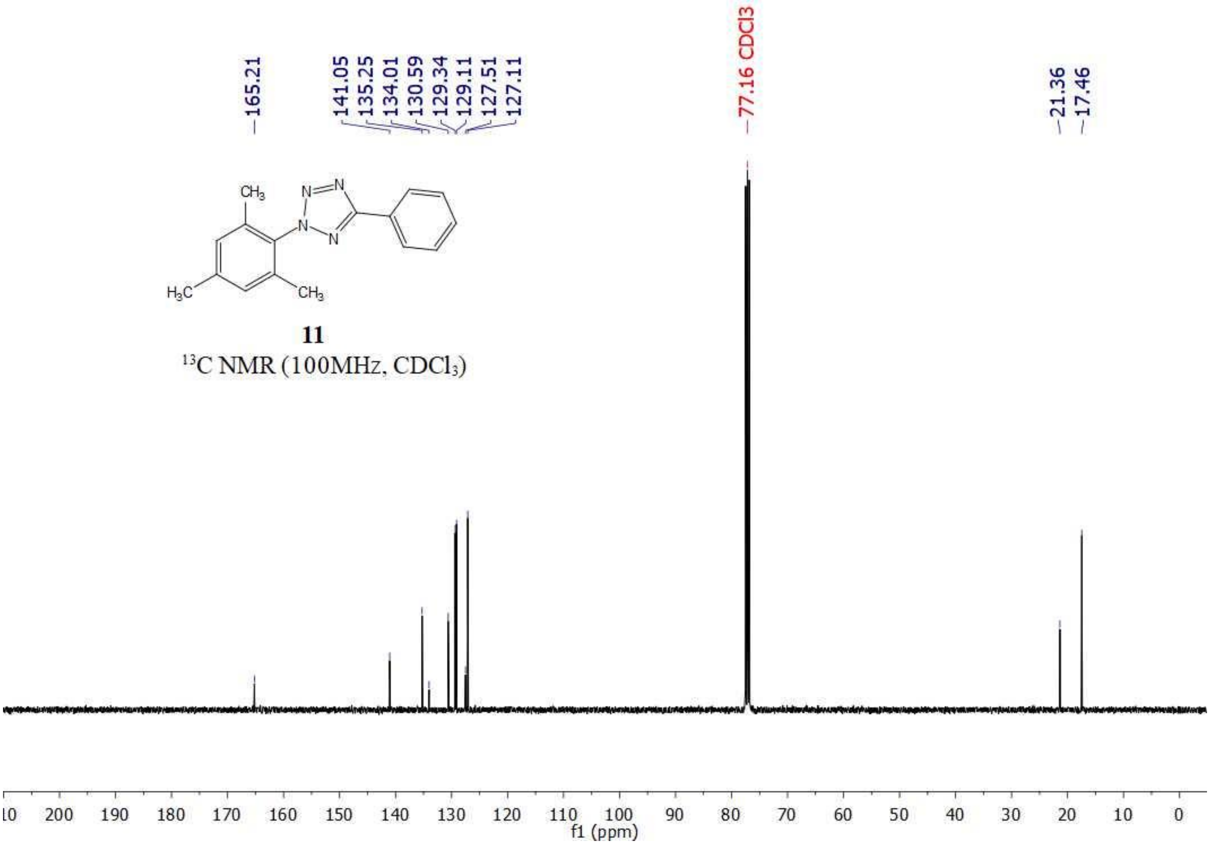

11’ - ^{1}H NMR (400 MHz, $CDCl_3$)

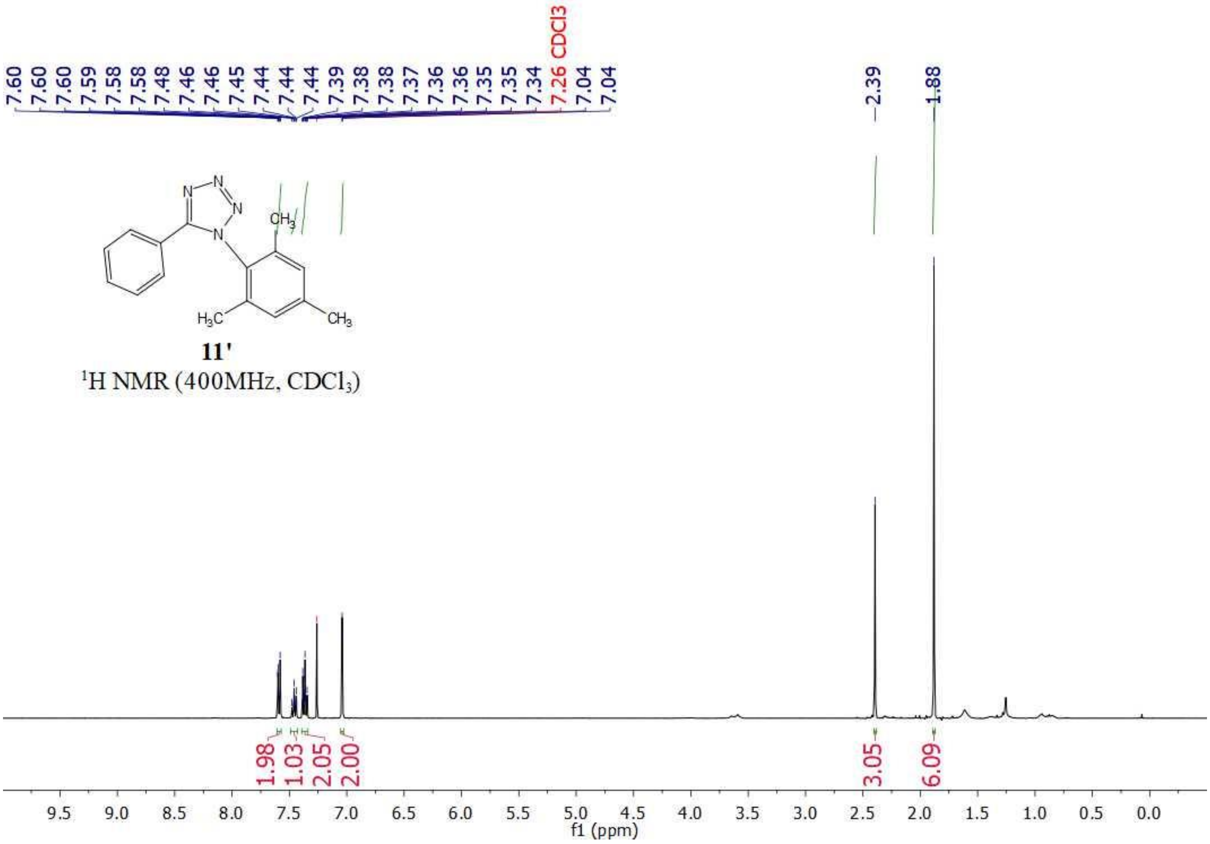

11’ – ^{13}C NMR (100 MHz, $CDCl_3$)

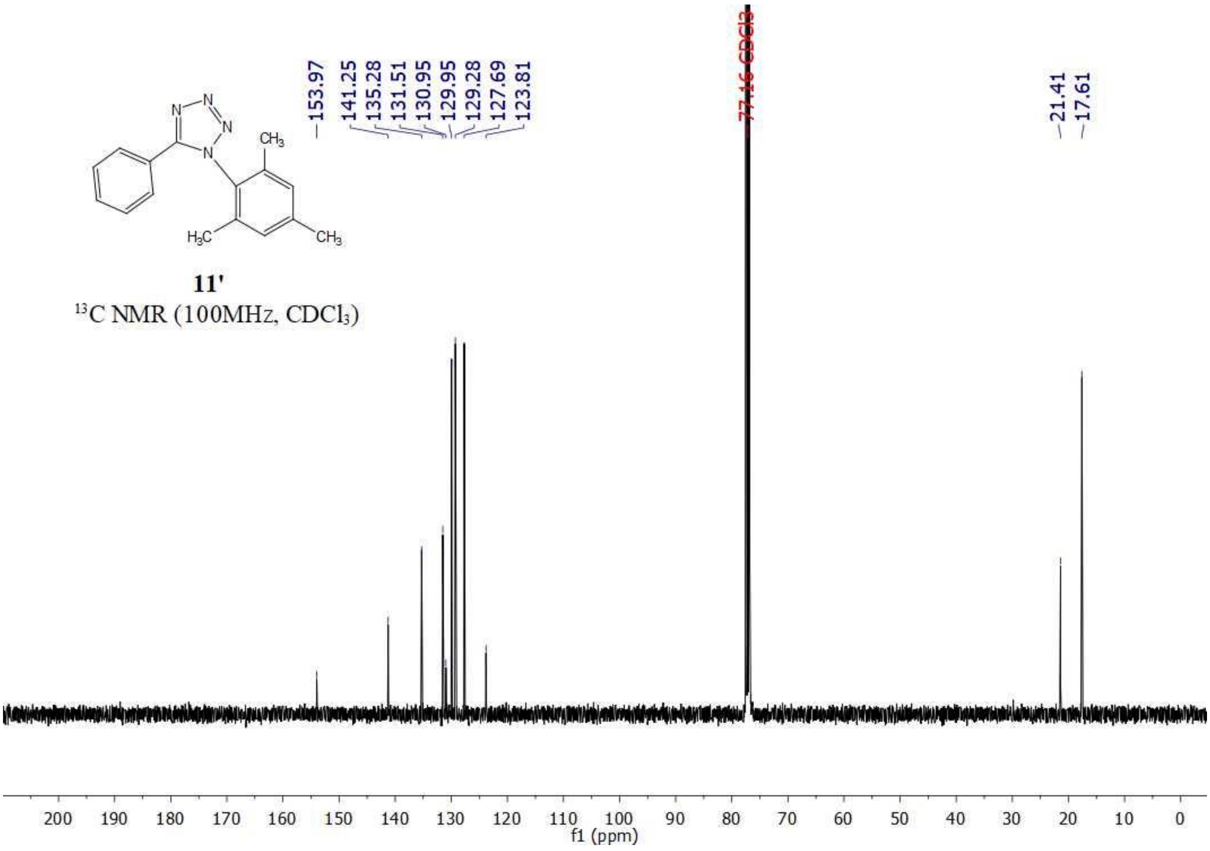

12 - ^{1}H NMR (400 MHz, $CDCl_3$)

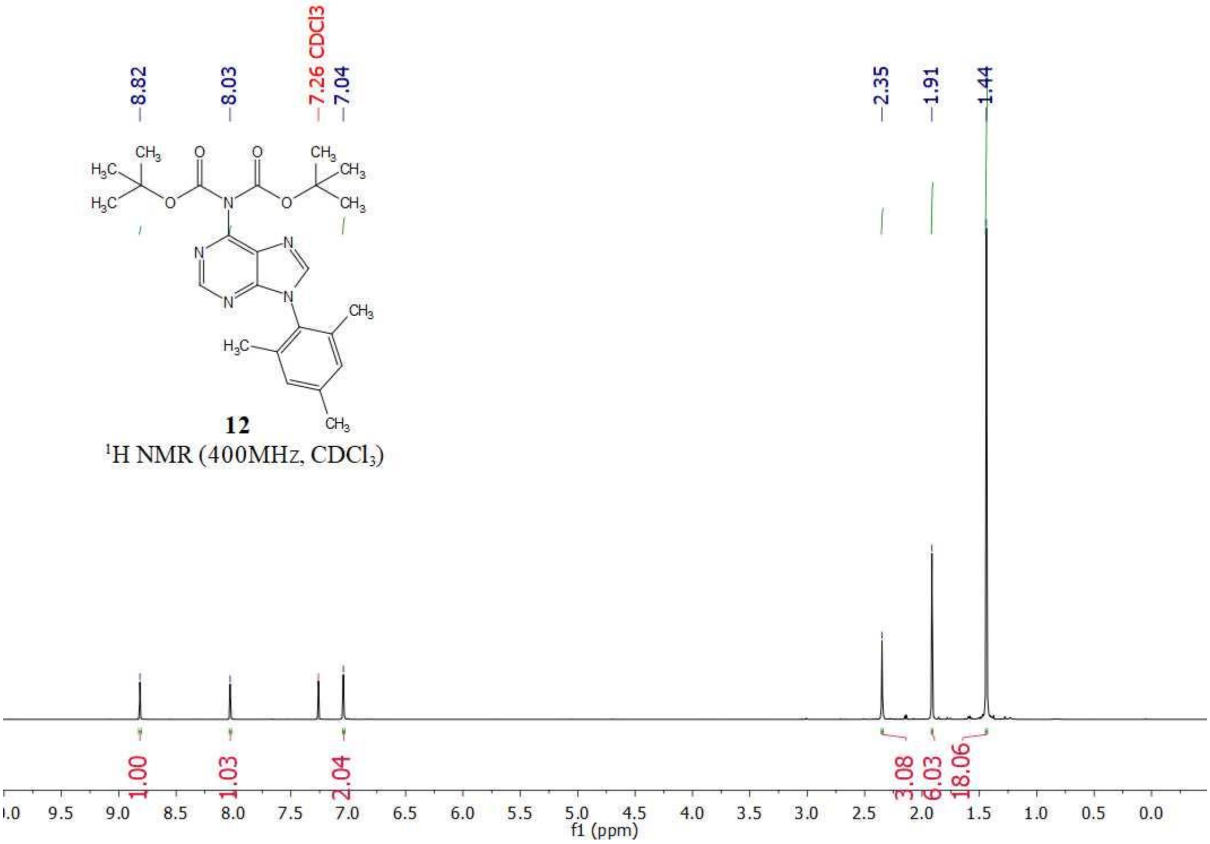

12 – ^{13}C NMR (100 MHz, $CDCl_3$)

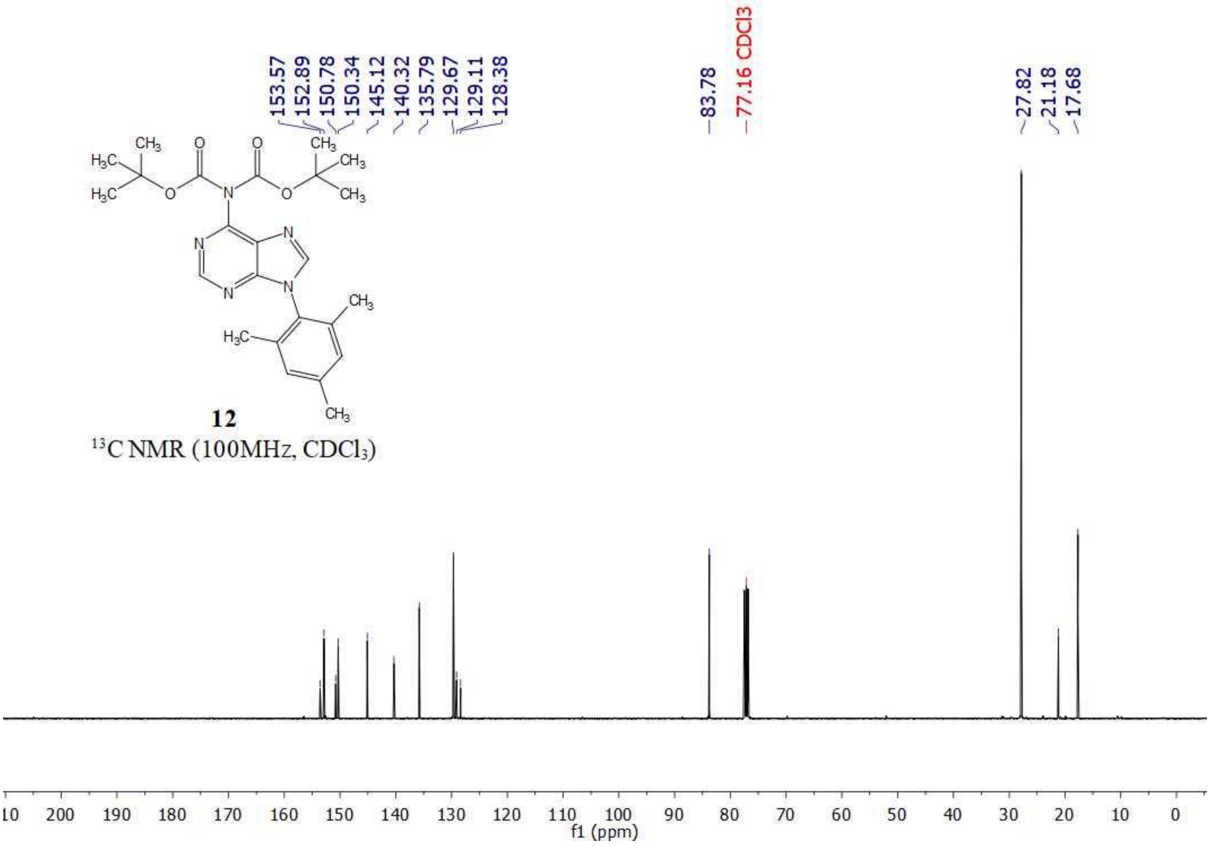

13 - ^{1}H NMR (400 MHz, $CDCl_3$)

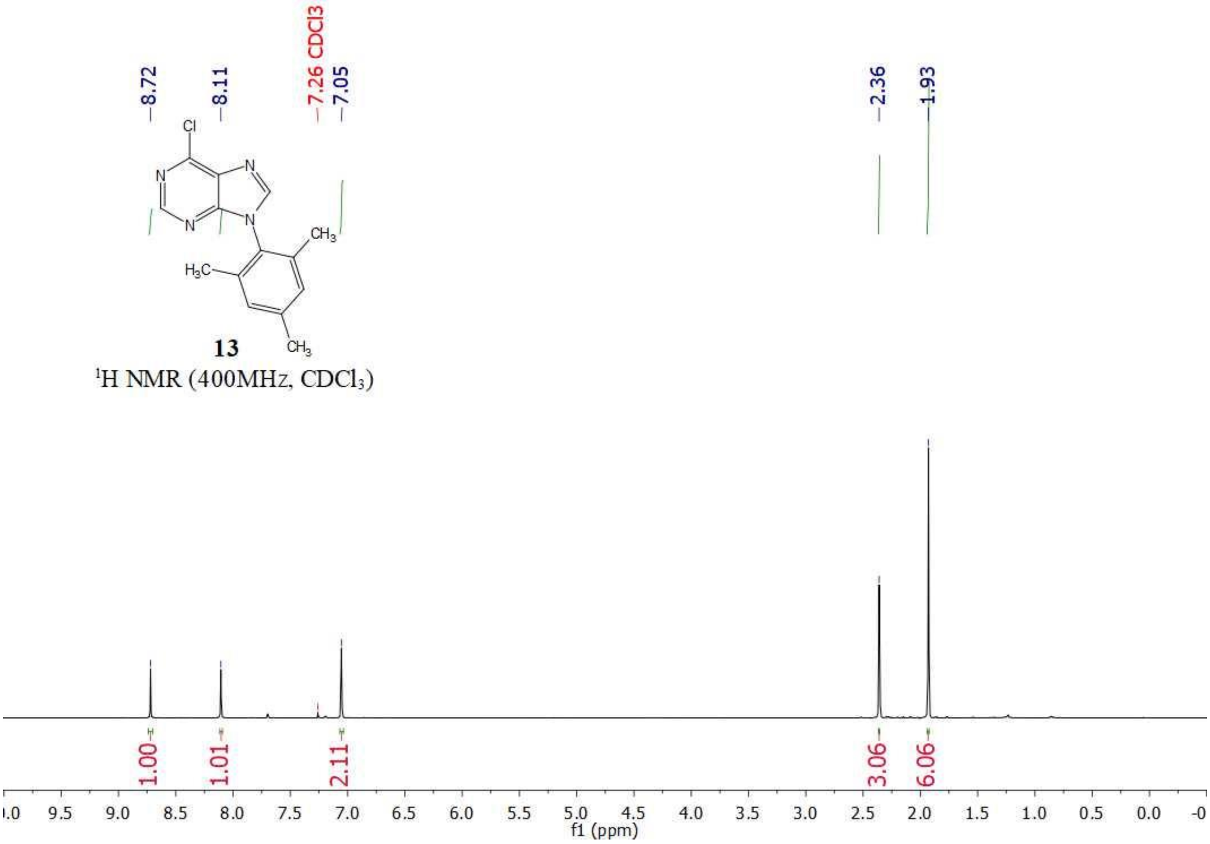

13 – ^{13}C NMR (100 MHz, $CDCl_3$)

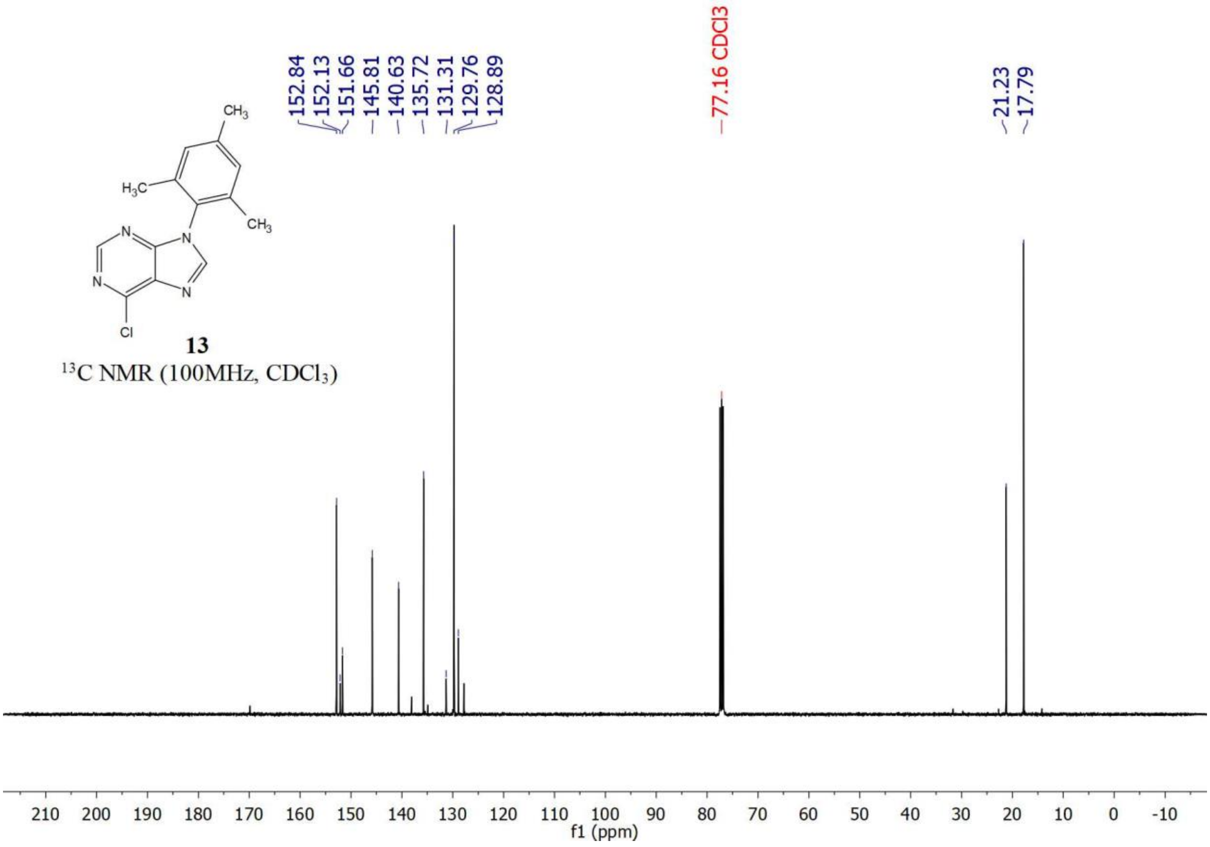

14 - ^{1}H NMR (400 MHz, $CDCl_3$)

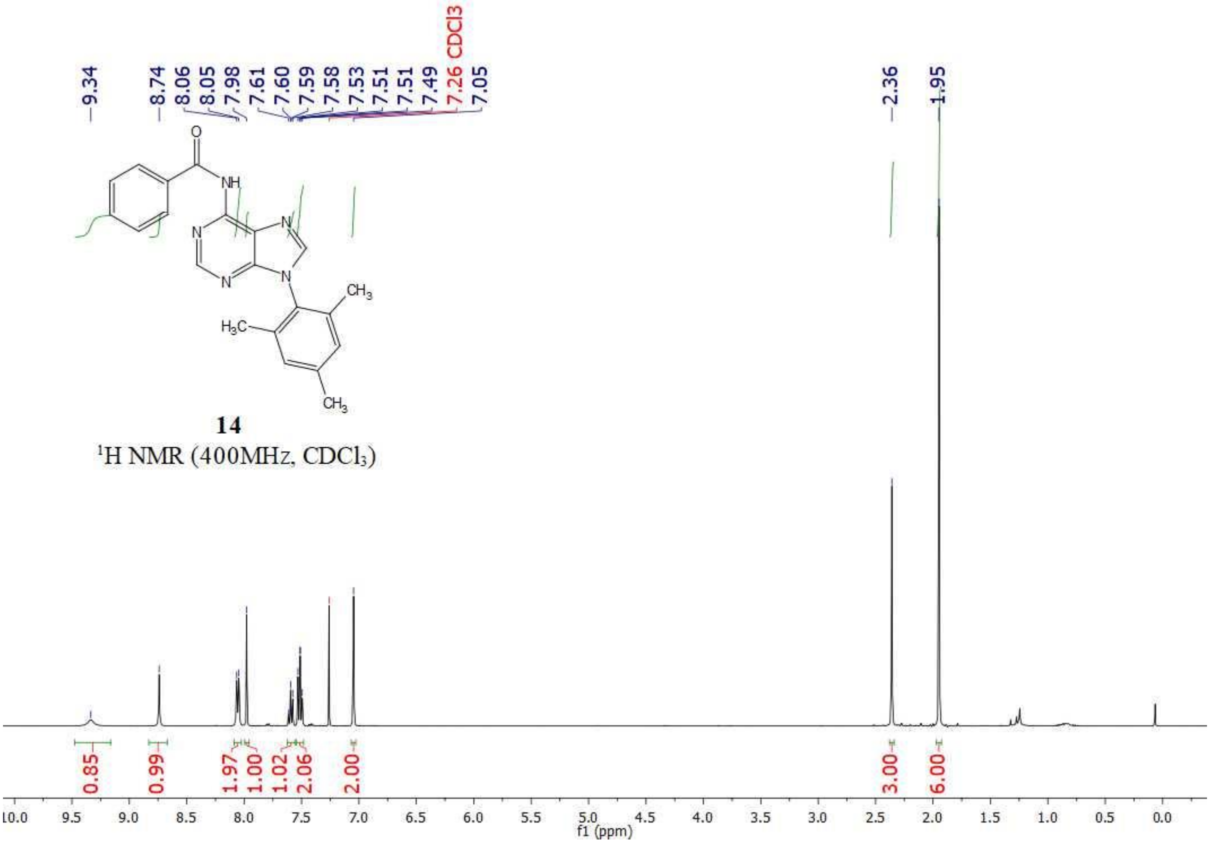

14 – ^{13}C NMR (100 MHz, $CDCl_3$)

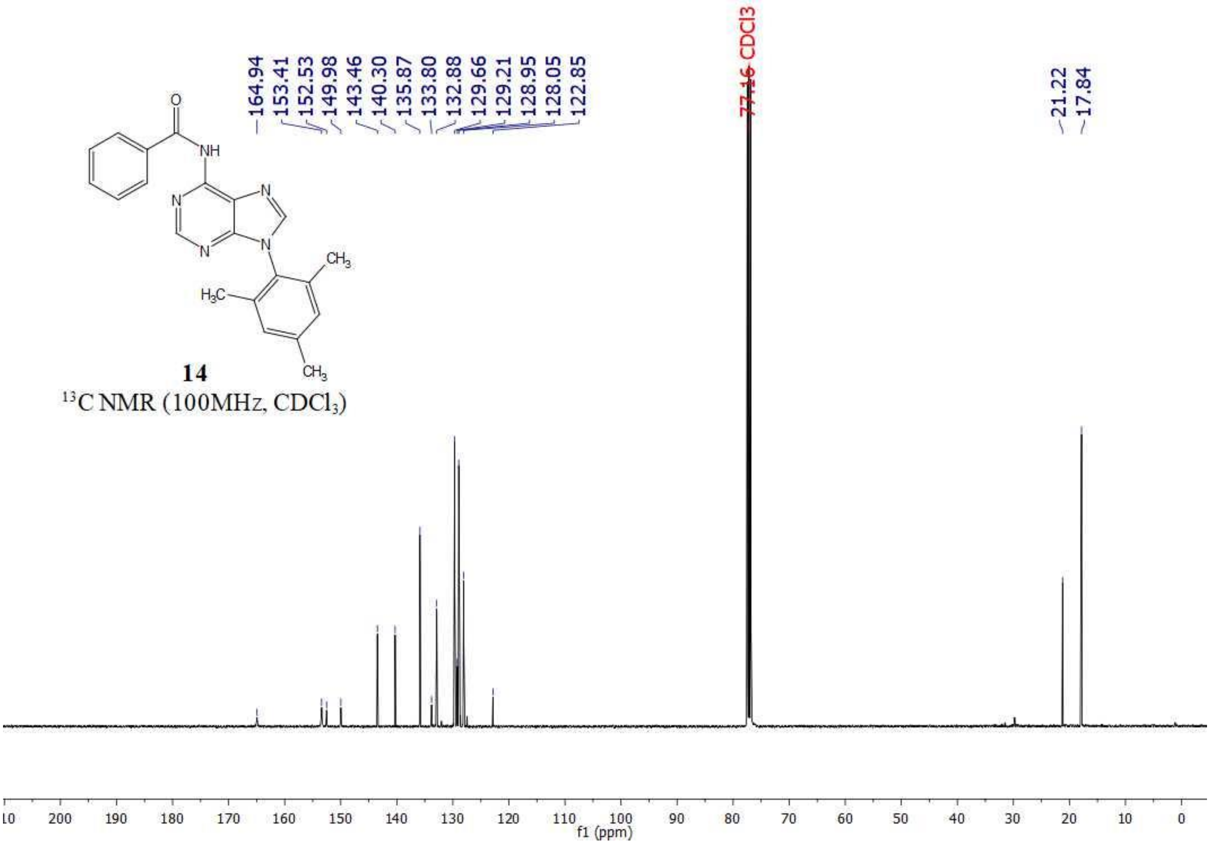

15 - ^{1}H NMR (400 MHz, $CDCl_3$)

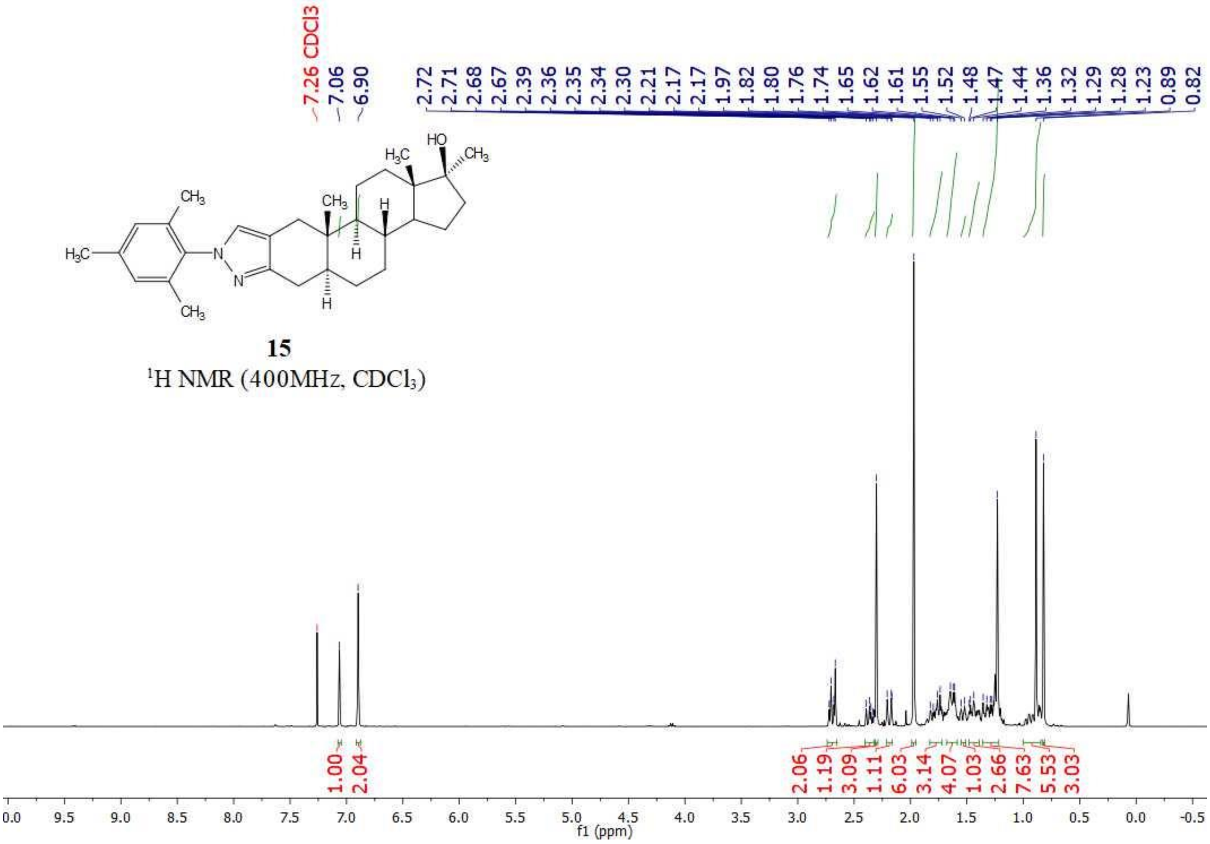

15 – ^{13}C NMR (100 MHz, $CDCl_3$)

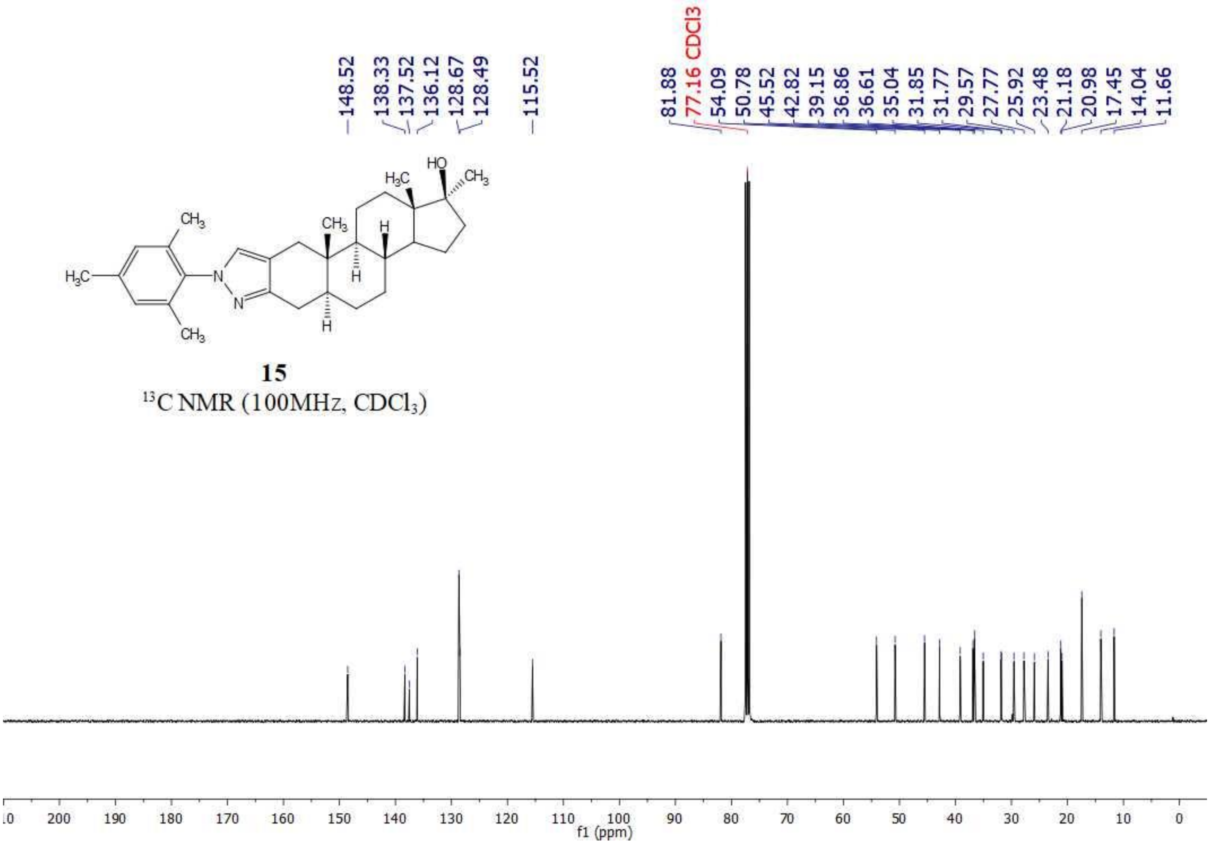

16 - ^{1}H NMR (400 MHz, $CDCl_3$)

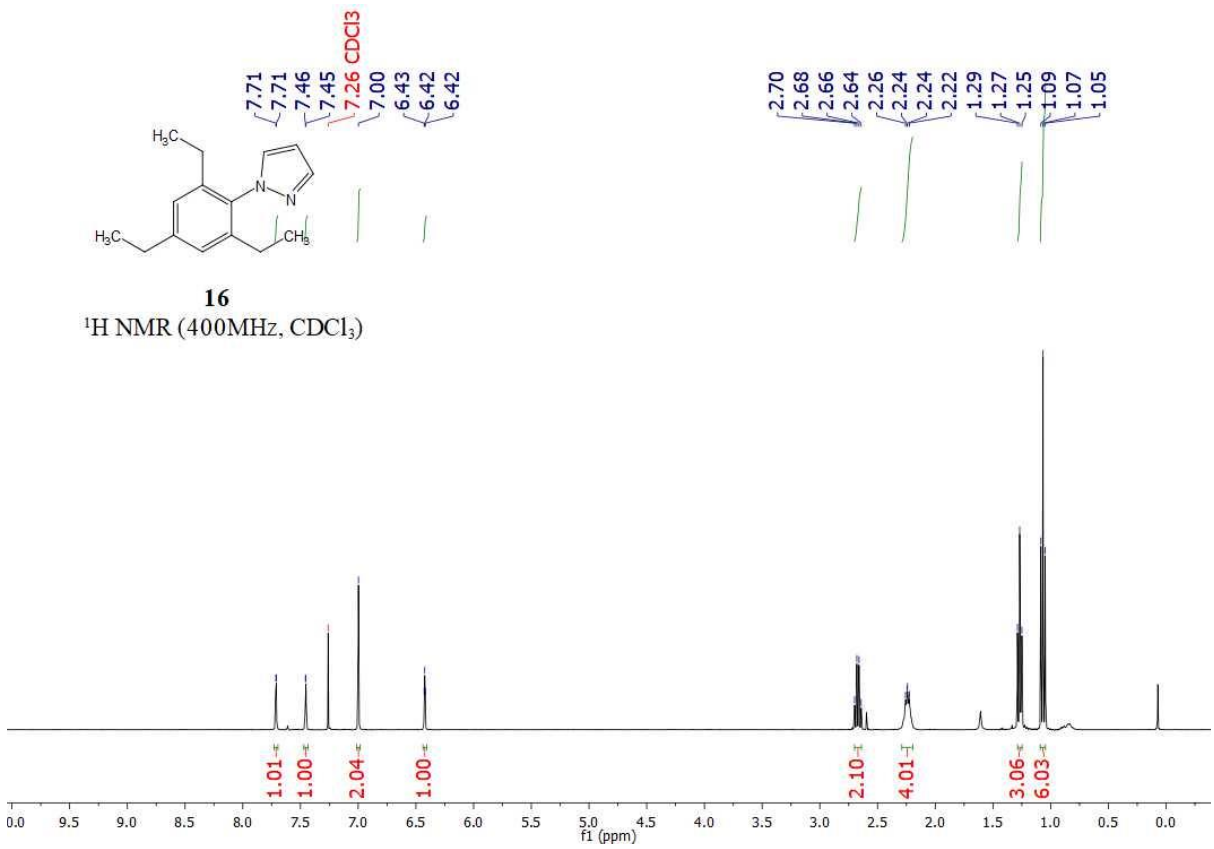

16 – ^{13}C NMR (100 MHz, $CDCl_3$)

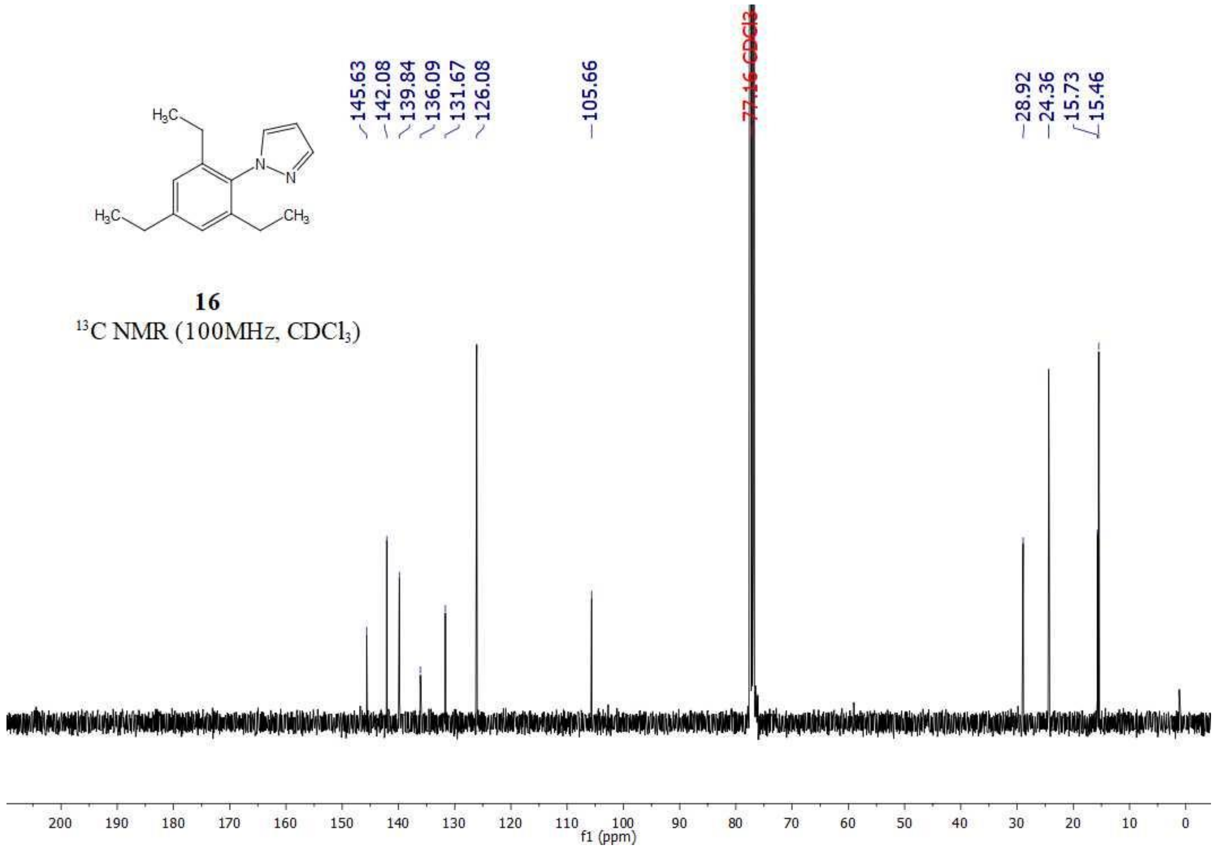

17 - ^{1}H NMR (400 MHz, $CDCl_3$)

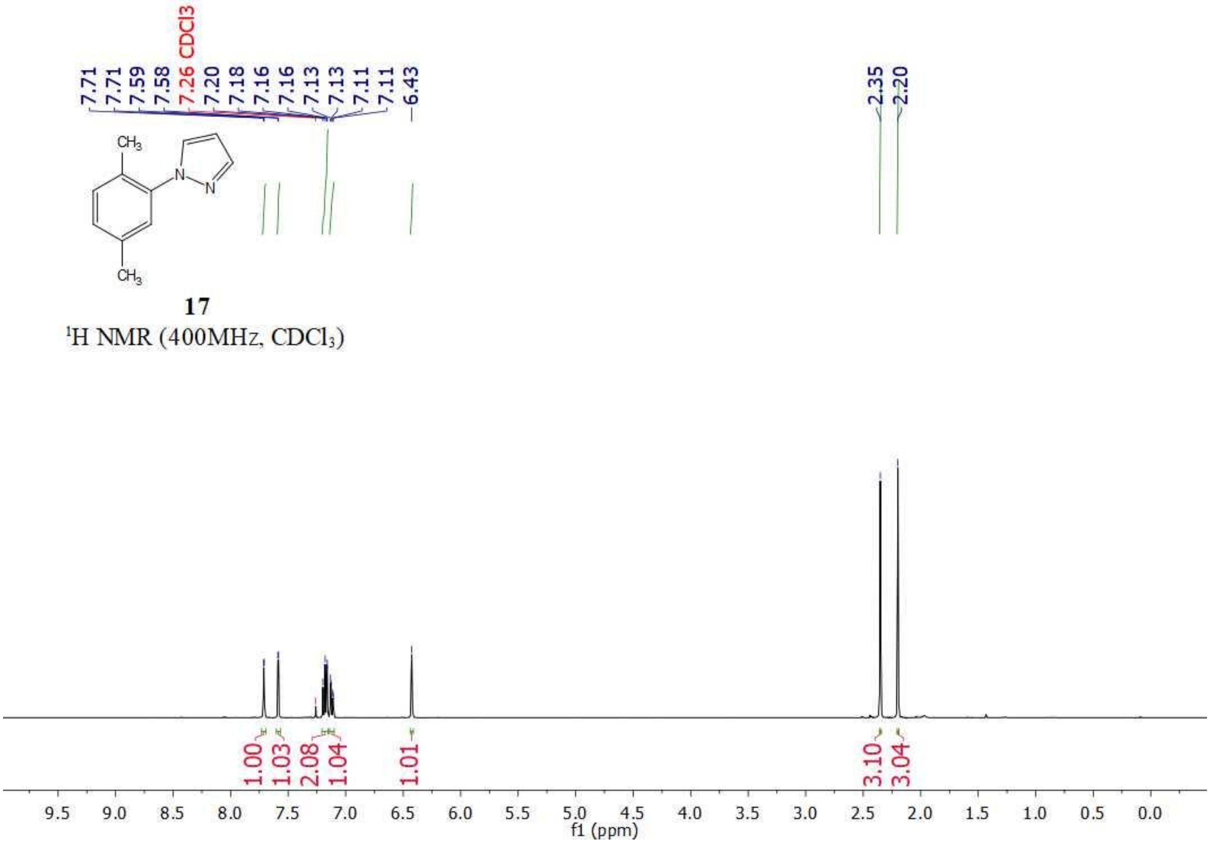

17 – ^{13}C NMR (100 MHz, $CDCl_3$)

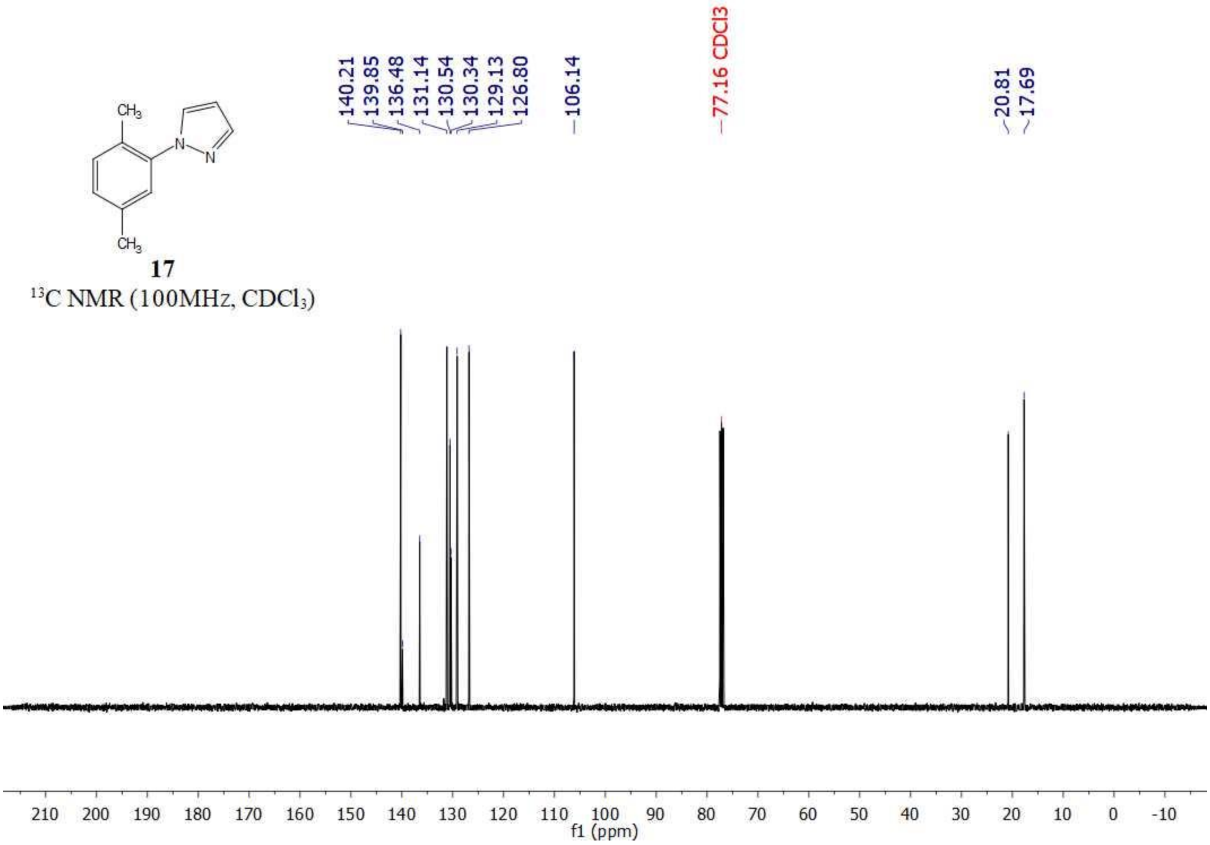

18 - ^{1}H NMR (400 MHz, $CDCl_3$)

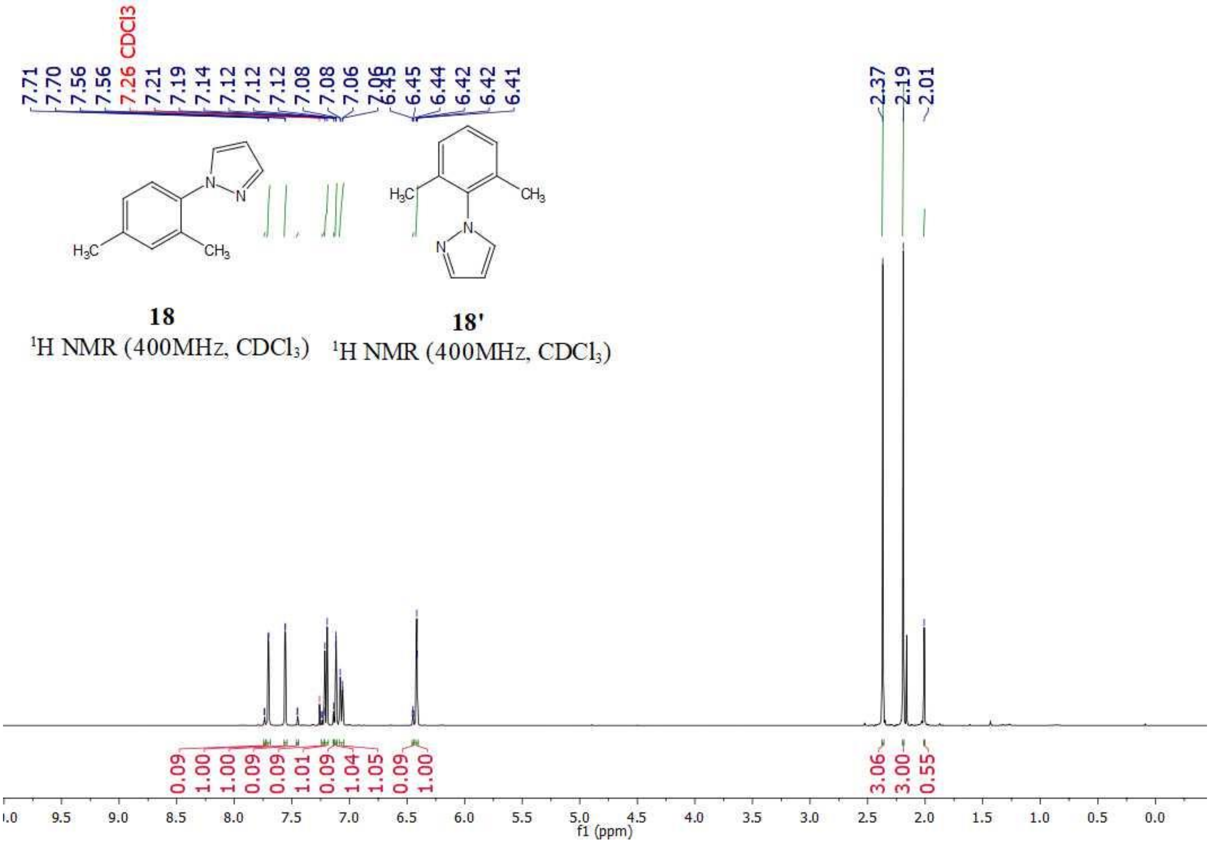

18 – ^{13}C NMR (100 MHz, $CDCl_3$)

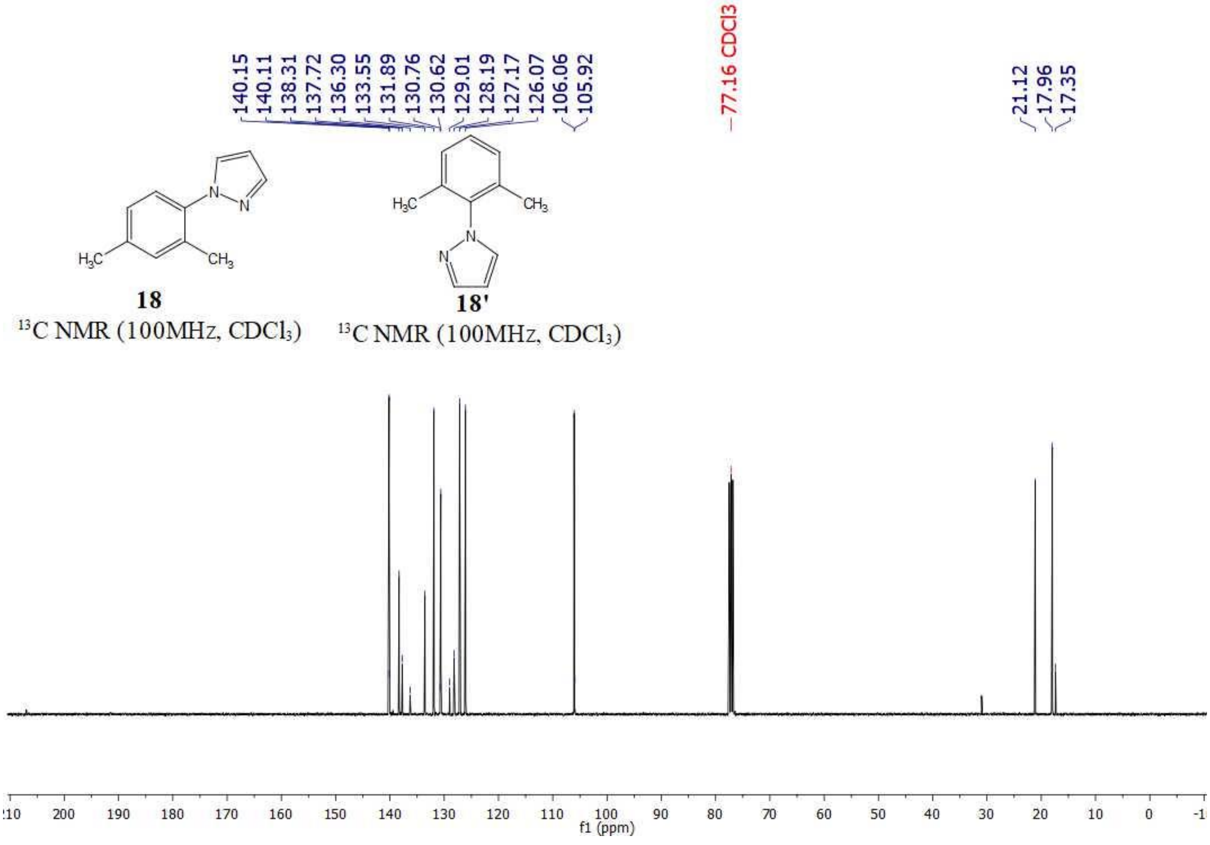

19 - ^{1}H NMR (400 MHz, $CDCl_3$)

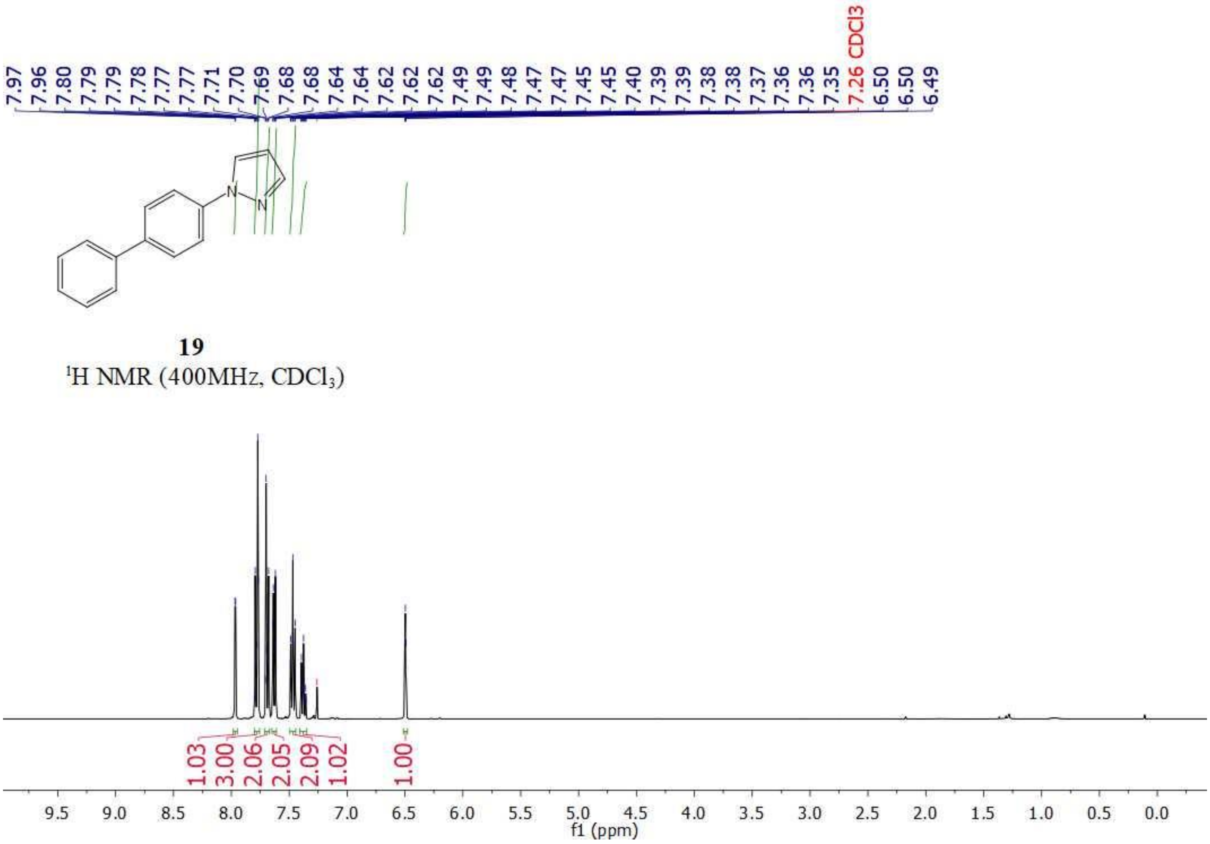

19 – ^{13}C NMR (100 MHz, $CDCl_3$)

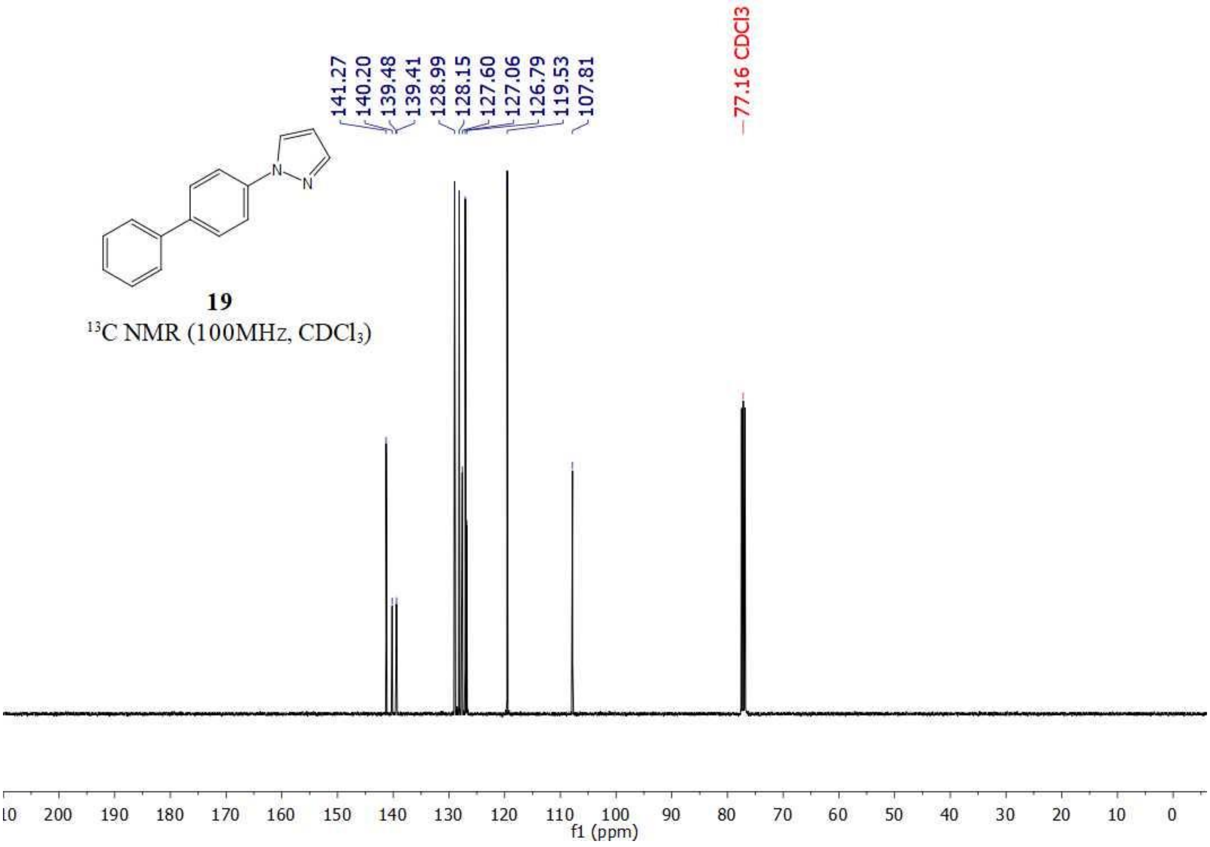

20 - ^{1}H NMR (400 MHz, $CDCl_3$)

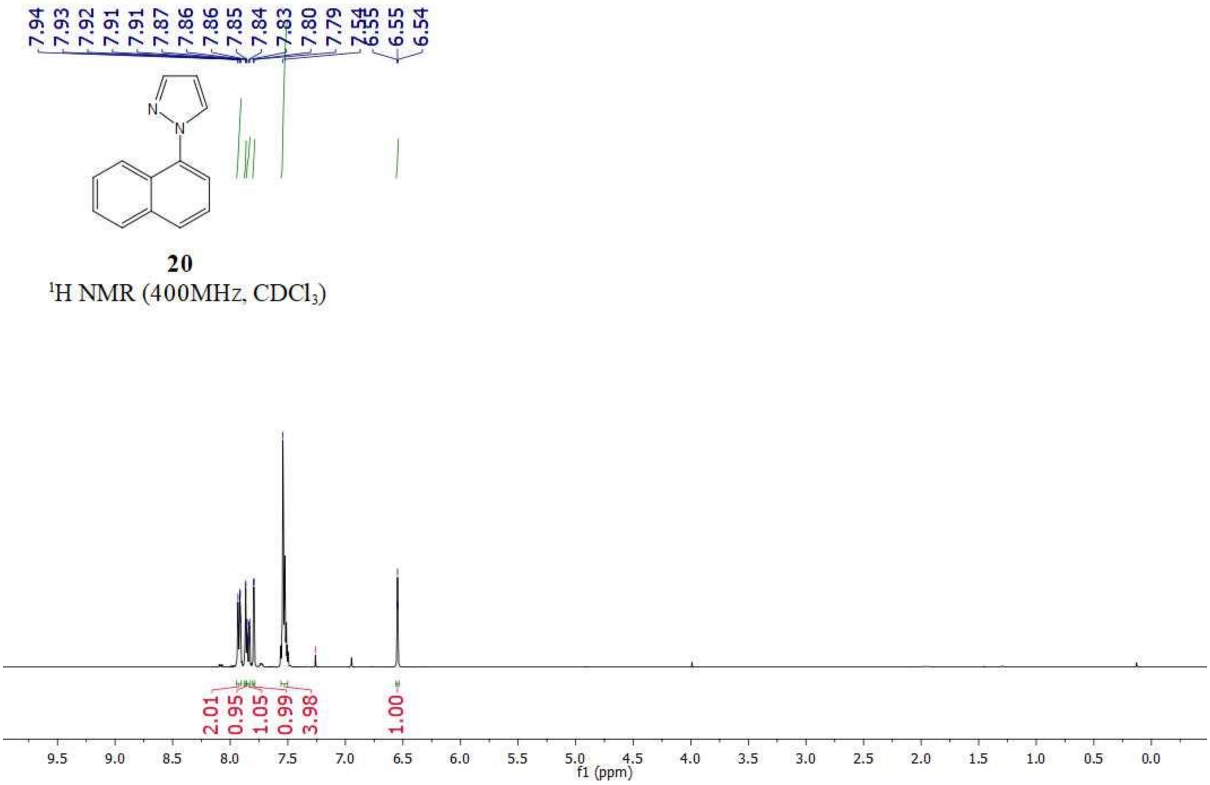

20 – ^{13}C NMR (100 MHz, $CDCl_3$)

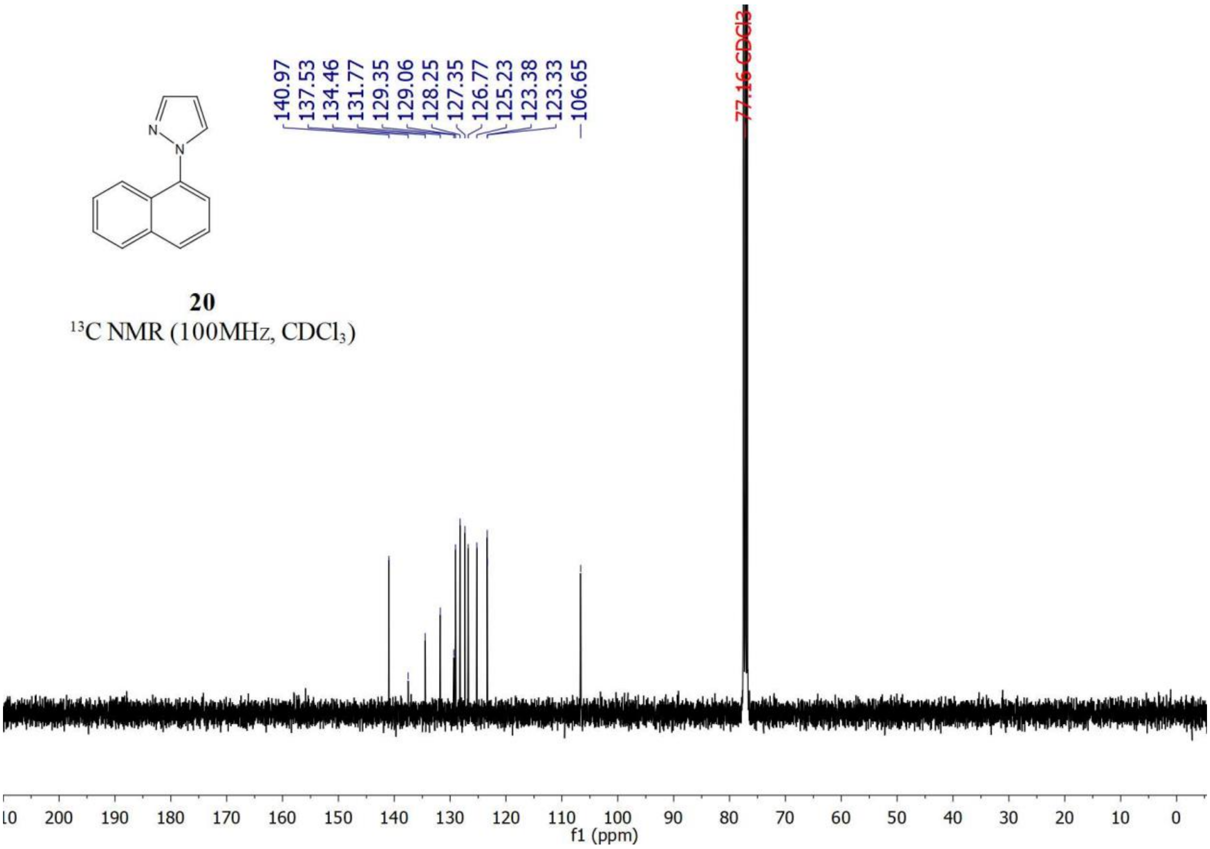

21 - ^{1}H NMR (400 MHz, $CDCl_3$)

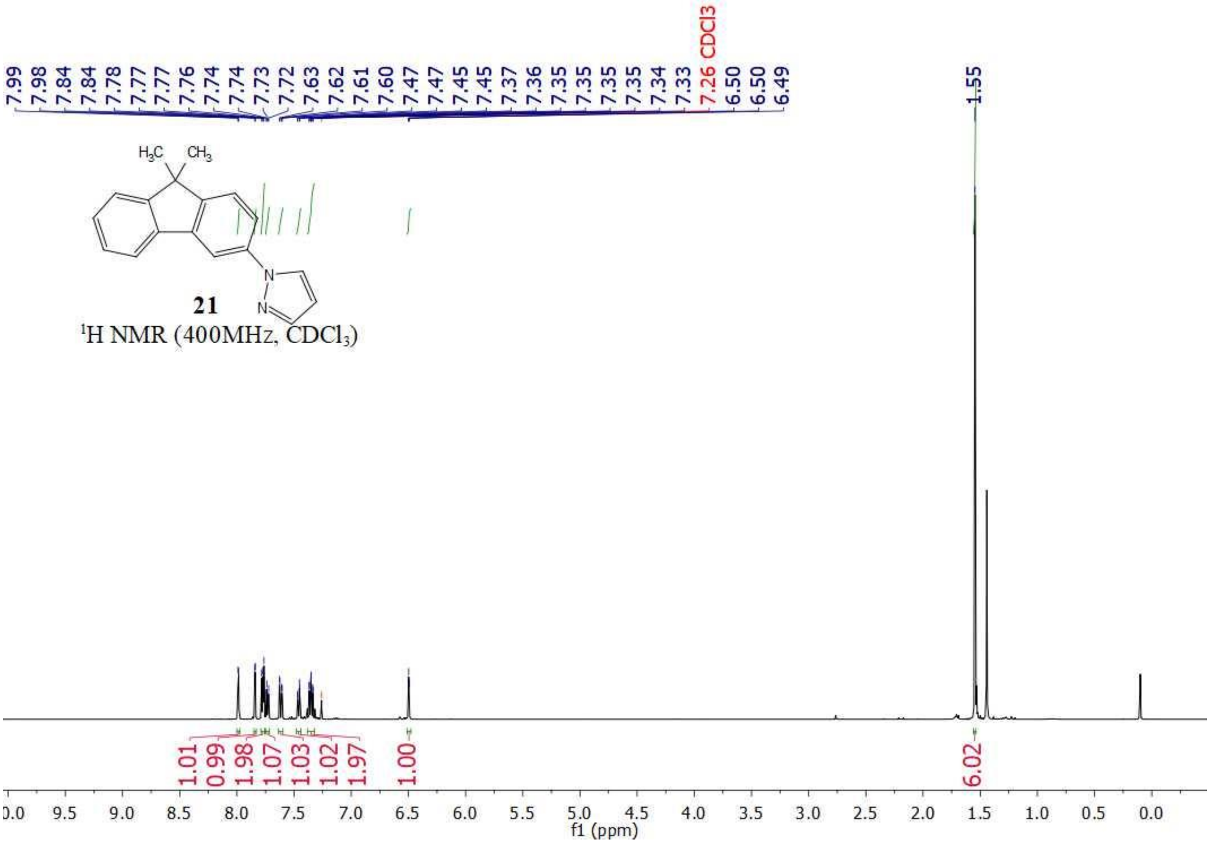

21 – ^{13}C NMR (100 MHz, $CDCl_3$)

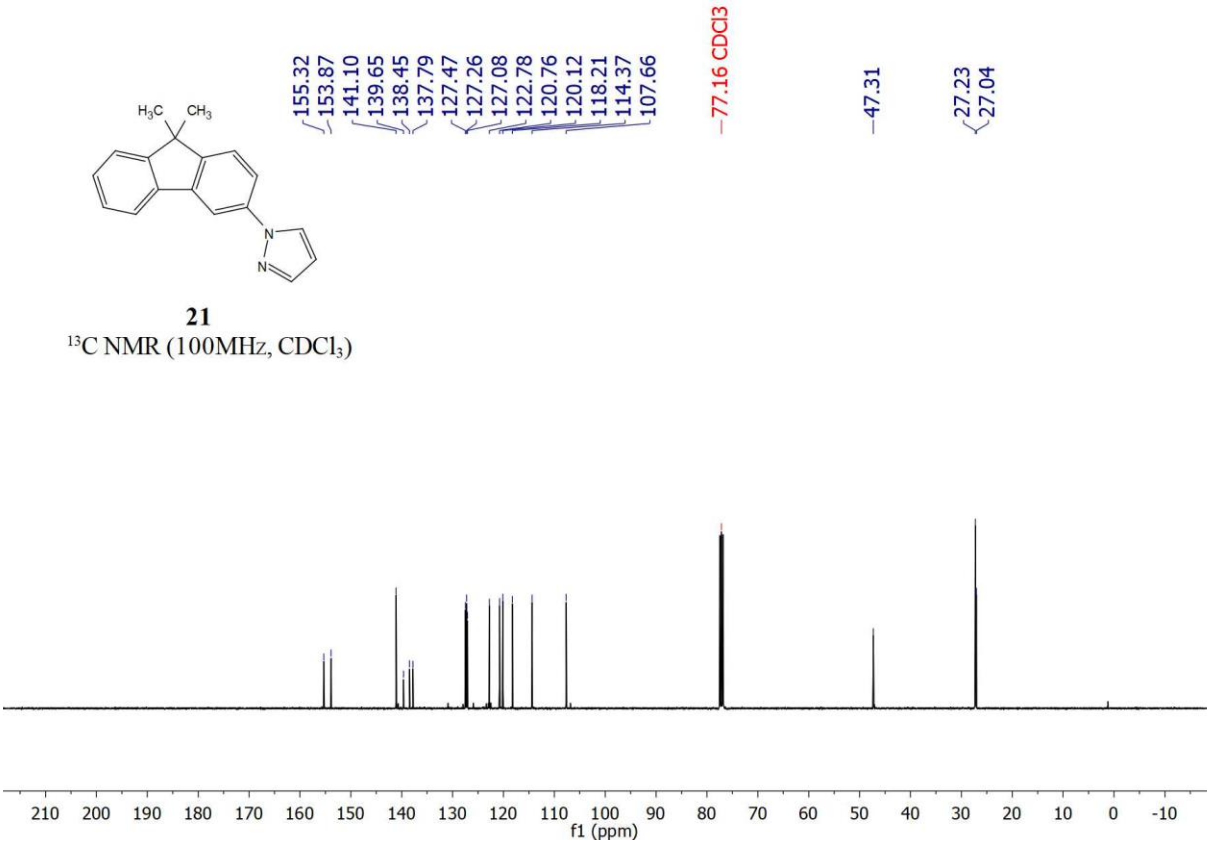

22 - ^{1}H NMR (400 MHz, $CDCl_3$)

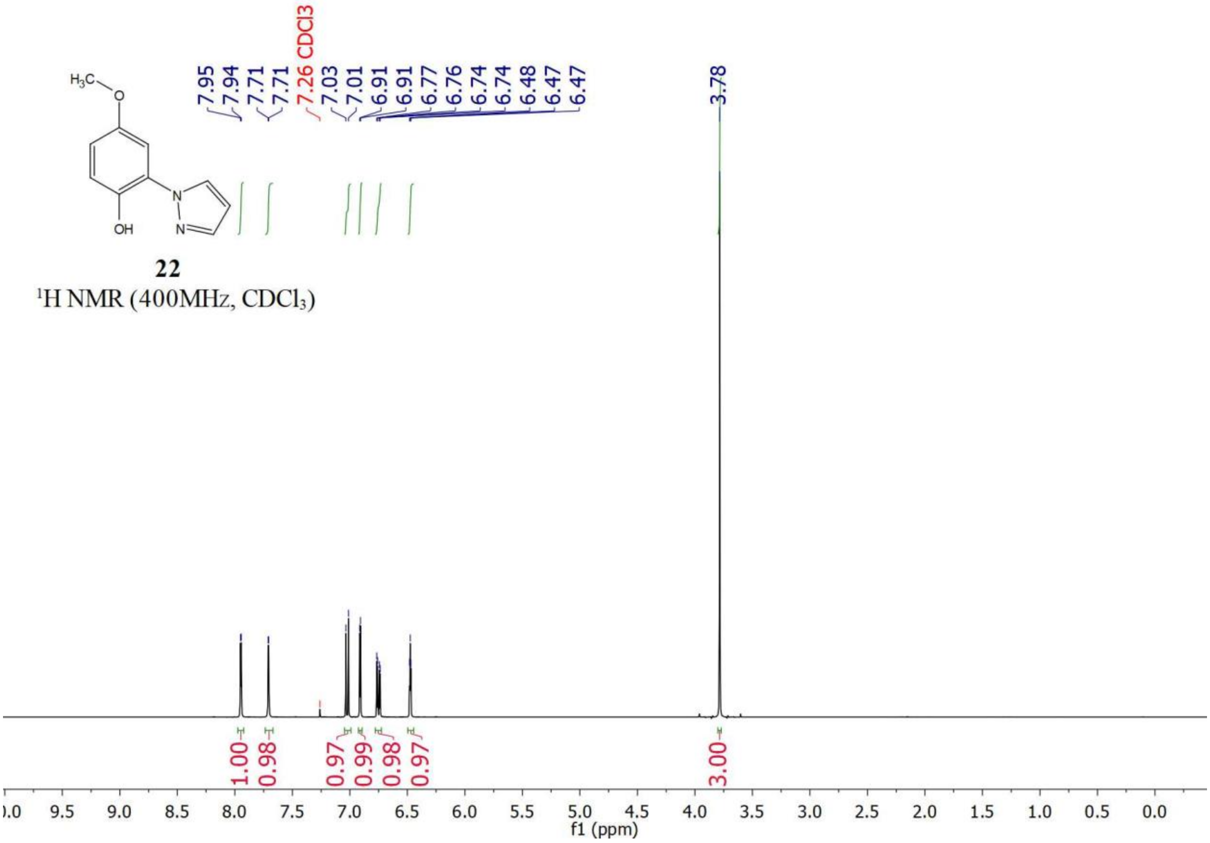

22 – ^{13}C NMR (100 MHz, $CDCl_3$)

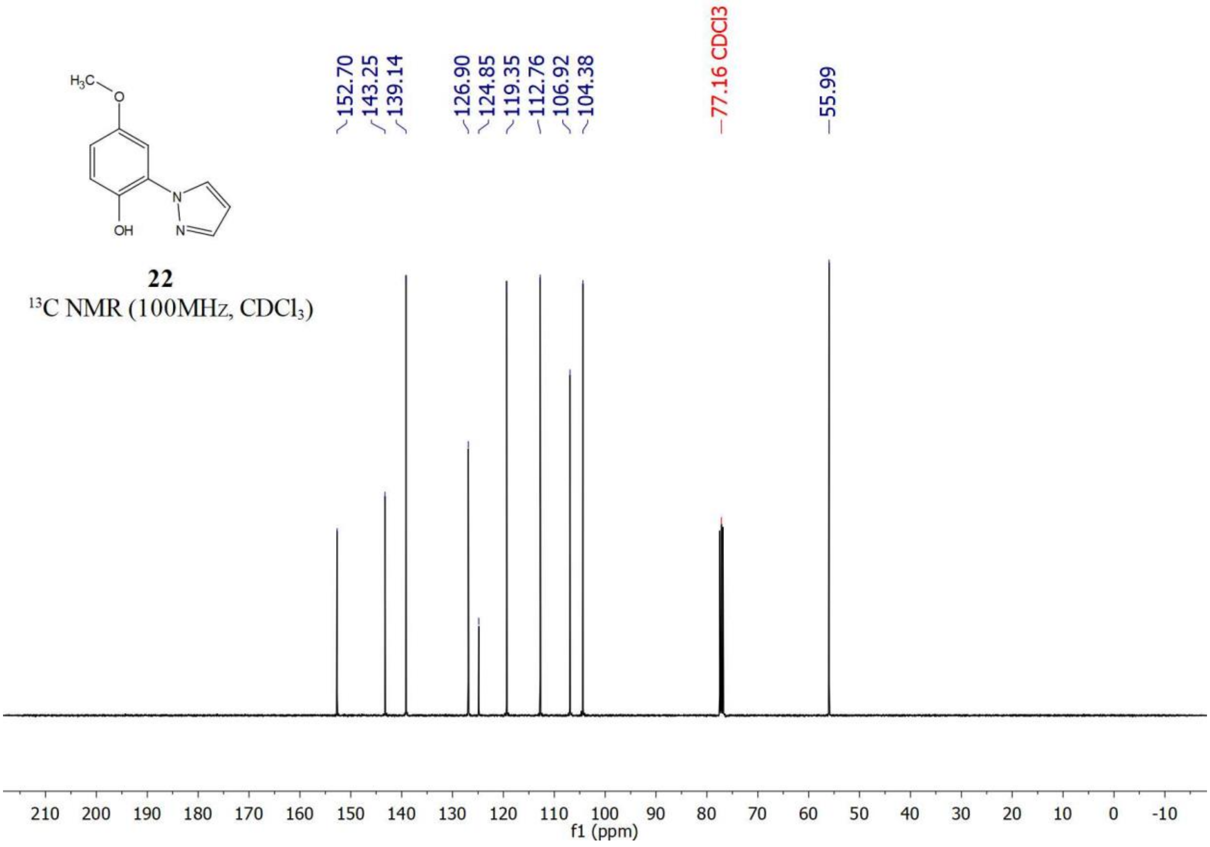

23 - ^{1}H NMR (400 MHz, $CDCl_3$)

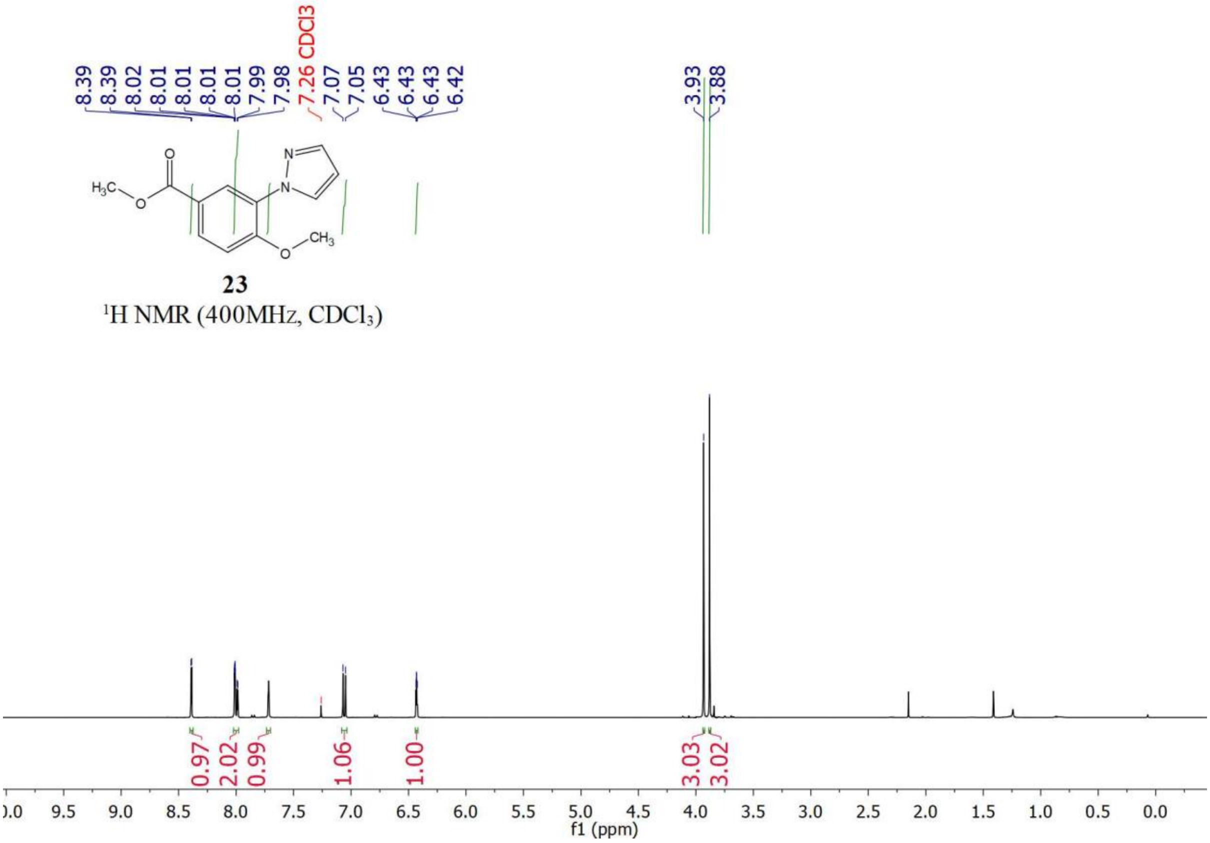

23 – ^{13}C NMR (100 MHz, $CDCl_3$)

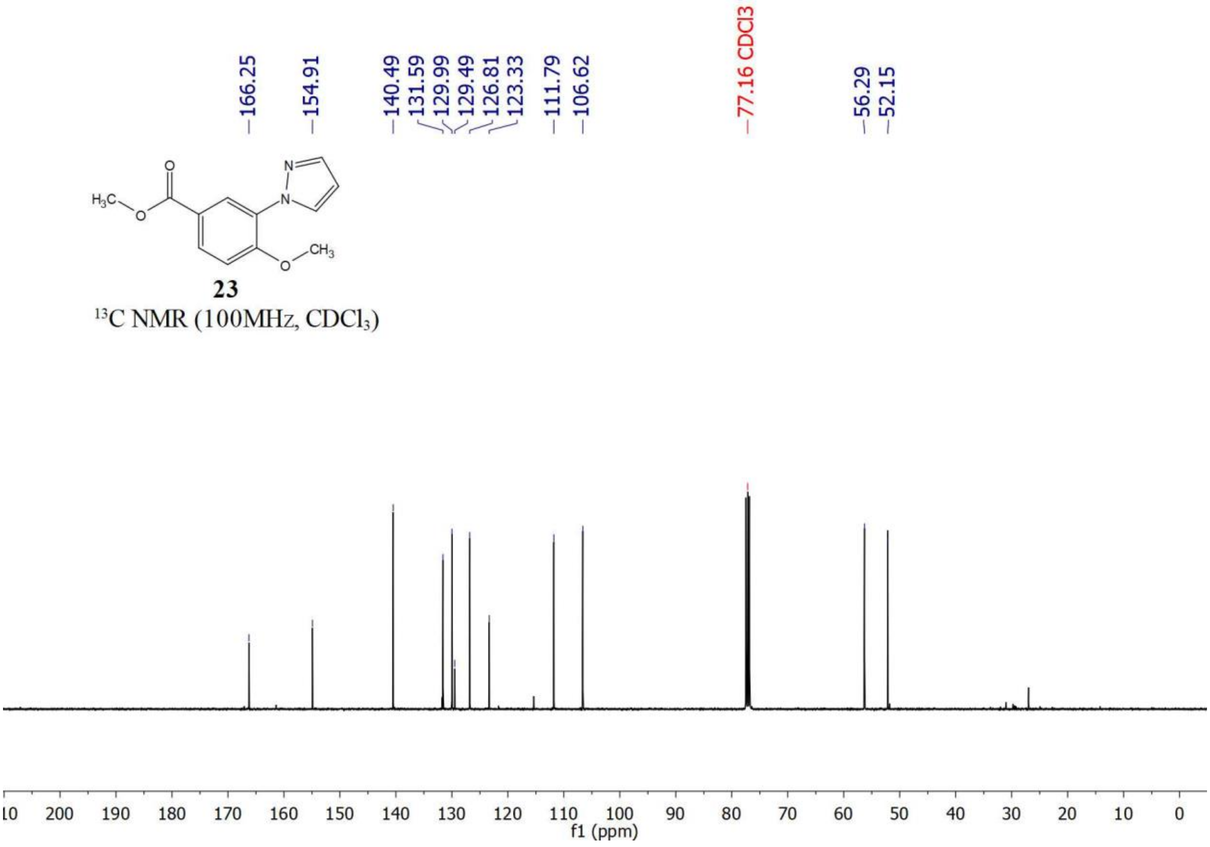

24 - ^{1}H NMR (400 MHz, $CDCl_3$)

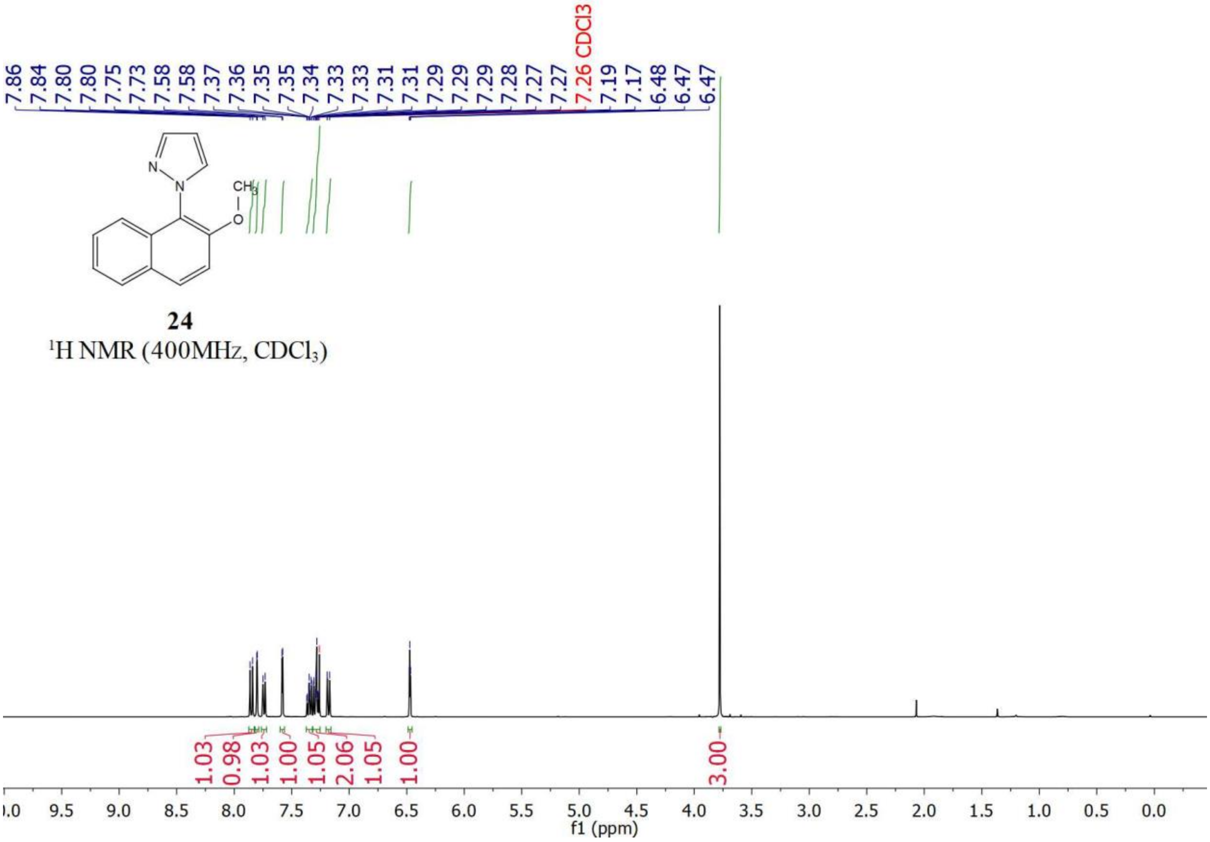

24 – ^{13}C NMR (100 MHz, $CDCl_3$)

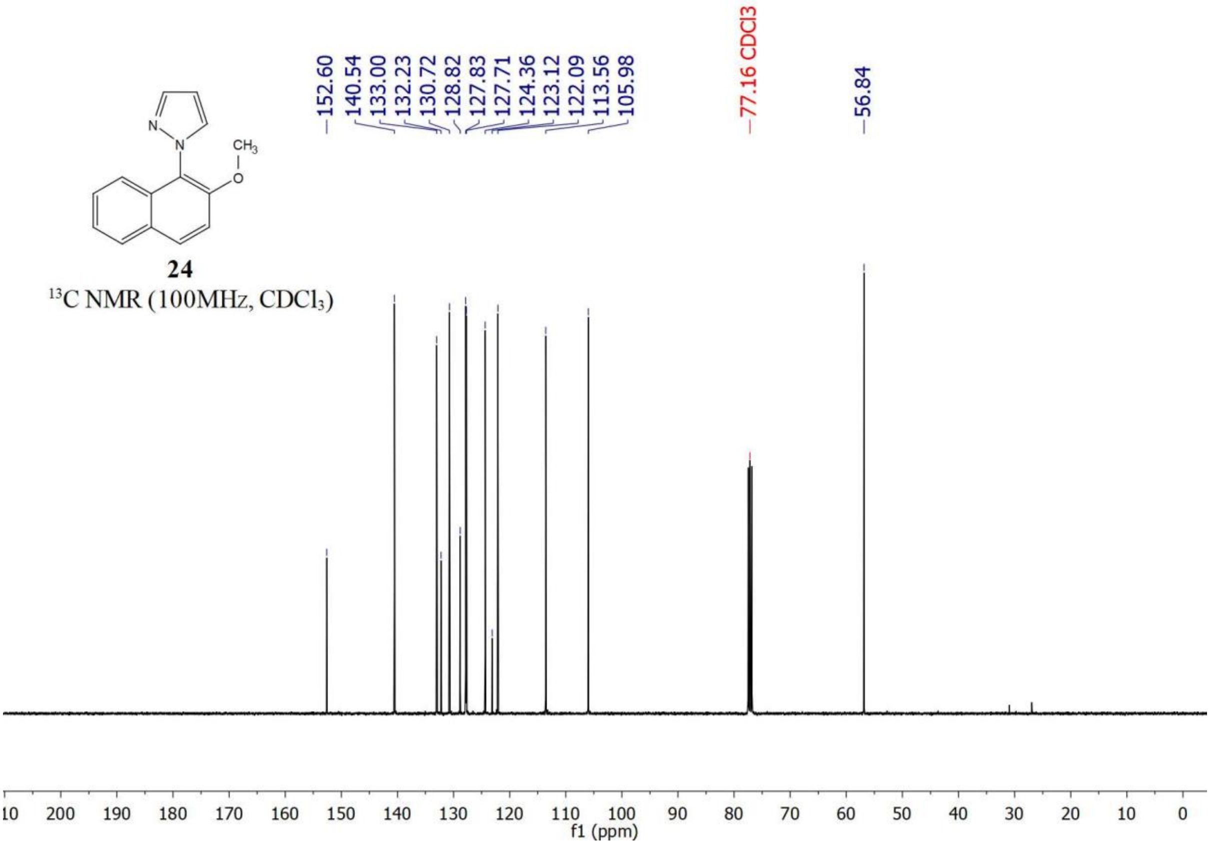

25 - ^{1}H NMR (400 MHz, $CDCl_3$)

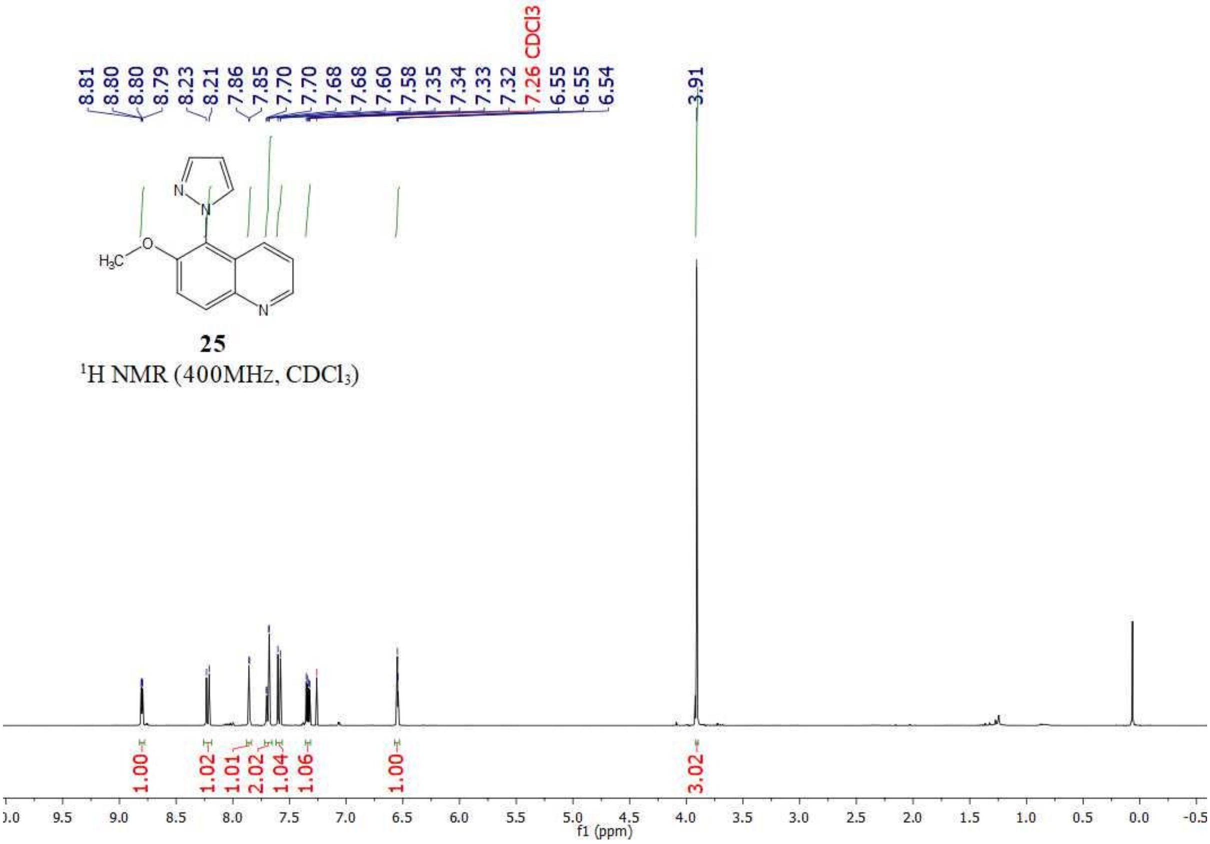

25 – ^{13}C NMR (100 MHz, $CDCl_3$)

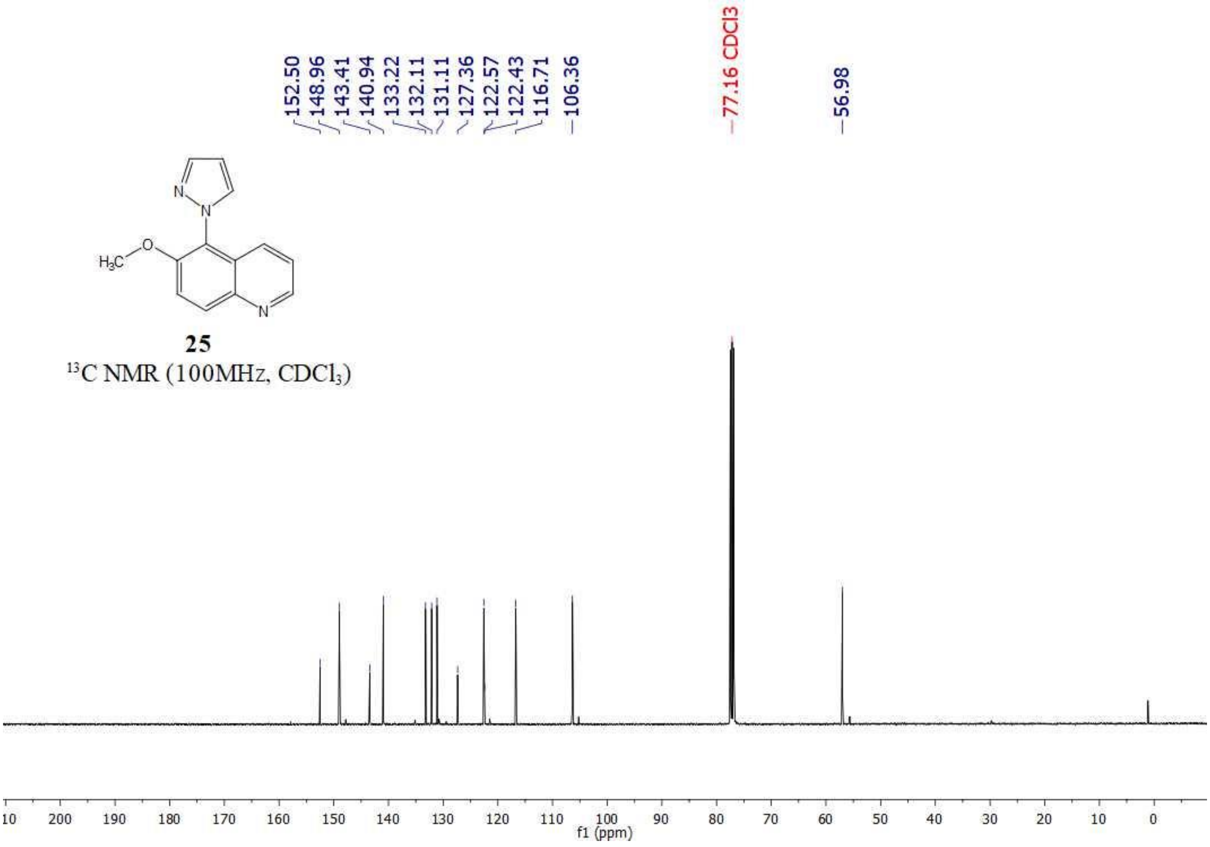

26 - ^{1}H NMR (400 MHz, DMSO-d_6)

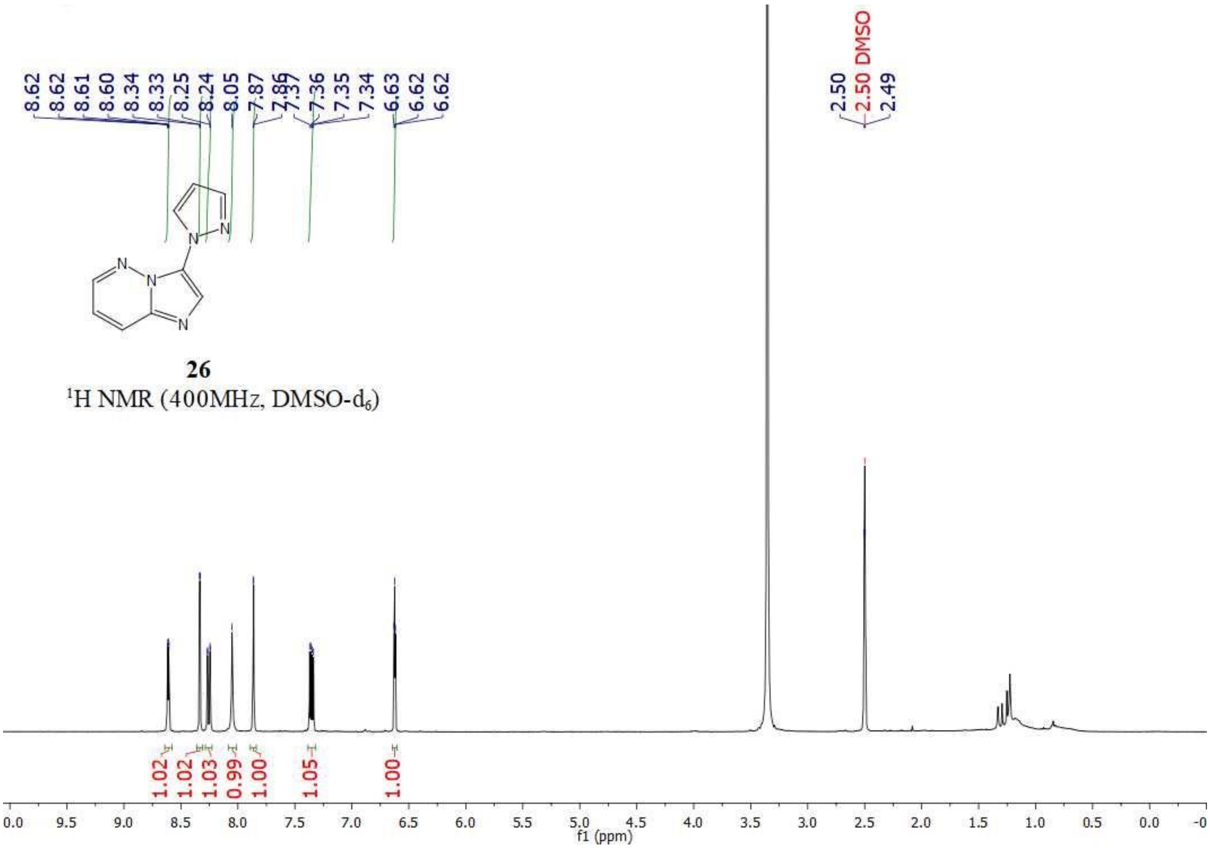

26 – ^{13}C NMR (100 MHz, DMSO-d_6)

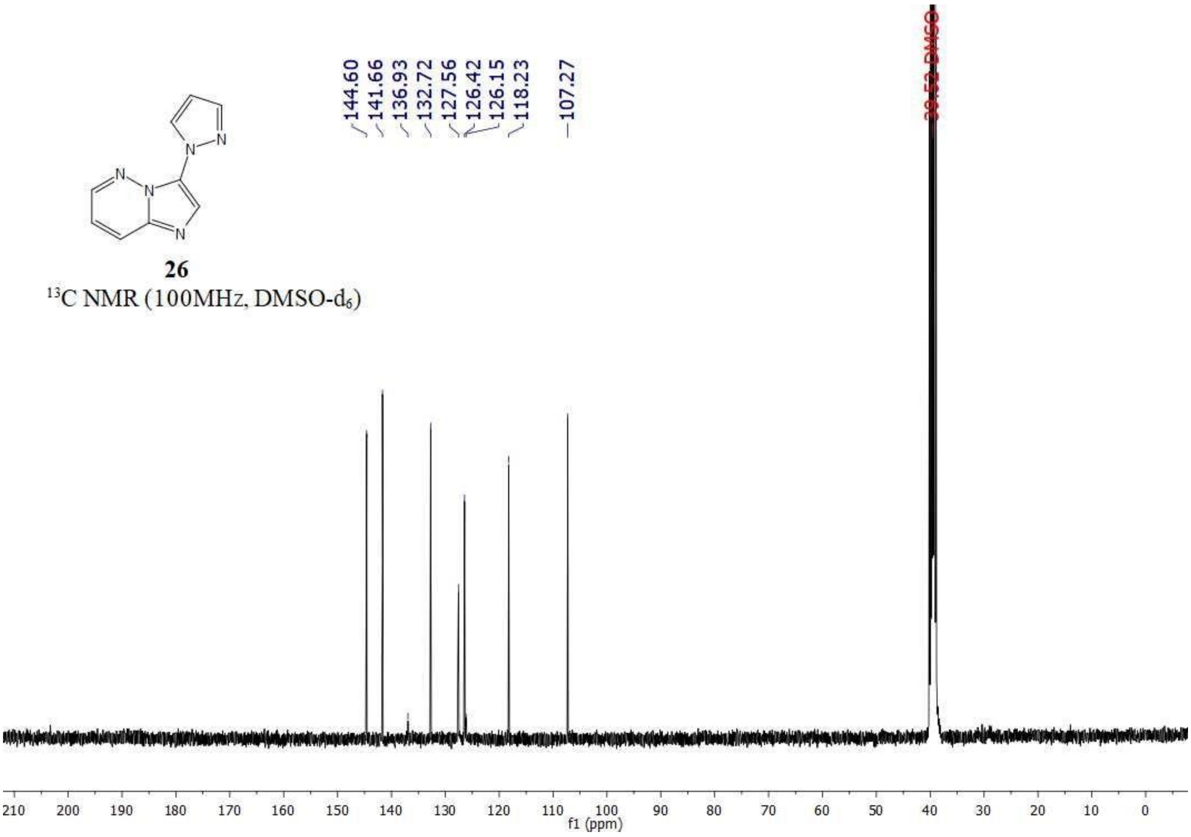

27 - ^{1}H NMR (400 MHz, $CDCl_3$)

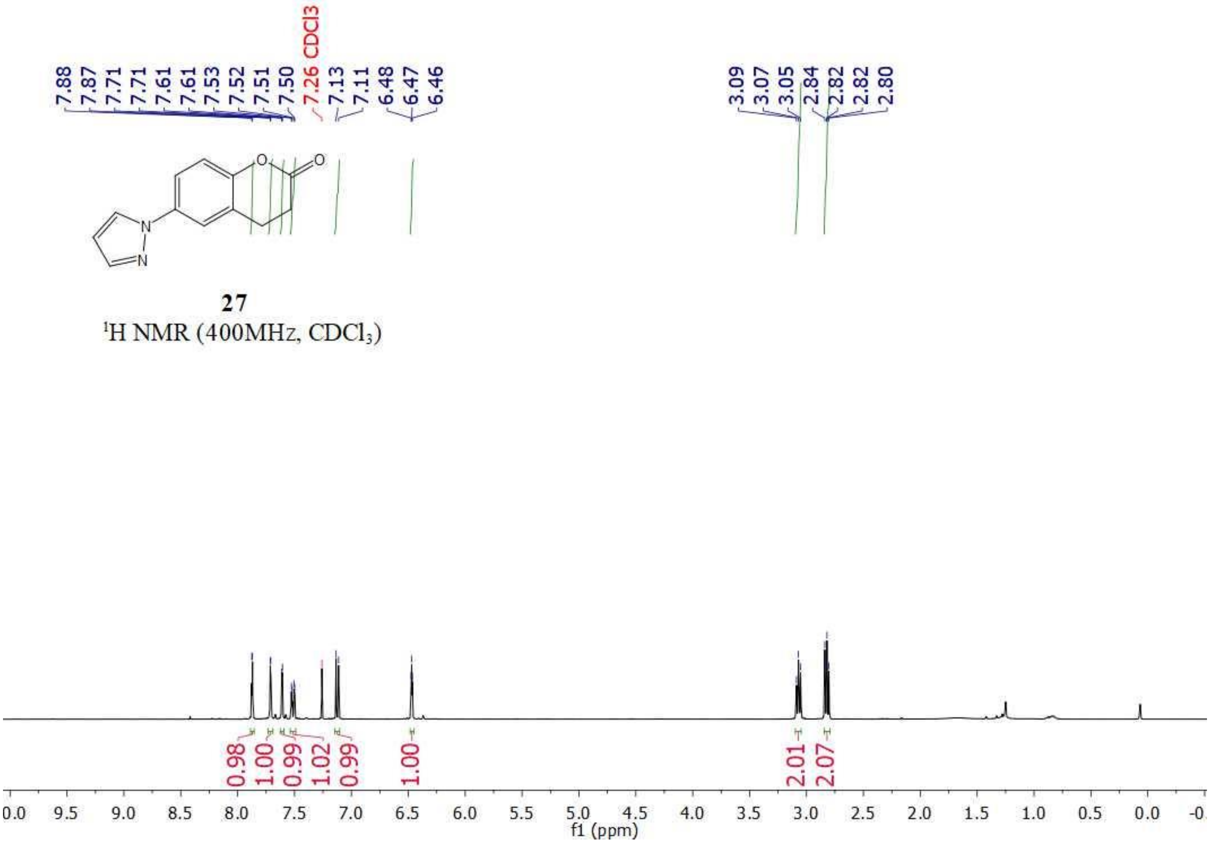

27 – ^{13}C NMR (100 MHz, $CDCl_3$)

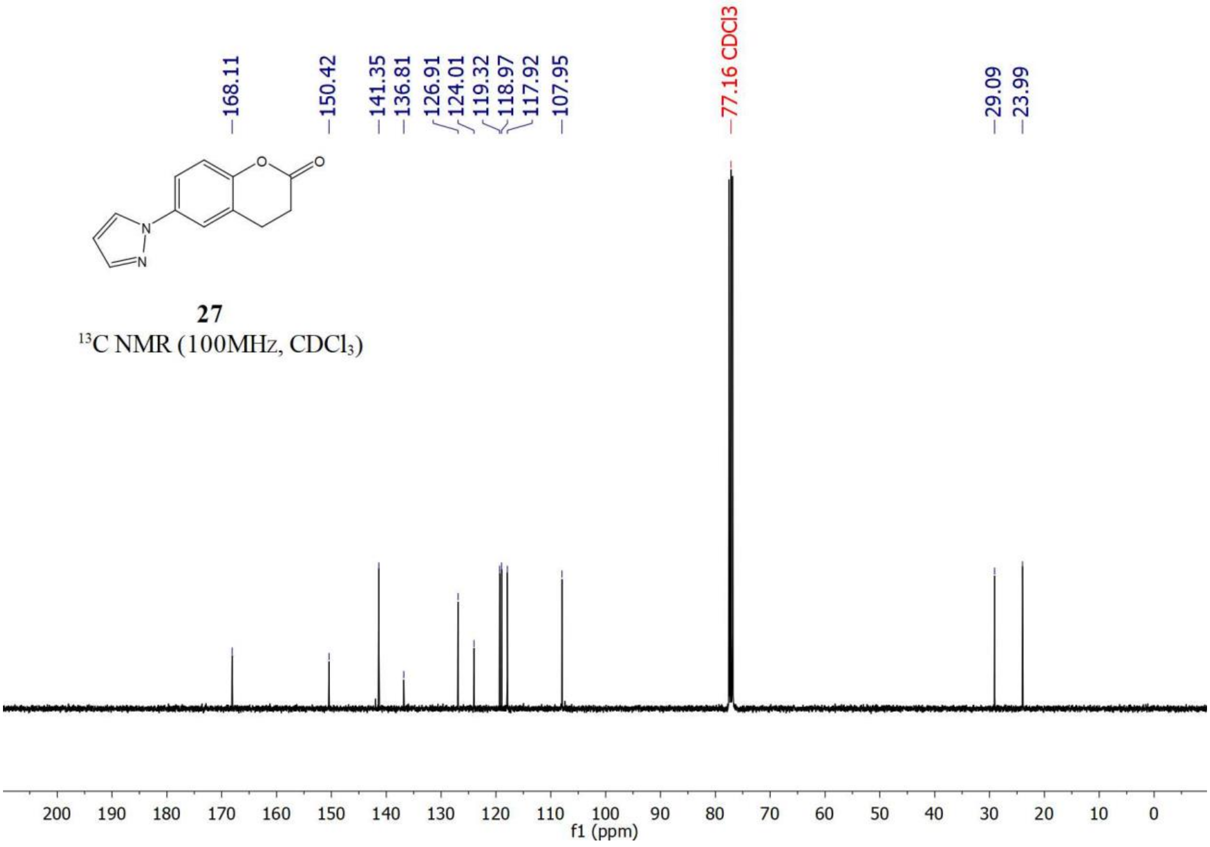

28 - ^{1}H NMR (400 MHz, $CDCl_3$)

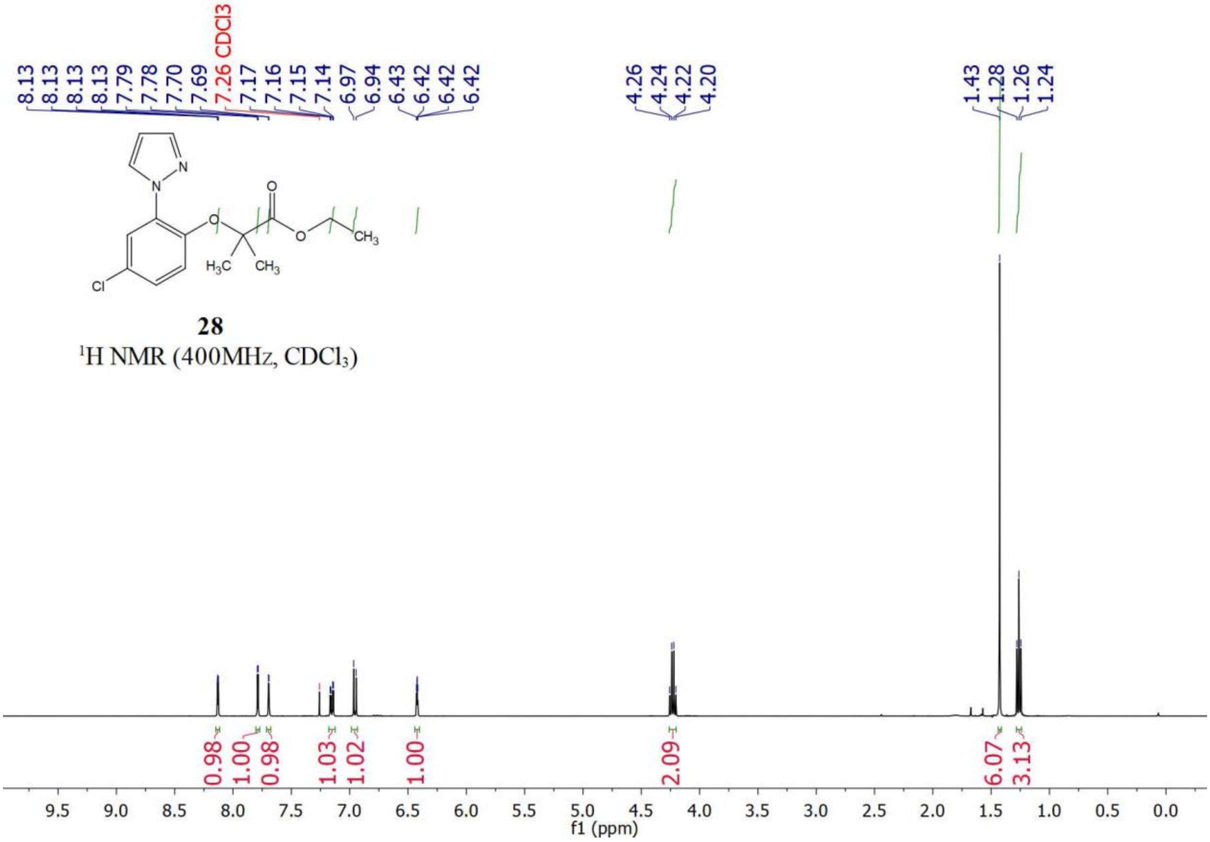

28 – ^{13}C NMR (100 MHz, $CDCl_3$)

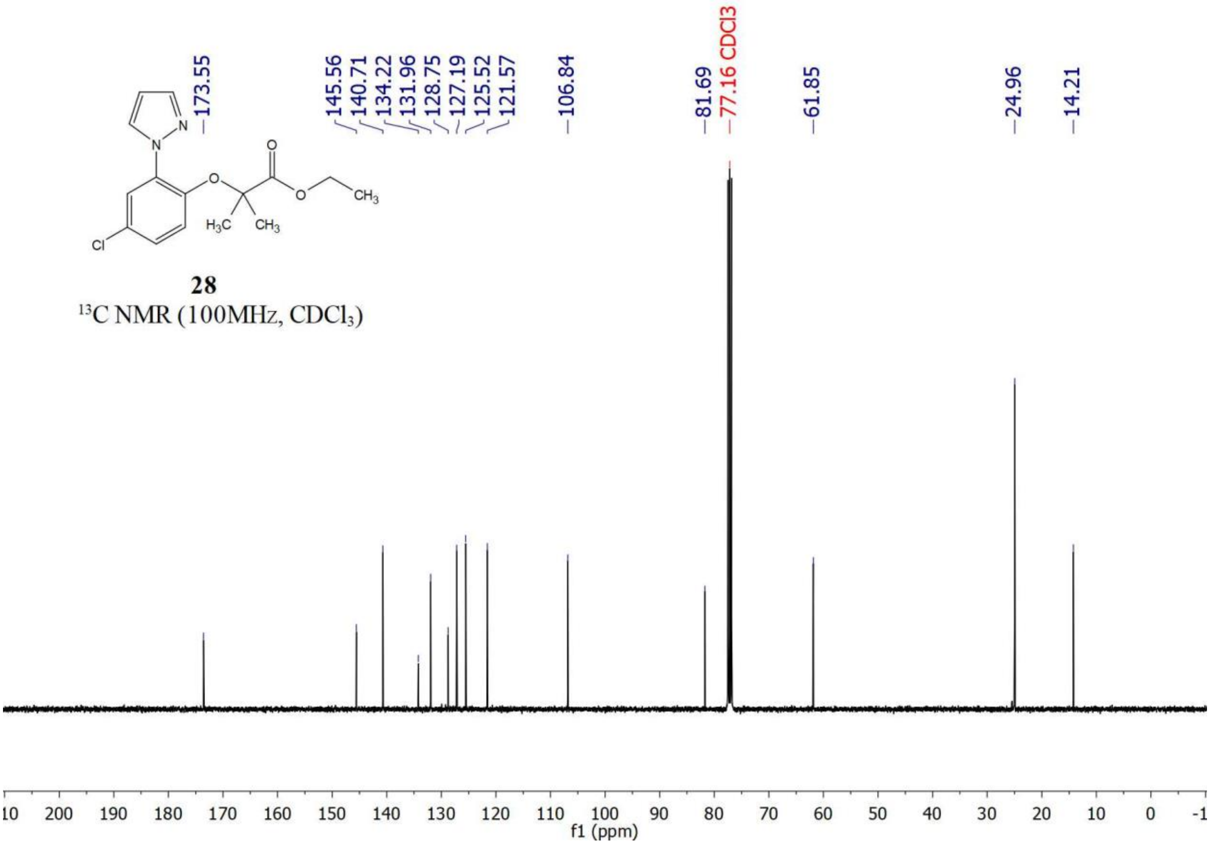

29 - ^{1}H NMR (400 MHz, $CDCl_3$)

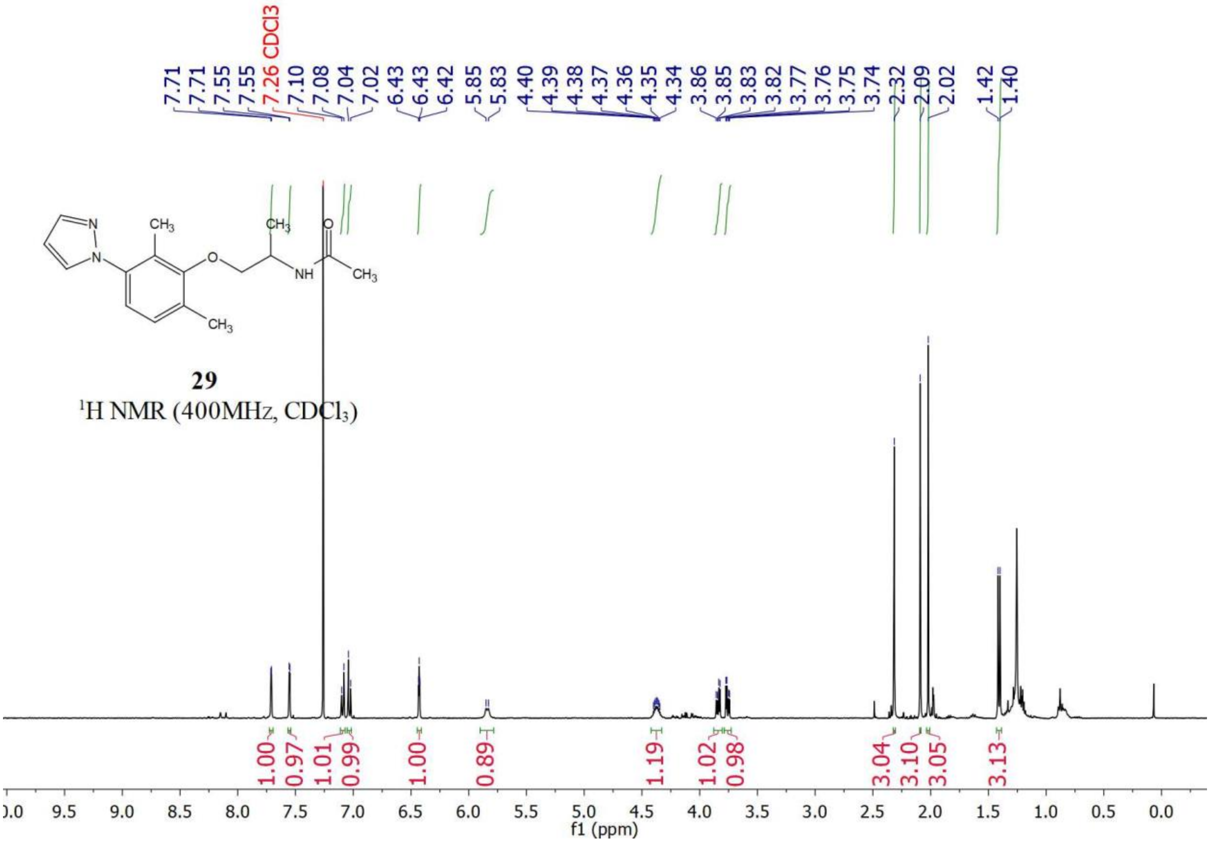

29 – ^{13}C NMR (100 MHz, $CDCl_3$)

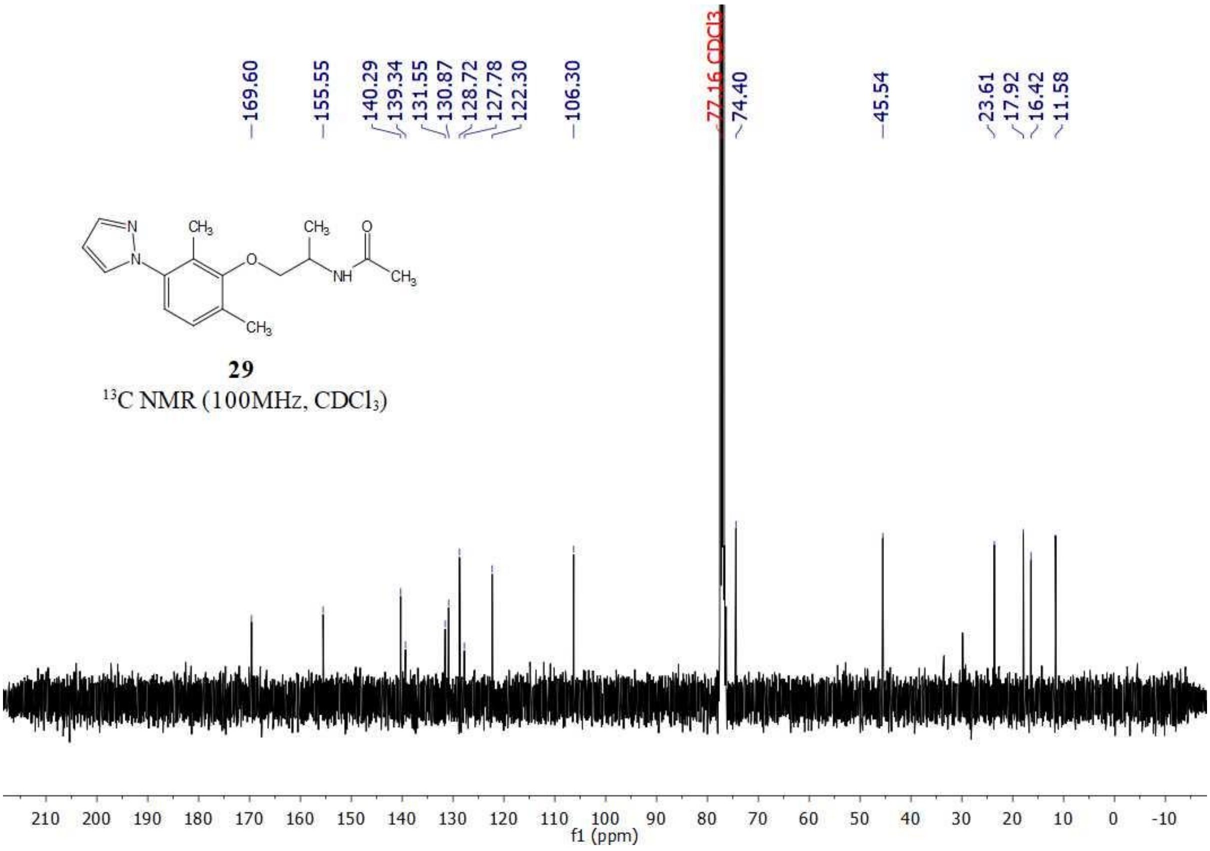

30 - ^{1}H NMR (400 MHz, $CDCl_3$)

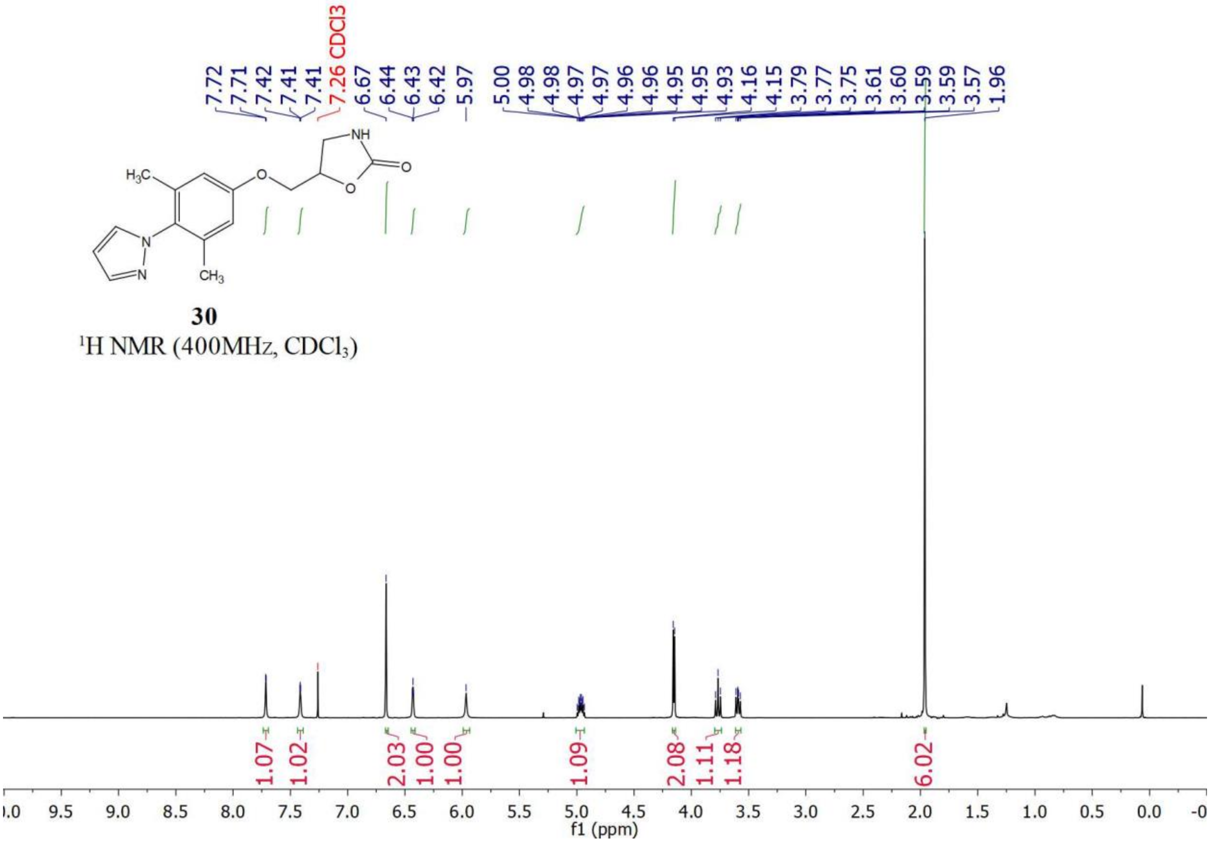

30 – ^{13}C NMR (100 MHz, $CDCl_3$)

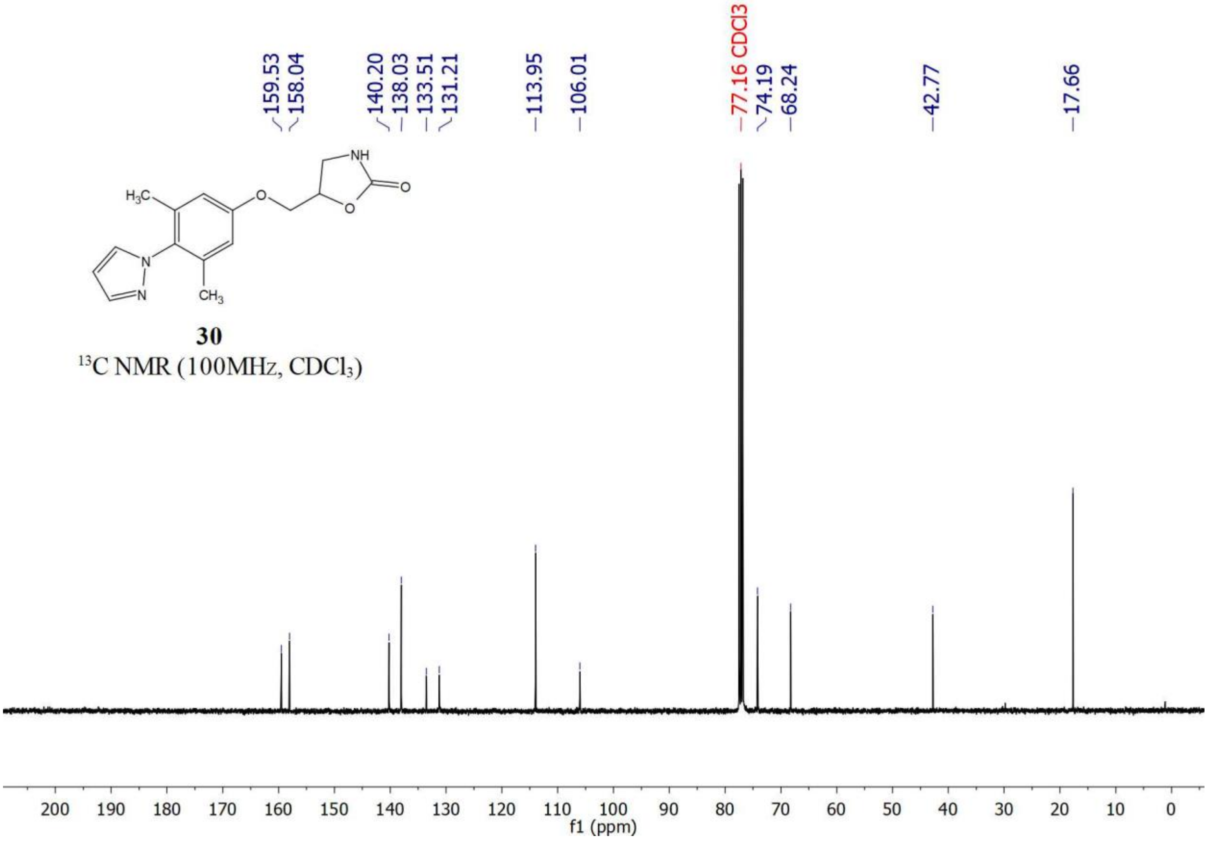

30’ - ^{1}H NMR (400 MHz, $CDCl_3$)

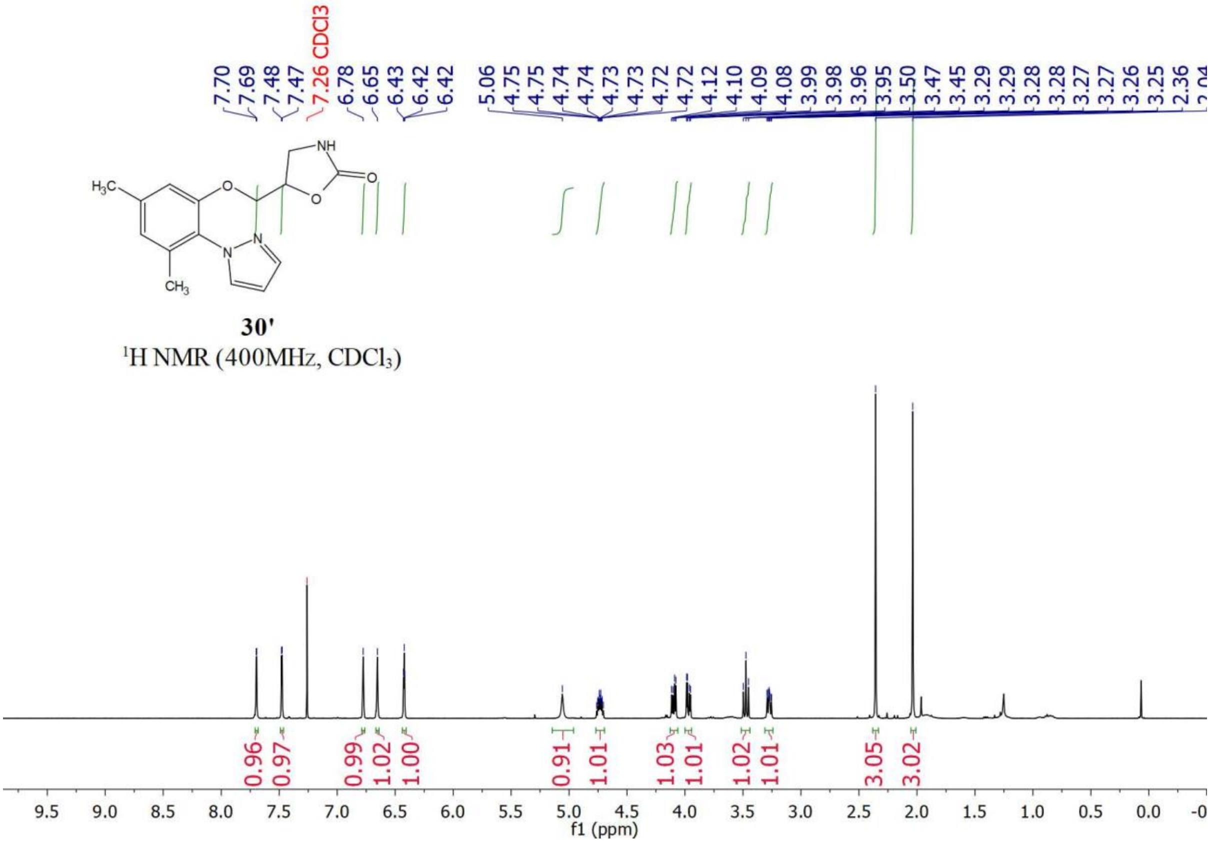

30’ – ^{13}C NMR (100 MHz, $CDCl_3$)

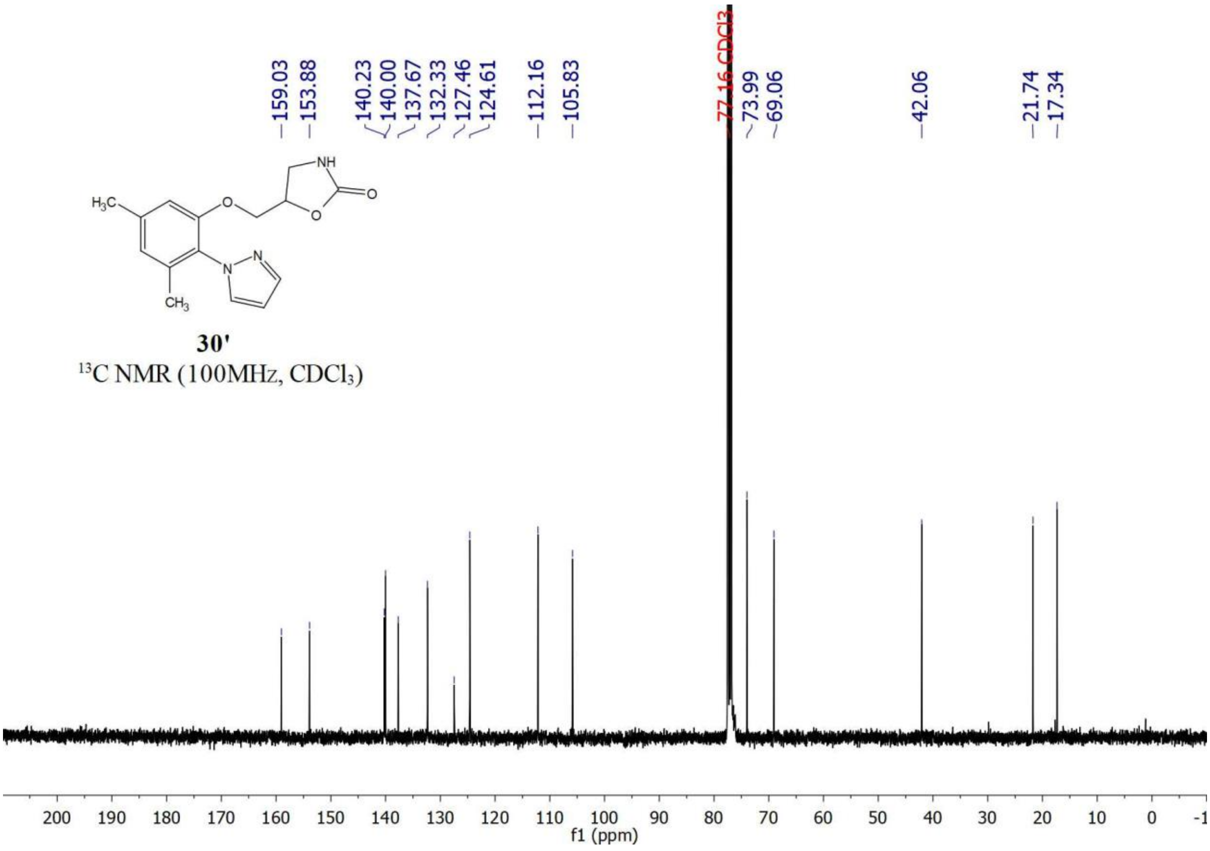

11. Reference

[1] X. Wang, K. Maeda, X. Chen, K. Takanabe, K. Domen, Y. Hou, X. Fu, M. Antonietti, *J. Am. Chem. Soc.* **2009**, *131*, 1680-1681.
[2] M. A. Bajada, A. Vijeta, A. Savateev, G. Zhang, D. Howe, E. Reisner, *ACS Appl. Mater. Interfaces* **2020**, *12*, 8176-8182.
[3] S. Dey, P. Garner, *J. Org. Chem.* **2000**, *65*, 7697-7699.
[4] M. H. Aukland, M. Šiaučiulis, A. West, G. J. P. Perry, D. J. Procter, *Nat. Catal.* **2020**, *3*, 163-169.
[5] N. A. Romero, K. A. Margrey, N. E. Tay, D. A. Nicewicz, *Science* **2015**, *349*, 1326-1330.
[6] Z.-W. Hou, H.-C. Xu, *ChemElectroChem* **2021**, *8*, 1571-1573.
[7] L. Buglioni, M. Beslać, T. Noël, *J. Org. Chem.* **2021**. DOI: https://doi.org/10.1021/acs.joc.1c01409.
[8] a) B. S. Gerstenberger, M. R. Rauckhorst, J. T. Starr, *Org. Lett.* **2009**, *11*, 2097-2100; b) S. Wu, J. Žurauskas, M. Domański, P. S. Hitzfeld, V. Butera, D. J. Scott, J. Rehbein, A. Kumar, E. Thyrhaug, J. Hauer, J. P. Barham, *Org. Chem. Front.* **2021**, *8*, 1132-1142.
[9] V. Zimmermann, S. Bräse, *J. Comb. Chem.* **2007**, *9*, 1114-1137.
[10] L. Niu, H. Yi, S. Wang, T. Liu, J. Liu, A. Lei, *Nat. Commun.* **2017**, *8*, 14226.
[11] K. Swapna, A. Vijay Kumar, V. Prakash Reddy, K. Rama Rao, *J. Org. Chem.* **2009**, *74*, 7514-7517.
[12] P. Feng, G. Ma, X. Chen, X. Wu, L. Lin, P. Liu, T. Chen, *Angew. Chem. Int. Ed.* **2019**, *58*, 8400-8404.
[13] R. A. Novikov, I. P. Klimenko, E. V. Shulishov, V. A. Korolev, Y. V. Tomilov, *Russ. Chem. Bull.* **2008**, *57*, 1718-1724.
[14] K. A. Margrey, J. B. McManus, S. Bonazzi, F. Zecri, D. A. Nicewicz, *J. Am. Chem. Soc.* **2017**, *139*, 11288-11299.
[15] L. Zhang, L. Liardet, J. Luo, D. Ren, M. Grätzel, X. Hu, *Nat. Catal.* **2019**, *2*, 366-373.
